# Inclusion Complexes of Gold(I)‐Dithiocarbamates with β‐Cyclodextrin: A Journey from Drug Repurposing towards Drug Discovery

**DOI:** 10.1002/chem.202101366

**Published:** 2021-07-13

**Authors:** Michael Morgen, Piotr Fabrowski, Eberhard Amtmann, Nikolas Gunkel, Aubry K. Miller

**Affiliations:** ^1^ Cancer Drug Development Group (A390) German Cancer Research Center (DKFZ) Im Neuenheimer Feld 280 69120 Heidelberg Germany; ^2^ German Cancer Consortium (DKTK) 69120 Heidelberg Germany

**Keywords:** gold dithiocarbamates, beta-cyclodextrin, inclusion complexes, host–guest interactions, drug repurposing

## Abstract

The gold(I)‐dithiocarbamate (dtc) complex [Au(*N*,*N*‐diethyl)dtc]_2_ was identified as the active cytotoxic agent in the combination treatment of sodium aurothiomalate and disulfiram on a panel of cancer cell lines. In addition to demonstrating pronounced differential cytotoxicity to these cell lines, the gold complex showed no cross‐resistance in therapy‐surviving cancer cells. In the course of a medicinal chemistry campaign on this class of poorly soluble gold(I)‐dtc complexes, >35 derivatives were synthesized and X‐ray crystallography was used to examine structural aspects of the dtc moiety. A group of hydroxy‐substituted complexes has an improved solubility profile, and it was found that these complexes form 2 : 1 host–guest inclusion complexes with β‐cyclodextrin (CD), exhibiting a rarely observed “tail‐to‐tail” arrangement of the CD cones. Formulation of a hydroxy‐substituted gold(I)‐dtc complex with excess sulfobutylether‐β‐CD prevents the induction of mitochondrial reactive oxygen species, which is a major burden in the development of metallodrugs.

## Graphical abstract

**Inclusion complex of a complex**: The two drugs disulfiram and aurothiomalate react to produce a gold(I)‐dithiocarbamate complex, which selectively kills cancer cells. Hydroxy‐substituted derivatives of this complex form inclusion complexes with β‐cyclodextrin in a rare “tail‐to‐tail” arrangement. Formulation of one gold complex with a β‐cyclodextrin derivative prevents formation of reactive oxygen species in mitochondria.

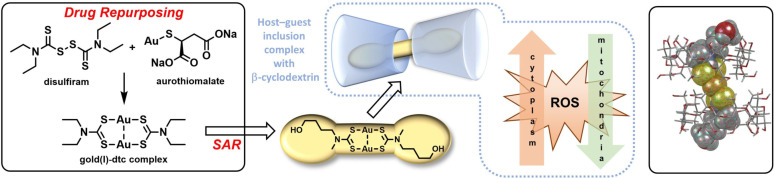

## Introduction

**Figure 1:**
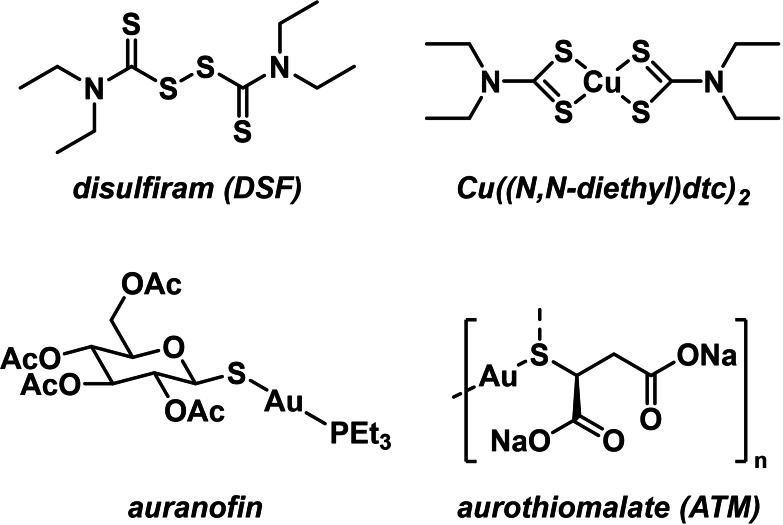
Metal‐containing and metal‐coordinating drugs.

Drug repurposing, the development of approved or clinically investigated drugs for new indications, is a powerful approach to bring new therapy options to patients.^[1]^ By harnessing the world's vast chemical equity for which toxicological and pharmacokinetic data in human subjects already exists, drug repurposing can progress compounds from pre‐clinical proof of concept studies to Phase 2 clinical trials more quickly and economically than traditional drug development projects. One substance that has been investigated extensively for repurposing in oncology is disulfiram (DSF), a drug which is clinically used to treat alcohol dependence (Figure 1).^[2]^ In a seminal retrospective epidemiological study, Skrott *et al*. found a significant benefit for cancer patients who continued to take DSF after their diagnosis.^[3]^ Interestingly, the researchers demonstrated that disulfiram's anti‐cancer activity derives not from disulfiram itself, but from the copper dithiocarbamate (dtc) complex Cu((*N,N*‐diethyl)dtc)_2_, which forms *in vivo* with endogenous or supplemented Cu^2+^ (Figure 1). The development of metal‐containing drugs remains a vibrant area of research, and we found the idea of forming organometallic complexes *in vivo* a unique opportunity for drug repurposing. Among the late transition metals, gold has a long history in medicine and is found in approved drugs like aurothiomalate (ATM)^[4]^ and auranofin. As gold complexes are widely regarded to be promising anti‐cancer agents,^[5]^ we wondered what the effects from combined administration of DSF and ATM would be.^[6]^

## Results and Discussion

**Figure 2:**
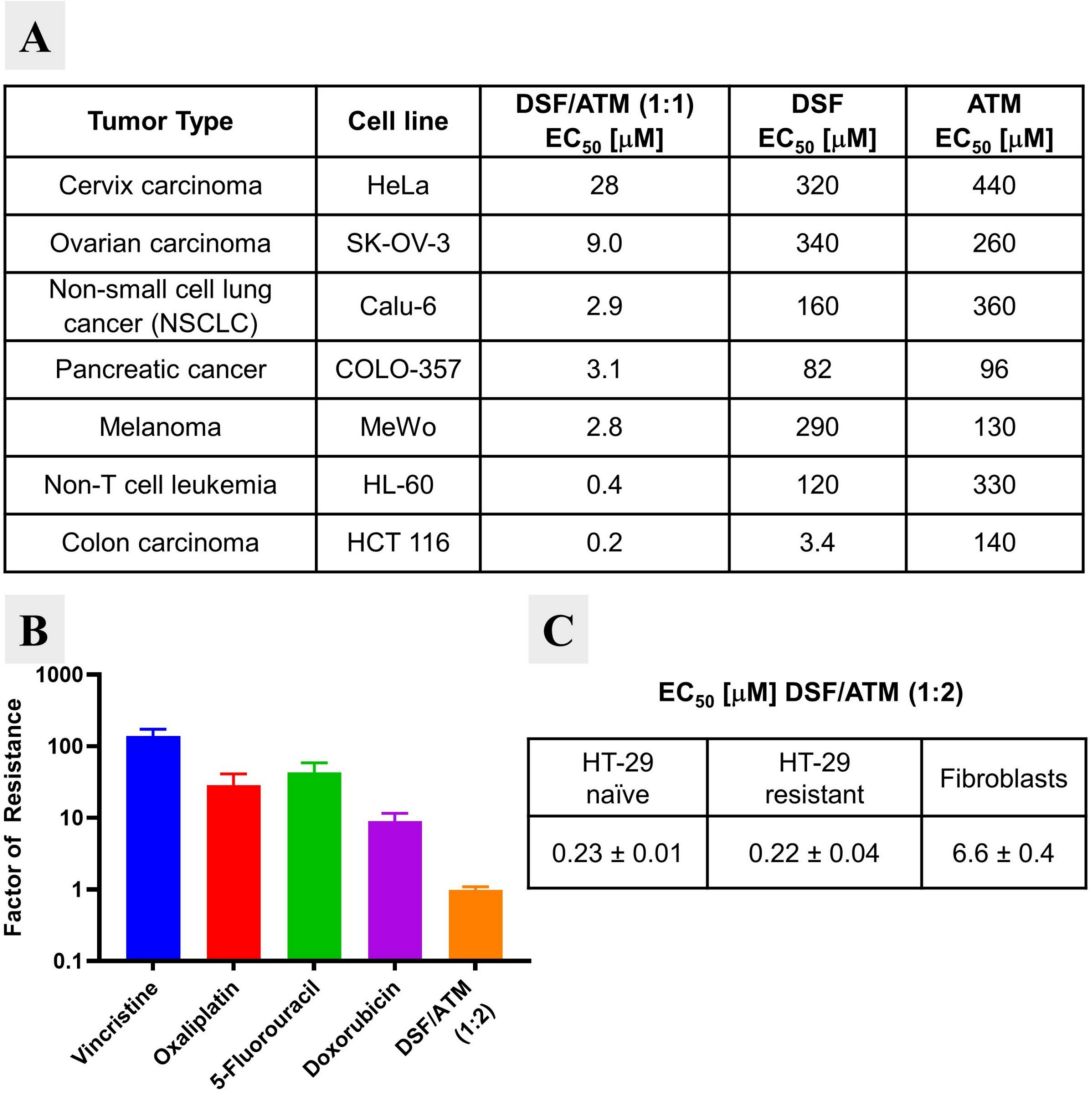
Cytotoxic effects of disulfiram (DSF) combined with aurothiomalate (ATM). **A**. EC_50_ values measured in cell lines covering seven different cancer entities for DSF, ATM, and the combination DSF/ATM (1 : 1). For all experiments, 2‐hydroxypropyl‐β‐cyclodextrin was used as formulation in 30‐fold excess. **B**. Factor of resistance measured in “therapy‐resistant” and naïve HT‐29 cells for four clinically approved drugs and the combination DSF/ATM. The HT‐29 cells were rendered “therapy‐resistant” by treating them with EC_80_ concentrations of oxaliplatin, and then re‐cultivating the surviving population. The factor of resistance is defined as the ratio of the EC_50_ values measured in “therapy‐resistant” and naïve cells, respectively. **C**. EC_50_ values for the DSF/ATM combination in naïve and “therapy‐resistant” HT‐29 cells, and human fibroblast cells. For B and C, DSF/ATM was used in a 1 : 2 ratio and 2‐hydroxypropyl‐β‐cyclodextrin was used as formulation in 30‐fold excess.

**Combination of DSF and ATM produces a cytotoxic gold complex**. In initial screening experiments, we treated cells with ATM and DSF at different ratios and always observed the formation of a precipitate, presumably the result of rapid reaction between the two compounds. As cyclodextrins are commonly used to solubilize lipophilic substances and to facilitate cellular‐uptake of small molecules, we repeated the experiment with DSF pre‐formulated in 2‐hydroxypropyl‐β‐cyclodextrin.^[7]^ In this experimental set‐up no precipitate formed and a wide range of combination ratios was cytotoxic against a panel of cell lines. For example, a 1 : 1 combination of ATM/DSF proved to be at least 10‐fold more efficient against each cell line than ATM or DSF alone (Figure 2A). Some cells were more sensitive than others to the combination treatment, with the most sensitive cell lines (HL‐60 and HCT 116) showing between one and two orders of magnitude lower EC_50_ values than the more resistant cell lines (HeLa, SK‐OV‐3, Calu‐6, COLO‐357 and MeWo). The combination of DSF and ATM also proved more effective against “therapy‐surviving” oxaliplatin‐resistant HT‐29 cells than four other standard chemotherapy agents, suggesting a potential to overcome acquired drug resistance (Figure 2B). This superior efficacy on drug‐resistant cells was not accompanied by high general toxicity as non‐transformed human fibroblasts showed 30‐fold lower sensitivity to ATM/DSF than both naïve and resistant HT‐29 cells (Figure 2C).

**Scheme 1:**
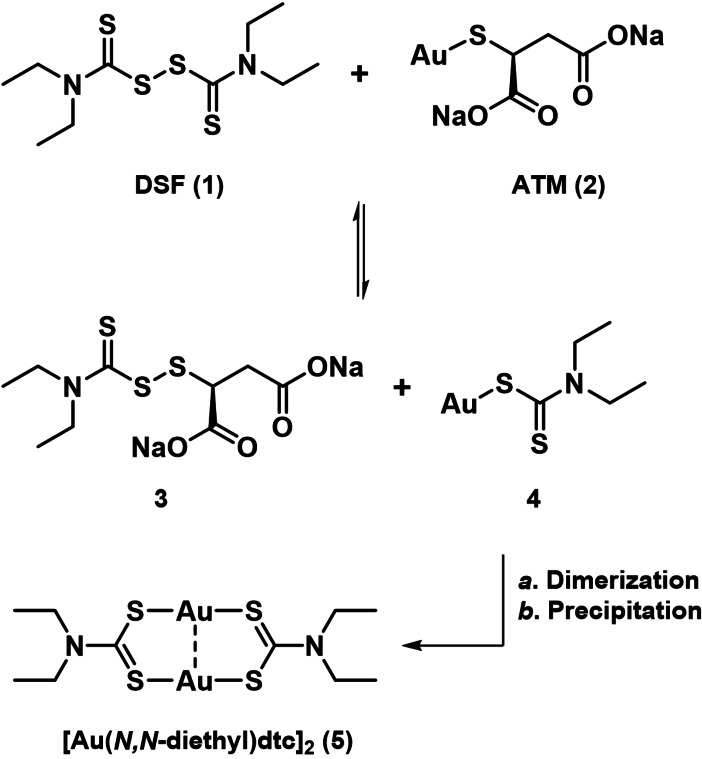
Proposed pathway of the reaction between DSF and ATM to give **5**.

Encouraged by these results, we decided to investigate the chemical outcome of the combination of DSF and ATM further. Combining the two in a 1 : 1 ratio rapidly produced an orange precipitate, which we confirmed to be the gold(I)‐dithiocarbamate complex **5**, isolated in a yield of 84 % (Scheme 1).^[8]^ In contrast to the reaction of Cu(II) salts with DSF, where a multi‐step redox process produces Cu((*N,N*‐diethyl)dtc)_2_,^[9]^ the reaction of ATM (**2**) with DSF (**1**) is redox neutral, presumably starting with a ligand exchange to give **3** and **4**. Compound **4** can stabilize itself through dimerization, forming **5**. The poor solubility of **5** shifts the equilibrium of the initial ligand exchange and serves to drive the reaction to completion. Calu‐6 cells responded to treatment with **5** formulated in 2‐hydroxypropyl‐β‐cyclodextrin (EC_50_=1.6 μM) similarly to when treated with DSF and ATM (EC_50_=2.9 μM, see Figure 2A, Figure S2), suggesting that **5** is the active cytotoxic substance.

The cytotoxic properties of gold(I)‐dtc complexes like **5** and also of gold(III)‐dtc complexes have previously been studied; however, to the best of our knowledge, no reports of selective anti‐tumoral properties of such complexes have been discussed.^[10]^ Also, the poor solubility of **5** in all common solvents, including DMSO, makes studying the cellular activity of such a compound virtually impossible in the absence of formulation. Encouraged by our initial findings, we decided to expand our initial repurposing concept and launched a medicinal chemistry campaign to prepare new analogs of **5** for two reasons: 1. Solubilization of a DSF/ATM combination or **5** requires >30 molar equivalents of cyclodextrin, a very high amount for pre‐clinical or clinical studies. We hypothesized that analogs of **5** containing different dtc moieties would require reduced amounts of cyclodextrin for solubilization. 2. We wanted to explore the role of the dtc moiety in the biological activity of such complexes. To this end, we synthesized and tested a series of dithiocarbamate complexes for solubility with the FDA‐approved formulant sulfobutylether‐β‐cyclodextrin (SBE‐β‐CD)[^7a^, ^11^] and for efficacy and selectivity in a panel of cancer cells lines. Concurrent with these biological studies, we became interested in the structural aspects of these gold complexes and their interaction with cyclodextrins in general.

**Figure 3:**
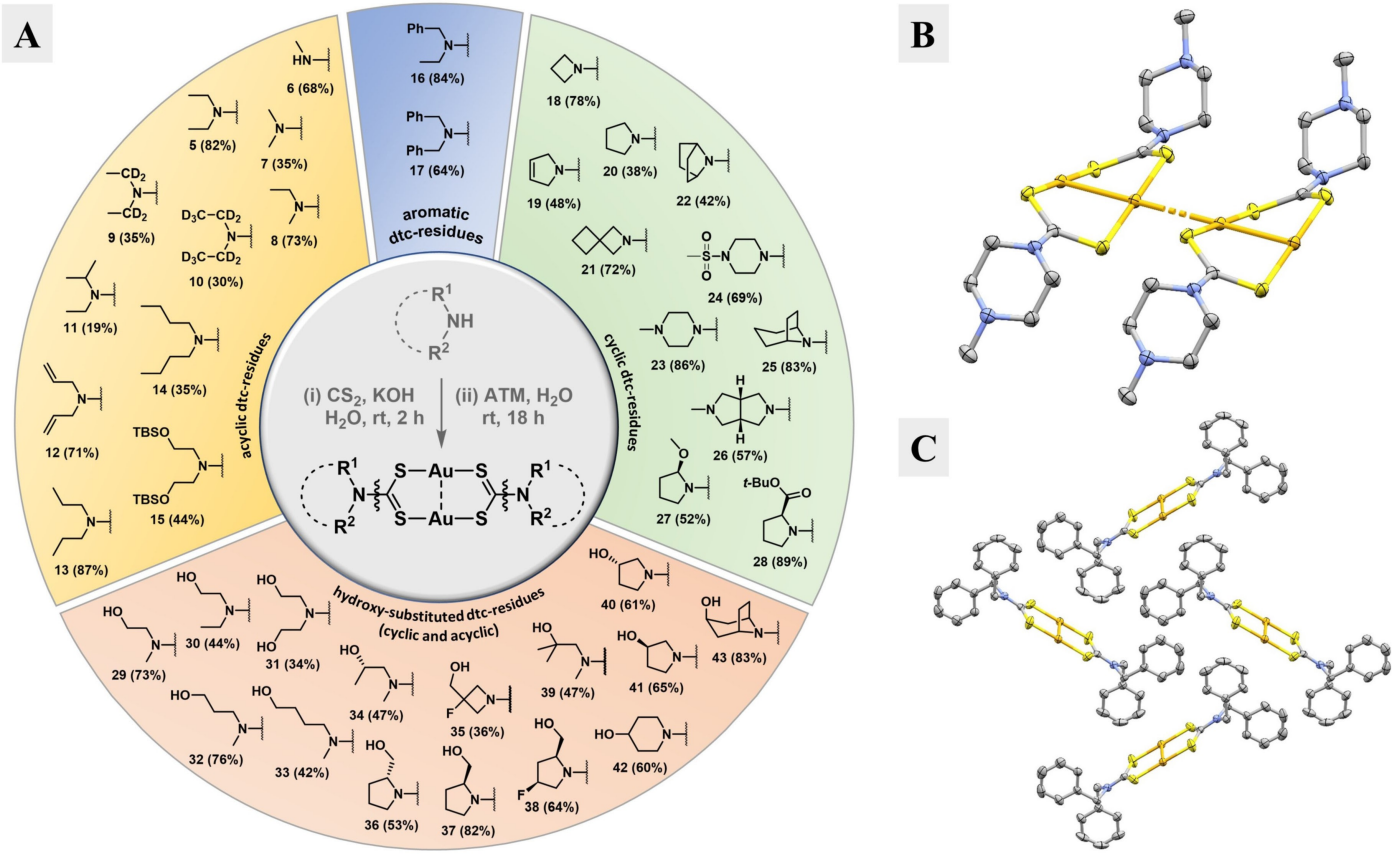
Synthesis and experimental structures of dimeric gold(I)‐dtc complexes. **A**. Synthesized gold(I)‐dtc complexes with dithiocarbamates containing acyclic (orange), aromatic (blue), cyclic (green) and hydroxy‐substituted (red) residues. Isolated yields are shown in parenthesis; also shown is the primarily used synthetic route (grey middle circle). **B**. Experimental structure of **23** highlighting the co‐linear alignment of the gold atoms due to aurophilic bonding. **C**. Experimental structure of **17**; view of the asymmetric unit along the crystallographic a‐axis. For B and C: Ellipsoids are shown at a 50 % probability level. Hydrogens are omitted for clarity. Color code: carbon, grey; nitrogen, blue; sulfur, yellow; gold, orange.^[17]^

**Structural aspects of gold(I)‐dithiocarbamate complexes**. Numerous reports deal with the synthesis and characterization of dimeric gold(I)‐dtc complexes such as **5**. These molecules can have R^1^ and R^2^ groups that are identical[^8b^, ^12^] or different from each other,[^10a^, ^13^] either as distinct substituents or connected in a cyclic system (Figure 3A, central circle).[^12c^, ^14^] Most of the known examples are highly symmetric complexes with identical R^1^ and R^2^ groups. These complexes are typically synthesized by mixing an alkali dithiocarbamate salt and a source of Au(I), such as (Ph_3_P)AuCl^[8b]^ or (Me_2_S)AuCl,[^13^, ^14^] or *in situ* reduced Au(III) salts.[^10a^, ^12a^] We found using ATM as a source of Au(I) to be superior. This approach takes advantage of the poor water solubility of most dimeric gold(I)‐dtc complexes and the excellent water solubility of dithiocarbamate salts and ATM. As such, a lithium or potassium dithiocarbamate salt, prepared from an amine and CS_2_, either in a separate step or *in situ*, is added to an aqueous solution of ATM. The resulting gold(I)‐dtc complexes precipitate from the solution and can be isolated in analytically pure form by filtration and washing with water. This convenient method provided a variety of functionalized complexes in moderate to excellent yields and high purity. We categorized the complexes into four different groups based on their dtc substituents: acyclic (orange), aromatic (blue), cyclic (green) and hydroxy‐substituted (red) (Figure 3A).^[15]^

Many of the synthesized complexes are poorly soluble in organic solvents at ambient temperature. Some derivatives could be solubilized at high temperatures and, upon cooling, crystallized. In this manner, x‐ray quality crystals of **23** (Figure 3B) and **17** (Figure 3C) were obtained, complementing the set of available experimental structures (Table S1). Three interesting structural features of gold(I)‐dtc complexes are exemplified when comparing these two structures. First, multiple molecules of piperazine derivative **23** stack their gold atoms to form linear “wires” through the crystal lattice with a distance of 3.24 Å to adjacent dimeric gold complexes (Figure 3B). Such “aurophilic bonding” is frequently found in the solid state of closed‐shell Au(I) (5d^10^) complexes (and gold(I)‐dtc complexes in general, see Table S1).^[16]^ In contrast, the gold atoms in benzyl derivative **17** make no intermolecular contacts (Figure 3C). This situation is rarely observed for gold(I)‐dtc complexes – in a thorough CCDC search, we found ten similar gold(I) complexes with different dtc moieties, of which only two showed no aurophilic bonding. In these two specific cases the steric bulk of the dtc moiety or co‐crystallized compounds prevent this aggregation (see Table S1). In the case of **17** both situations do not apply. Presumably, favorable hydrophobic interactions of the benzyl groups energetically outweigh stabilization via intermolecular aurophilic contacts.

**Figure 4:**
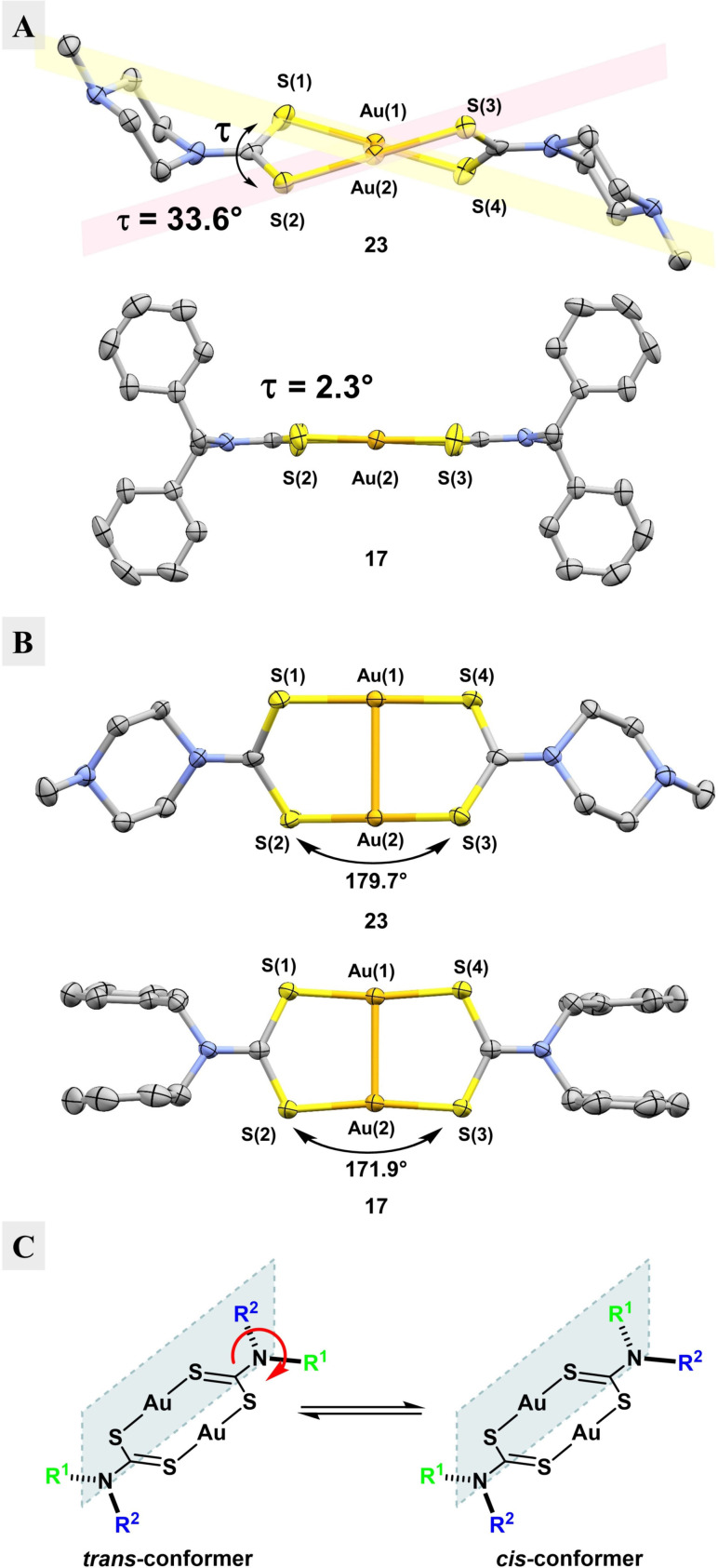
Structural aspects of gold(I)‐dtc complexes. **A**. Depiction of the twist angle τ, which can be calculated by taking the mean of both dihedral angles S(1)−Au(1)−Au(2)−S(2) and S(3)−Au(2)−Au(1)−S(4), for **17** and **23. B**. Depiction of the S−Au−S angles for **17** and **23. C**. Depiction of the possible *cis*/*trans*‐conformers in gold(I)‐dtc complexes bearing different R^1^ and R^2^ groups.^[17]^

Second, the eight‐membered rings defined by the two gold atoms and the two S−C−S fragments of the dithiocarbamates have quite different conformations in the two structures, indicating flexibility in the ring. This can best be described by the twist angle τ, which we define to be the angle, when viewed down the Au−Au axis, formed by lines made by connecting S1 with S4 and S2 with S3 (Figure 4A). While piperazine derivative **23** has a twisted eight‐membered ring with τ=33.6°, benzyl derivative **17** has an almost flat eight‐membered ring with τ=2.3°.^[18]^ Another distinguishing feature of the eight‐membered ring is the angle of the S−Au−S bonds: in compound **23**, this is nearly linear with a mean angle of 179.7°, but it significantly deviates from linearity at 171.9° for complex **17**, presumably due to an intramolecular aurophilic interaction between the two gold atoms (Figure 4B). Lastly, the R^1^R^2^N−CS_2_ bond shows considerable double bond character. This is apparent from the R−N−C−S torsion angle being close to 0°, and also from the IR spectrum of **5** (Figure S1) that shows absorption peaks at around 1500 cm^−1^. These can be attributed to the “thioureide‐band”, which is between a carbon‐nitrogen single bond at $\tilde{v}$ =1250–1350 cm^−1^ and a double bond at $\tilde{v}$ =1640–1690 cm^−1^.^[10a]^ A consequence of this amide‐like double bond character are “*cis*/*trans*‐conformers” that, due to restricted bond rotation, constitute energetic minima for compounds where R^1^ and R^2^ are different (Figure 4C). In the case of **23**, R^1^ and R^2^ are identical, but rotation around the thioureide C−N bond still produces two distinct conformations, because the cyclic piperazine moiety adopts a chair‐conformation with the *N*‐methyl group in a favorable equatorial position. One conformation orients both piperazines “above” the eight‐membered ring (not observed), whereas the other (observed) displays the piperazines on opposite sides in an *anti*‐conformation, as shown in Figure 4A.

**Figure 5:**
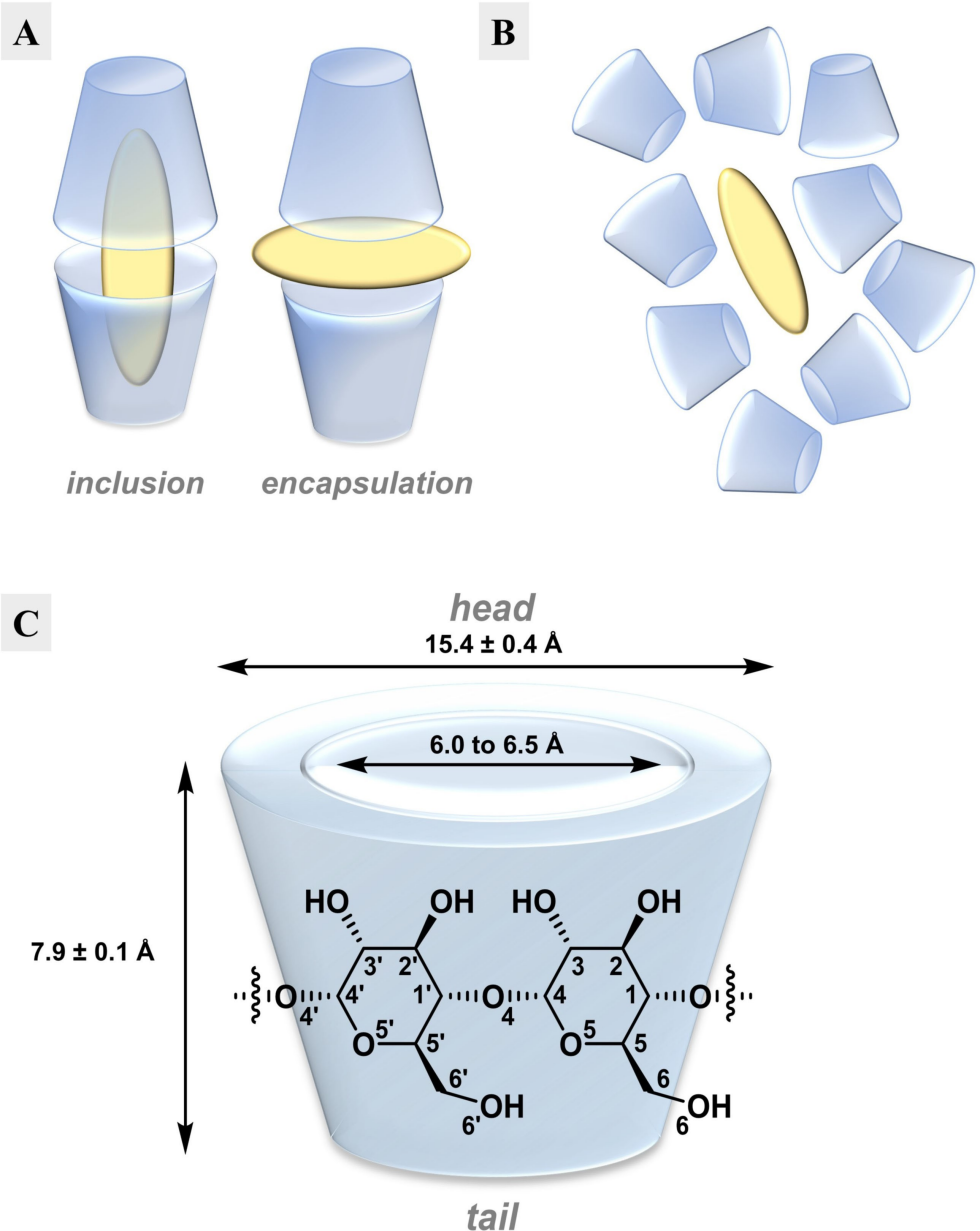
β‐cyclodextrin as a host for host–guest complexes. **A**. Sketch visualizing different common modes of interaction of β‐CD with a lipophilic compound with a 2 : 1 host (blue)/guest (yellow) ratio – formation of an inclusion complex with a “head‐to‐head”‐arrangement of the CD cones and encapsulation of the guest by β‐CD in the interstitial space. **B**. Proposed less‐ordered aggregates formed with insoluble compounds like **5** (yellow) in the presence of high excess of β‐CD (blue) as a special case of encapsulation. **C**. Schematic depiction of β‐cyclodextrin.

**Dimeric gold(I)‐dtc complexes form inclusion complexes with β‐Cyclodextrin**. We noticed that the hydroxy‐substituted complexes are, in general, more soluble than the other classes, with many (**29**–**39**) being readily soluble in some organic solvents such as DMSO (see Figure 3A). More importantly, less SBE‐β‐CD was required to prepare standard aqueous solutions of these complexes. To better quantify the amount of SBE‐β‐CD that was needed for each gold(I)‐dtc complex, we performed a modified kinetic solubility experiment on a selection of compounds (Figure S3). While there is no apparent solubility trend of the complexes with respect to the size or constitution of the dithiocarbamate side chains, we were intrigued that some complexes only require cyclodextrin ratios as low as 2 to 3 (for **34** and **32**, respectively) for solubilization. These relatively low ratios suggested to us that such compounds might be capable of forming distinct 2 : 1 host–guest inclusion or encapsulation complexes with cyclodextrins (Figure 5A),^[19]^ as opposed to less‐ordered aggregates (Figure 5B), which might be more operative under high cyclodextrin concentrations as required for **5**.

To explore this hypothesis, we attempted to grow crystals of selected gold complexes out of cyclodextrin solutions. Because SBE‐β‐CD is not a homogenous material, we used solutions of β‐cyclodextrin (Figure 5C)^[20]^ that were saturated with specific gold complexes, and allowed crystals to form via slow evaporation of water. From these experiments, we were able to isolate crystals from seven different gold complexes (**30**, **32**, **33**, **35**, **37**, **39**, and **43**). NMR analysis of crystals re‐dissolved in DMSO‐*d_6_* revealed all had 2 : 1 host/guest ratios (Figure S4).^[21]^ For the crystals grown from solutions of **33**, **39** and **43**, we were able to obtain well‐resolved X‐ray structures, thereby confirming the formation of inclusion complexes.^[22]^

**Figure 6:**
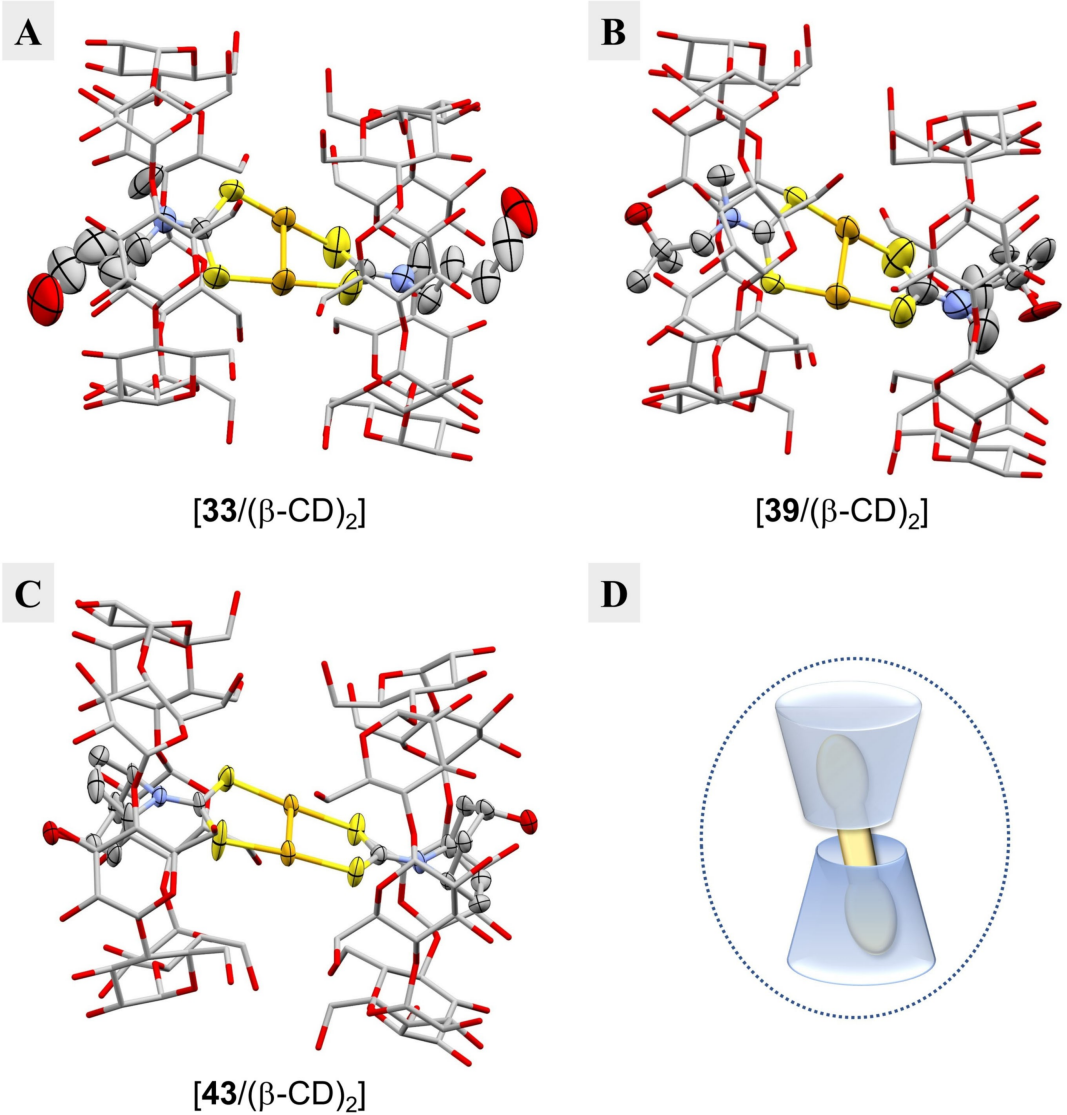
Experimental structures of the inclusion complexes of **33**, **39** and **43** with β‐CD. **A**. Depiction of [**33**/(β‐CD)_2_]; shown is the major component (site occupancy factor (s. o. f.) 71 %). **B**. Depiction of [**39**/(β‐CD)_2_]; shown is the major component (s. o. f. 52 %). **C**. Depiction of [**43**/(β‐CD)_2_]. **D**. Sketch visualizing the obtained inclusion complexes of gold(I)‐dtc complexes (yellow) with β‐cyclodextrin (blue) in a 2 : 1 host/guest ratio and a “tail‐to‐tail” arrangement of the CD cones. For A–C: Hydrogens and water molecules are omitted for clarity and the β‐cyclodextrin cones are shown as capped‐stick model whereas **33**, **39** and **43** are depicted as ellipsoids (on a 50 % probability level). Color code: carbon, grey; oxygen, red; nitrogen, blue; sulfur, yellow; gold, orange.^[17]^

The experimental structures were elucidated in resolutions of 0.91 Å (**33**), 0.91 Å (**39**) and 0.84 Å (**43**), and all three inclusion complexes share common features (Figure 6A–C). The gold(I)‐dtc complexes are included into two β‐CDs that arrange in a “tail‐to‐tail” manner (2 : 1 host/guest ratio) (Figure 6D). This counterintuitive arrangement of the CD cones is only rarely observed in inclusion complexes.^[23]^ The organic residues of each gold(I)‐dtc complex reach out of the wide “head”‐side of the corresponding cyclodextrin, whereas the gold atoms of the eight‐membered ring system are located within the cleft of the cyclodextrin cones.

The primary hydroxy groups of the narrow “tail” side of each CD cone are all in a (−)‐gauche orientation, as in uncomplexed β‐cyclodextrin (*i. e*. pointing towards the outside of the cavity; Figure 5C) indicating no direct interaction with the complex on the inside. Both β‐CD cones of the inclusion complexes are arranged almost parallel to each other – the angle between the mean plane through the seven glycosidic oxygens of both cyclodextrins is 1.1° for [**33**/(β‐CD)_2_], 5.7° for [**39**/(β‐CD)_2_] and 3.7° for [**43**/(β‐CD)_2_] – and are not precisely stacked on top of each other but are shifted sideways by approximately 2.2 to 2.4 Å (see Figure S5 for a detailed description of the determination of the structural parameters).

In complex **33** (Figure 6A) the gold atoms are disordered (three‐fold disorder of the eight‐membered ring system; not shown). As a consequence, one of the carbamate moieties is fixed in its position within the cavity of the host, whereas the other one adopts different positions due to different twist angles of the eight‐membered ring system (twist angle τ=33.1° for the major component; site occupancy factor (s. o. f.) 71 %). In contrast, the eight‐membered ring system of **39** (Figure 6B) shows no disorder. It has a twist angle τ=29.1°, and the gold‐complex exists as a nearly 1 : 1 mixture of *cis*/*trans*‐conformers (s. o. f. 52 % for the major component). Complex **43** (Figure 6C) also shows no disorder and is solely present in an *anti*‐conformation similar to **23** (Figure 3B). It has a twist angle τ=9.9°, resulting in the most planar eight‐membered ring system of the three gold(I)‐dtc inclusion complexes.

**Figure 7:**
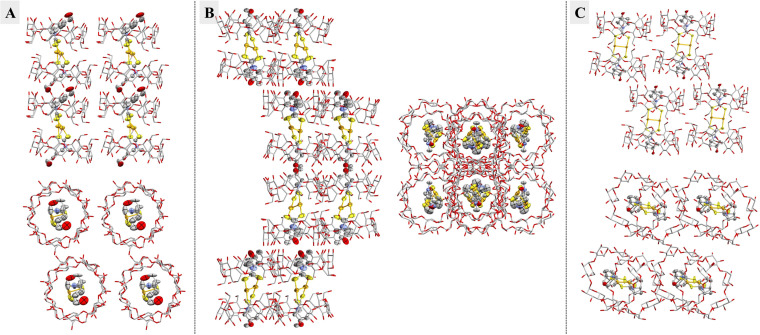
Lateral and axial views of the inclusion complexes. **A**. Depiction of [**33**/(β‐CD)_2_]; shown is the major component (s. o. f. 71 %). **B**. Depiction of [**39**/(β‐CD)_2_]; shown is the major component (s. o. f. 52 %). **C**. Depiction of [**43**/(β‐CD)_2_]. For A–C: Hydrogens and water molecules are omitted for clarity and the β‐cyclodextrin cones are shown as capped‐stick model whereas **33**, **39** and **43** are shown as ellipsoids (on a 50 % probability level). Color code: carbon, grey; oxygen, red; nitrogen, blue; sulfur, yellow; gold, orange.^[17]^

Interestingly, the stacking of the inclusion complexes in the crystal lattice is different for all obtained experimental structures. In [**33**/(β‐CD)_2_] the CD cones of the inclusion complexes form channel‐like structures, stacking up to make “infinite” pipes (Figure 7A). In [**39**/(β‐CD)_2_] the CD cones are staggered in pairs with only every second unit stacking‐up to one adjacent unit (Figure 7B). In [**43**/(β‐CD)_2_] the inclusion complexes are arranged in a brick‐like structure (Figure 7C). In each case the interstitial space of the CD‐cones as well as between the inclusion complexes is filled with water molecules stabilizing this assembly by an extensive network of hydrogen bonds (not shown). No water molecules were found within the cavity of the β‐cyclodextrins, indicating that they are completely filled by the organic ligands of the gold complexes.

**Figure 8:**
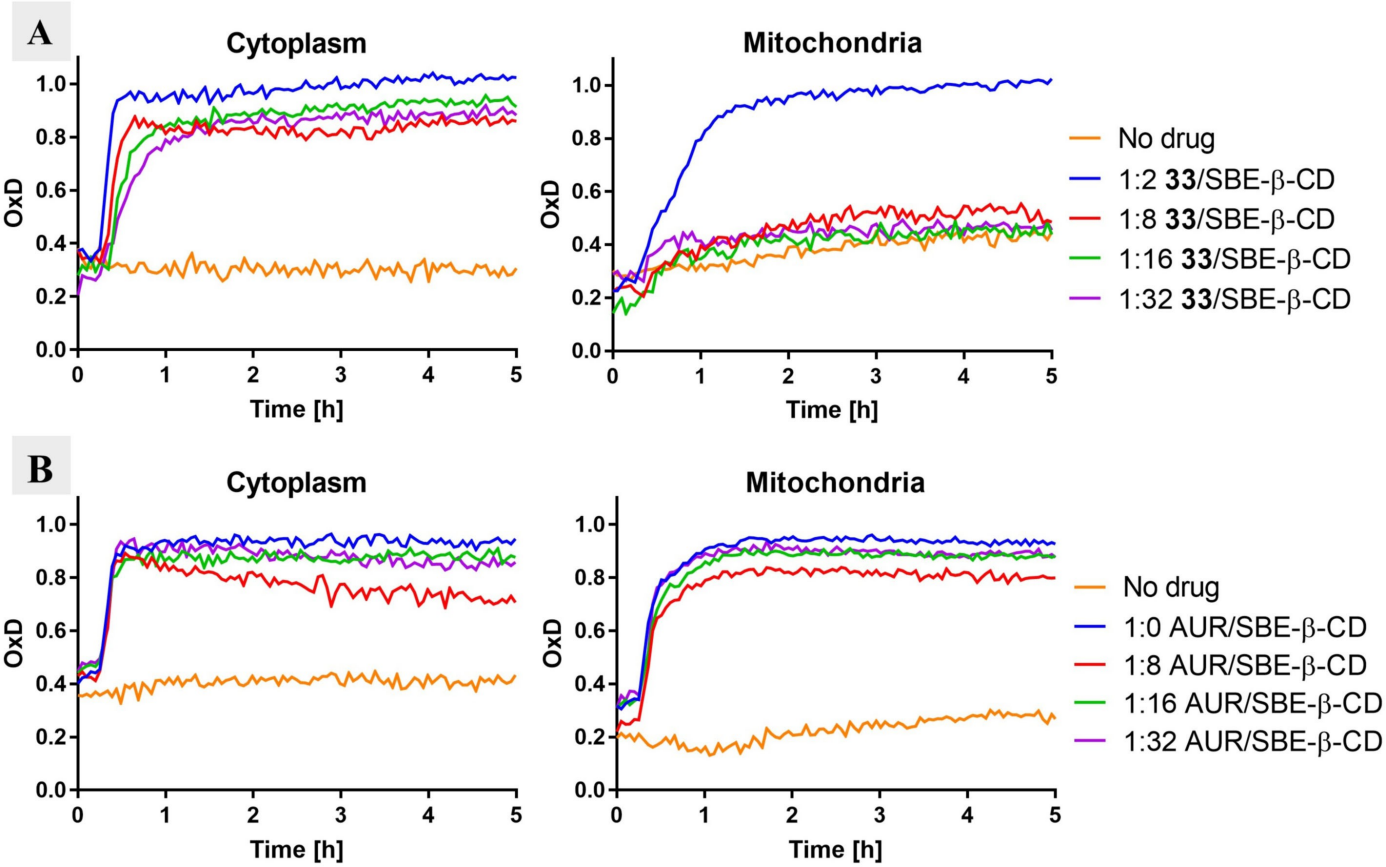
Measurement of ROS levels in the cytoplasm and mitochondria using the roGFP2‐Orp1 sensor. **A**. Kinetic data after treatment of H838 cells with **33** (12.5 μM) formulated with different amounts of SBE‐β‐CD. **B**. Kinetic data after treatment of H838 cells with auranofin (AUR) (12.5 μM) either from a DMSO stock solution or formulated with different amounts of SBE‐β‐CD. For A and B: “No drug” is a control experiment with only vehicle to illustrate the background oxidation state of the sensor. OxD is the degree of probe oxidation with the data normalized to 1.0 (fully oxidized) defined by the signal from diamide (2 mM) and 0.0 (fully reduced) defined by the signal from DTT (10 mM). Data is presented as the mean of 3 technical replicates.

**SBE‐β‐CD reduces mitochondrial ROS formation from dimeric gold(I)‐dtc complexes**. Many cancer cells are sensitive to elevated reactive oxygen species (ROS) levels, and metal complexes are known to increase ROS by disrupting proteins that regulate cellular redox homeostasis. Normal cells are not immune to ROS‐induced damage, and bursts of ROS in mitochondria can cause cardiac toxicity. This is particularly problematic for the safe use of metallodrugs in animals and patients.^[24]^ We therefore measured the effects of our gold(I)‐dtc complexes on cellular ROS levels using a genetically encoded ROS‐sensor (roGFP2‐Orp1), which can be selectively localized to either the cytoplasm or the mitochondria.^[25]^ Surprisingly, many of our compounds produced a rapid and persistent increase in cytoplasmic ROS while having little effect on mitochondrial ROS (data not shown). In order to discern the source of this effect, we measured ROS‐induction by **33** in the moderately sensitive non‐small cell lung cancer H838 cell line in the presence of different amounts of SBE‐β‐CD. Formulations of **33**/SBE‐β‐CD in ratios of 1 : 32, 1 : 16, 1 : 8, and 1 : 2 produced high cytoplasmic ROS at similar levels (Figure 8A). In contrast, only a 1 : 2 **33**/SBE‐β‐CD formulation generated high ROS in mitochondria, while all other ratios showed no difference to a control sample. This suggests that SBE‐β‐CD is not a passive solubilizing agent in these experiments, but that it provides a “mito‐protective” effect. Interestingly, this effect appears not to be universal: in an identical experimental setup, auranofin produced high levels of ROS in both the cytoplasm and mitochondria up to a 32‐fold excess of SBE‐β‐CD (Figure 8B).

To the best of our knowledge, a mito‐protective role of cyclodextrins has not been described before. Cyclodextrins in general don't cross cell membranes, and SBE‐β‐CD, which has on average seven sulfonates per CD molecule, should also have no passive cell permeability.[^25^, ^26^] The possibility that formulation in a cell‐impermeable host can selectively impact a sub‐cellular drug phenotype is enticing, but the exact mechanism awaits elucidation. The effect may result from gold(I)‐dtc/SBE‐β‐CD inclusion complexes, which then form higher‐order oligomers consisting of drug‐loaded and free SBE‐β‐CD molecules. Such oligomeric structures have been previously described,^[27]^ and if active transport across the membrane (*e. g*. endocytosis) was possible, an explanation for the effect could be envisaged. The fact that auranofin produces mitochondrial ROS in the presence of excess SBE‐β‐CD suggests that formation of an inclusion complex, which is unlikely for auranofin due to its size, is required for mito‐protection. It cannot be excluded, however, that auranofin induces mitochondrial ROS via a different mechanism of action than gold(I)‐dtc complexes. Studies to understand this effect in greater detail are currently underway in our laboratory, and will be reported along with pre‐clinical studies of gold(I)‐dtc complexes as anti‐cancer agents.

## Conclusion

We found that the two drugs disulfiram and aurothiomalate, when combined in the presence of β‐cyclodextrins, are selectively cytotoxic against a collection of cancer cell lines and show no cross‐resistance in therapy‐surviving HT‐29 cells. The two drugs react to make the dimeric gold(I)‐dtc complex **5**, which we showed to be the active component, and subsequently prepared >35 analogs for further study. Crystallographic analysis of selected dimeric gold(I)‐dtc complexes alone, or as inclusion complexes with β‐cyclodextrin, revealed a diversity of conformations for the central eight‐membered scaffold, as well as interesting stereochemical considerations with respect to cyclic and non‐symmetric dtc ligands. Finally, we discovered that when formulated with an excess of SBE‐β‐CD, the gold(I)‐dtc complex **33** does not induce mitochondrial ROS. The same formulation of the related gold(I) complex auranofin provides no protection against mitochondrial ROS, suggesting that SBE‐β‐CD has untapped potential beyond just a passive solubilizing agent.

## Experimental Section

### Chemistry

**General**: Reactants and reagents were obtained from Sigma‐Aldrich (Germany), Alfa Aesar (Germany), Fisher Scientific (Germany), Carl Roth (Germany) and Enamine (Ukraine), and were used without further purification. Deionized water was used from in‐house supply. Microwave reactions were done using a Biotage Initiator^+^ Microwave Synthesizer. NMR spectra were recorded on a Bruker 400 MHz instrument (DKFZ) and on a Bruker Avance III 600 MHz instrument (Inorganic Chemistry Department, University of Heidelberg). High resolution mass spectrometry was recorded on a Bruker ApexQe FT‐ICR instrument (Department of Organic Chemistry, University of Heidelberg). Elemental analyses were performed on a vario Micro Cube by the “Microanalysis Laboratory” (Department of Organic Chemistry, University of Heidelberg). X‐ray crystallography was either performed on a Bruker APEX II Quazar (with Mo–Microsource) or a Stoe Stadivari (with Cu–Microsource and Pilatus‐detector) instrument (Department of Organic Chemistry, University of Heidelberg).

**Synthesis of 5 via ATM and DSF**: A solution of ATM (195.0 mg, 0.5 mmol, 1.0 equiv.) in water (10.0 mL) and a solution of DSF (148.3 mg, 0.5 mmol, 1.0 equiv.) in EtOH (10.0 mL) were mixed and vigorously stirred at room temperature for 4 h. The resulting precipitate was filtered using a glass frit (pore 4), thoroughly washed with water (200 mL) and afterwards dried under high vacuum overnight. The pure product was obtained as an orange‐colored powder in a yield of 84 % (145.6 mg, 0.21 mmol). MALDI‐MS (*m/z*) [Au^III^+2 dtc^−^]^+^ calcd. for C_10_H_20_AuN_2_S_4_: 493.018; Found: 493.026. Anal. calcd for C_10_H_20_Au_2_N_2_S_4_: C, 17.39; H, 2.92; N, 4.06; Found: C, 17.69; H, 3.20; N, 3.83. The precipitate was recrystallized from hot DMF to afford orange‐colored needles. MALDI‐MS (*m/z*) [Au^III^+2 dtc^−^]^+^ calcd. for C_10_H_20_AuN_2_S_4_: 493.018; Found: 493.018. Anal. calcd for C_10_H_20_Au_2_N_2_S_4_: C, 17.39; H, 2.92; N, 4.06; Found: C, 17.54; H, 3.14; N, 3.74. The IR‐spectra of both the initially obtained and recrystallized material are identical (Figure S1).

**General procedure for the synthesis of gold(I)‐dtc complexes**: To a solution of the appropriate amine (0.92 mmol, 1.2 equiv.) and potassium hydroxide (0.92 mmol, 1.2 equiv.; for amine hydrochlorides 2.4 equiv. of KOH was used) in water (10.0 mL) was added carbon disulfide (0.92 mmol, 1.2 equiv.). The solution was stirred at room temperature for 2 h in order to assure complete formation of the corresponding dithiocarbamate (dtc). Then a solution of sodium aurothiomalate (ATM) (0.77 mmol, 1.0 equiv.) in water (15.0 mL) was added to the solution. A yellow to orange‐colored precipitate formed immediately. The suspension was stirred at room temperature overnight, filtered through a glass frit (pore 4), and thoroughly washed with distilled water (2×20 mL). This material was dried under high vacuum overnight (and, if necessary, recrystallized from an appropriate solvent) to afford the pure product. In most cases, due to the poor solubility of the gold(I)‐dtc complexes, analytics are based on elemental analysis and high‐resolution mass spectrometry (ESI). Concerning HRMS, a diagnostic fragmentation ion (*i. e*. [Au^III^+2 dtc^−^]^+^) specific for these gold(I)‐dtc complexes was regularly found.

**Crystallization of inclusion complexes**: A mixture of the gold(I)‐dtc complexes (0.05 mmol) and β‐cyclodextrin (0.05 mmol) in water (5.0 mL) (HPLC grade), resulting in a 10 mM mixture of the components in a 1 : 1 host/guest ratio, was vigorously stirred at 50 °C for 3 h. Then the mixtures/suspensions were taken up in a 10 mL syringe and filtered through a syringe filter (Millex®‐HV, 0.45 μm, Merck‐Millipore) into a 20 mL glass vial. Those vials were left for slow evaporation in a ventilated fume hood for several days. When initial crystal formation was detected, the vials were capped and transferred to a 4 °C refrigerator for several days (and left there until the actual X‐ray measurements). For the NMR experiments of these inclusion complexes, the crystalline material was filtered through a round filter paper and washed with a minimal amount of water.

### Biology

**Quantification of drug sensitivity (**Figure 2A**)**: Cell lines (including HT‐29) were obtained from ATCC or as gifts from other research groups at the DKFZ and checked for mycoplasma contamination and authenticity before use. Cell lines were seeded in 96‐well plates in RPMI‐1640 medium, supplemented with 10 % BCS (bovine calf serum) and 1 % Pen/Strep at a density of 2×10^4^ cells/well. Disulfiram (DSF) and sodium aurothiomalate (ATM) were added at a fixed ratio of 1 : 1, formulated in a 30‐fold molar excess of 2HP‐β‐CD (2‐hydroxypropyl‐β‐cyclodextrin). ATM and DSF monotherapy was also done in the presence of 2HP‐β‐CD, to control for possible combination effects by 2HP‐β‐CD. All concentrations were applied in triplicate. After incubation at 37 °C for 72 h, CellTiter‐Blue (Promega) was added (10 μL/well). After incubation at 37 °C for 4 h, fluorescence (excitation filter: 550 nm, emission filter 590 nm) was determined in an Optima FLUOstar microplate reader (BMG Labtech). Mean values were calculated for each treatment and EC_50_ values were calculated from non‐fitted dose response curves.

**Quantification of factor of resistance (**Figure 2B and 2C): 10 million HT‐29 human colon cancer cells were seeded in 175 mL Falcon flasks. After 24 h, 10 μM oxaliplatin was added. After 48 h incubation at 37 °C, the medium was replaced with fresh medium. After 48 h, cells were trypsinized and seeded in 96‐well plates at a density of 2 x 10^4^ cells per well. Human skin fibroblasts (obtained from the Darai lab, Institute of Virology, University of Heidelberg) and untreated HT‐29 cells were seeded in 96‐well plates at the same density. After 24 h incubation at 37 °C, cells were treated in triplicates with 1 : 2 serial dilutions of vincristine sulfate (starting concentration 1 μg/mL; 1.1 μM), oxaliplatin (starting concentration 50 μg/mL; 126 μM), 5‐fluorouracil (5‐FU) (starting concentration 500 μM), doxorubicin (starting concentration 10 μg/mL; 17.2 μM) and DSF/ATM (molar ratio of 1 : 2), formulated in 30‐fold molar excess of 2HP‐β‐CD (starting concentration 2 mM ATM). After incubation for 72 h at 37 °C, 10 μL of CellTiter‐Blue was added per well. After incubation at 37 °C for 4 h, fluorescence (excitation filter: 550 nm, emission filter 590 nm) was determined in an Optima FLUOstar microplate reader (BMG Labtech). Mean values were calculated for each treatment and EC_50_ values were calculated from non‐fitted dose response curves. Calculation of “Factor of resistance”: For each treatment EC_50_ values in oxaliplatin treatment surviving cells were divided by the EC_50_ values found with the same treatment in unselected HT‐29 cells. A factor of resistance >1 indicates “resistance”. A factor of resistance=1 indicates “no resistance”.

**Measurement of ROS levels in cytoplasm and mitochondria** (Figure 8): The effects of **33** and auranofin on roGFP2‐Orp1 oxidation were quantified in H838 cells stably expressing roGFP2‐Orp1 targeted either to cytoplasm or mitochondria as described in Ezerina *et* *al*.^[28]^ Specifically, a 5 mM stock solution of **33** was prepared by directly dissolving solid **33** into 10 mM, 40 mM, 80 mM, and 160 mM solutions of SBE‐β‐CD (Biomol) in PBS (Gibco). A 100 mM auranofin (Sigma‐Aldrich) solution was prepared in DMSO (Sigma‐Aldrich). This was further diluted to 0.5 mM stock solutions of auranofin in 0 mM, 4 mM, 8 mM, and 16 mM SBE‐β‐CD in PBS. Cells were seeded into a black clear‐bottomed 96‐well imaging plate (Corning) at a density of 10,000 cells/well in 100 μL Fluorobrite medium (Gibco) 16 to 18 h before the measurement. Non‐transduced H838 cells were seeded on the same plate to establish background fluorescence. The fluorescence intensity from these wells was subtracted from all the measurement results. To establish the signal baseline, the entire plate was measured every 3 min for 8 cycles in a CLARIOstar fluorescence plate reader (BMG Labtech), with excitation wavelengths of 400 nm and 485 nm, while measuring emission at 520 nm. Next, 100 μL of 2 times concentrated drug was added, and measurement was continued for up to 100 cycles. 2 mM diamide (3‐(dimethylcarbamoylimino)‐1,1‐dimethylurea) and 10 mM DTT (dithiothreitol) were used as positive and negative controls, respectively, for probe oxidation. Fluorobrite medium was used as a non‐treated control. The readout of the roGFP2 measurement is normalized to the positive (1.0) and the negative (0) controls using the following equation:

$${OxD}_{roGFP2}=\frac{{(I400}_{sample})({I485}_{red})-\left({I400}_{red})({I485}_{sample}\right)}{{(I400}_{sample})\left({I485}_{red}-{I485}_{ox}\right)+\left({I485}_{sample})({I400}_{ox}-{I400}_{red}\right)}$$

where I400 and I485 are the signal intensities measured at 520 nm after excitation at 400 nm and 485 nm, respectively. The subscripts “ox” and “red” indicate the signal intensity of fully oxidized and fully reduced controls, respectively. All treatments were performed in technical triplicate.

## Author Contributions

M. M. synthesized compounds and analyzed inclusion complexes. P. F. and E. A. performed biological experiments. N. G. supervised biological experiments. A. K. M. supervised chemical experiments. M. M. and A. K. M. wrote the manuscript. All authors have given approval to the final version of the manuscript.

## Associated Content

The Supporting Information of this article contains: Experimental details for the synthesis of each compound and corresponding NMR spectra, details of the kinetic solubility and X‐ray crystallographic measurements, depiction of the experimental structure of [**37**/(β‐CD)_2_], the IR spectrum of **5**, NMR spectra of the inclusion complexes, a detailed description of the determination of structural parameters of the inclusion complexes as well as a list of all gold(I)‐dtc complexes deposited at Cambridge Crystallographic Data Centre (CCDC). 2068758 (for **17**), CCDC 2068757 (for **23**), CCDC 2068754 (for [**33**/(β‐CD)_2_]), CCDC 2068759 (for [**37**/(β‐CD)_2_]), CCDC 2068755 (for [**39**/(β‐CD)_2_]) and CCDC 2068756 (for [**43**/(β‐CD)_2_]) contain(s) the supplementary crystallographic data for this paper. These data are provided free of charge by the joint Cambridge Crystallographic Data Centre and Fachinformationszentrum Karlsruhe Access Structures service www.ccdc.cam.ac.uk/structures.

## Abbreviations

2‐HP‐β‐CD, 2‐hydroxypropyl‐β‐cyclodextrin; ATM, sodium aurothiomalate; CD, cyclodextrin; dtc, dithiocarbamate; DSF, disulfiram; s. o. f., site occupancy factor; DTT, dithiothreitol.

## Conflict of interest

The authors declare no competing financial interests.

## Supporting information

As a service to our authors and readers, this journal provides supporting information supplied by the authors. Such materials are peer reviewed and may be re‐organized for online delivery, but are not copy‐edited or typeset. Technical support issues arising from supporting information (other than missing files) should be addressed to the authors.

Supporting Information

## References

[1a] F. S. Collins, Nat. Rev. Drug Discov. 2011, 10, 397; 21629277 10.1038/nrd3461 PMC5101935

[1b] Z. Zhang, L. Zhou, N. Xie, E. C. Nice, T. Zhang, Y. Cui, C. Huang, Sig. Transduct. Target. Ther. 2020, 5, 113. 10.1038/s41392-020-00213-8 PMC7331117 32616710

[2] E. Ekinci, S. Rohondia, R. Khan, Q. P. Dou, Recent Pat. Anti-Cancer Drug Discov. 2019, 14, 113–132. 10.2174/1574892814666190514104035 31084595

[3] Z. Skrott, M. Mistrik, K. K. Andersen, S. Friis, D. Majera, J. Gursky, T. Ozdian, J. Bartkova, Z. Turi, P. Moudry, M. Kraus, M. Michalova, J. Vaclavkova, P. Dzubak, I. Vrobel, P. Pouckova, J. Sedlacek, A. Miklovicova, A. Kutt, J. Li, J. Mattova, C. Driessen, Q. P. Dou, J. Olsen, M. Hajduch, B. Cvek, R. J. Deshaies, J. Bartek, Nature 2017, 552, 194–199. 29211715 10.1038/nature25016 PMC5730499

[4] During the course of our studies the marketing of sodium aurothiomalate (ATM) was ended by several pharma companies (https://go.drugbank.com/drugs/DB09276; retrieved on April 9th 2021).

[5a] E. R. T. Tiekink, Inflammopharmacology 2008, 16, 138–142; 18521545 10.1007/s10787-007-0018-5

[5b] E. R. T. Tiekink, Crit. Rev. Oncol. Hematol. 2002, 42, 225–248; 12050017 10.1016/s1040-8428(01)00216-5

[5c] C. I. Yeo, K. K. Ooi, E. R. T. Tiekink, Molecules 2018, 23, 1410; 10.3390/molecules23061410 PMC6100309 29891764

[5d] J. H. Kim, E. Reeder, S. Parkin, S. G. Awuah, Sci. Rep. 2019, 9, 12335. 31451718 10.1038/s41598-019-48584-5 PMC6710276

[6] For conceptionally related work using DSF in combination with auranofin see:

[6a] R. E. Kast, J. A. Boockvar, A. Brüning, F. Cappello, W.-W. Chang, B. Cvek, Q. P. Dou, A. Duenas-Gonzalez, T. Efferth, D. Focosi, S. H. Ghaffari, G. Karpel-Massler, K. Ketola, A. Khoshnevisan, D. Keizman, N. Magné, C. Marosi, K. McDonald, M. Muñoz, A. Paranjpe, M. H. Pourgholami, I. Sardi, A. Sella, K. S. Srivenugopal, M. Tuccori, W. Wang, C. R. Wirtz, M.-E. Halatsch, Oncotarget 2013, 4, 502–530; 23594434 10.18632/oncotarget.969 PMC3720600

[6b] H. Huang, Y. Liao, N. Liu, X. Hua, J. Cai, C. Yang, H. Long, C. Zhao, X. Chen, X. Lan, D. Zang, J. Wu, X. Li, X. Shi, X. Wang, J. Liu, Oncotarget 2016, 7, 2796–2808. 26625200 10.18632/oncotarget.6425 PMC4823072

[7a] M. E. Davis, M. E. Brewster, Nat. Rev. Drug Discov. 2004, 3, 1023–1035; 15573101 10.1038/nrd1576

[7b] B. dos Santos Lima, S. Shanmugam, J. De Souza Siqueira Quintans, L. J. Quintans Jr., A. A. De Souza Araújo, Phytochem. Rev. 2019, 18, 1337–1359;

[7c] S. R. Gandhi, J. De Souza Siqueira Quintans, G. R. Gandhi, A. A. De Souza Araújo, L. J. Quintans Jr. , Expert Opin. Drug Deliv. 2020, 17, 1069–1080. 32515613 10.1080/17425247.2020.1776261

[8a] D. D. Heinrich, J.-C. Wang, J. P. Fackler Jr. , Acta Crystallogr. 1990, C46, 1444–1447;

[8b] D. Paliwoda, P. Wawrzyniak, A. Katrusiak, J. Phys. Chem. Lett. 2014, 5, 2182–2188; 26279531 10.1021/jz500778t

[8c] The precipitate was analyzed by MALDI-MS and elemental analysis. Furthermore, the precipitate was recrystallized from hot DMF affording orange needles that were identified as complex **5** by X-ray crystallography (see reference 8b). IR-spectra of both the precipitate and recrystallized material are identical (see Figure S1) confirming that the product was not transformed during the recrystallization procedure.

[9] D. J. Lewis, P. Deshmukh, A. A. Tedstone, F. Tuna, P. O′Brien, Chem. Commun. 2014, 50, 13334–13337. 10.1039/c4cc04767b 25233190

[10a] L. Ronconi, L. Giovagnini, C. Marzano, F. Bettìo, R. Graziani, G. Pilloni, D. Fregona, Inorg. Chem. 2005, 44, 1867–1881; 15762713 10.1021/ic048260v

[10b] X. Zhang, M. Frezza, V. Milacic, L. Ronconi, Y. Fan, C. Bi, D. Fregona, Q. P. Dou, J. Cell. Biochem. 2010, 109, 162–172; 19911377 10.1002/jcb.22394 PMC3767936

[10c] M. Altaf, M. Monim-ul-Mehboob, A. A. Isab, V. Dhuna, G. Bhatia, K. Dhuna, S. Altuwaijri, New J. Chem. 2015, 39, 377–385;

[10d] A. A. A. Sulaiman, M. Altaf, A. A. Isab, A. Alawad, S. Altuwaijri, S. Ahmad, Z. Anorg. Allg. Chem. 2016, 642, 1454–1459;

[10e] C. K. Adokoh, RSC Adv. 2020, 10, 2975–2988. 10.1039/c9ra09682e PMC9048446 35496096

[11a] S. Jacob, A. B. Nair, Drug Dev. Res. 2018, 79, 201–217; 30188584 10.1002/ddr.21452

[11b] M. Fukuda, D. A. Miller, N. A. Peppas, J. W. McGinity, Int. J. Pharm. 2008, 350, 188–196. 17920217 10.1016/j.ijpharm.2007.08.038

[12a] H. J. A. Blaauw, R. J. F. Nivard, G. J. M. Van Der Kerk, J. Organomet. Chem. 1964, 2, 236–244;

[12b] J. Dobrowolski, Z. Badkowska, I. Kwiatkowska, Roczniki Chemii, Ann. Soc. Chim. Polonorum 1976, 50, 53–60;

[12c] J. B. Miller, J. L. Burmeister, Synth. React. Inorg. Met.-Org. Chem. 1985, 15, 223–233;

[12d] P. Bishop, P. Marsh, A. K. Brisdon, B. J. Brisdon, M. F. Mahon, J. Chem. Soc. Dalton Trans. 1998, 675–682;

[12e] M. A. Mansour, W. B. Connick, R. J. Lachicotte, H. J. Gysling, R. Eisenberg, J. Am. Chem. Soc. 1998, 120, 1329–1330.

[13] S. Han, O.-S. Jung, Y.-A. Lee, Transition Met. Chem. 2011, 36, 691–697.

[14a] F. Baril-Robert, M. A. Radtke, C. Reber, J. Phys. Chem. C 2012, 116, 2192–2197;

[14b] S. Naeem, S. A. Serapian, A. Toscani, A. J. P. White, G. Horgarth, J. D. E. T. Wilton-Ely, Inorg. Chem. 2014, 53, 2404–2416. 24512628 10.1021/ic402048a

[15a] E. Amtmann, N. Gunkel, A. K. Miller, M. Morgen, “Compositions Comprising a Metal Source, Dithiocarbamate and Cyclodextrin”, WO 2018/069525, **2018**;

[15b] E. Amtmann, N. Gunkel, A. K. Miller, M. Morgen, “Improved Dithiocarbamate Based Compounds, Therapies and Diagnostics”, WO 2020/161337, **2020**.

[16] H. Schmidbaur, A. Schier, Chem. Soc. Rev. 2008, 37, 1931–1951. 18762840 10.1039/b708845k

[17] The experimental structures are depicted using Mercury 4.2.0 (http://www.ccdc.cam.ac.uk/mercury/).

[18] τ is shown to adopt values between 0° and ∼34° (see Table S1).

[19a] L. X. Song, L. Bai, X. M. Xu, J. He, S. Z. Pan, Coord. Chem. Rev. 2009, 253, 1276–1284;

[19b] F. Hapiot, S. Tilloy, E. Monflier, Chem. Rev. 2006, 106, 767–781; 16522008 10.1021/cr050576c

[19c] D. Prochowicz, A. Kornowicz, J. Lewiński, Chem. Rev. 2017, 117, 13461–13501. 29048880 10.1021/acs.chemrev.7b00231

[20a] W. Saenger, T. Steiner, Acta Cryst. 1998, A54, 798–805;

[20b] J. Szejtli, Encyclopedia Nanosci. Nanotechnol. 2004, 2, 283–304;

[20c] G. Crini, Chem. Rev. 2014, 114, 10940–10975. 25247843 10.1021/cr500081p

[21] H.-J. Schneider, F. Hacket, V. Rüdiger, H. Ikeda, Chem. Rev. 1998, 98, 1755–1785. 11848948 10.1021/cr970019t

[22] A fourth, less well-resolved X-ray structure of the inclusion complex [**37**/(β-CD)_2_] was also obtained (see Figure S6).

[23] A thorough CCDC search on experimental structures with (plain) β-cyclodextrin performed on November 24th 2020 revealed 336 entries. We identified 107 entries of inclusion complexes with a 1 : 1 host/guest ratio (of which 9 inclusion complexes were host/metal complexes), 16 entries with a 2 : 1 host/guest ratio, 38 entries with a 2 : 2 host/guest ratio, 1 entry with a 3 : 2 host/guest ratio, and 33 entries which we considered encapsulation interactions of β-cyclodextrin with the guest molecule. Among the sixteen 2 : 1 host/guest complexes, only 2 entries showed a “tail-to-tail” arrangement of the CD cones (CCDC reference codes OCIGAK and SATFEB). In this evaluation we only included inclusion complexes with small organic or metal-organic guest molecules – solvates or salt adducts of β-cyclodextrin were excluded.

[24] S. J. Berners-Price, A. Filipovska, Metallomics 2011, 3, 863–873. 21755088 10.1039/c1mt00062d

[25] M. Gutscher, M. C. Sobotta, G. H. Wabnitz, S. Ballikaya, A. J. Meyer, Y. Samstag, T. P. Dick, J. Biol. Chem. 2009, 284, 31532–31540. 19755417 10.1074/jbc.M109.059246 PMC2797222

[26] P. Jansook, N. Ogawa, T. Loftsson, Int. J. Pharm. 2018, 535, 272–284. 29138045 10.1016/j.ijpharm.2017.11.018

[27a] T. Loftsson, A. Magnúsdóttir, M. Másson, J. F. Sigurjónsdóttir, J. Pharm. Sci. 2002, 91, 2307–2316; 12379916 10.1002/jps.10226

[27b] T. Loftsson, M. Másson, H. H. Sigurdsson, Int. J. Pharm. 2002, 232, 35–43. 11790488 10.1016/s0378-5173(01)00895-x

[28] D. Ezeriņa, Y. Takano, K. Hanaoka, Y. Urano, T. P. Dick, Cell Chem. Biol. 2018, 25, 447–459. 29429900 10.1016/j.chembiol.2018.01.011 PMC6455997

