# Supplementary material for: Inclusion Complexes of Gold(I)‐Dithiocarbamates with β‐Cyclodextrin: A Journey from Drug Repurposing towards Drug Discovery
Source: Chemistry. 2021 Jul 13;27(47):12156–65. doi: 10.1002/chem.202101366 (PMC8456977; doi:10.1002/chem.202101366)
Supplement: Supplementary file 1 — Supporting Information [file CHEM-27-12156-s001.pdf]

Chemistry–A European Journal

Supporting Information

Inclusion Complexes of Gold(I)-Dithiocarbamates with β-Cyclodextrin: A Journey from Drug Repurposing towards Drug Discovery

Michael Morgen, Piotr Fabrowski, Eberhard Amtmann, Nikolas Gunkel, and Aubry K. Miller*

Table of Contents

IR-spectra of [Au(N,N-diethyl)dtc]$_2$ (**5**) (Figure S1) ……………………….............page S2

Dose-Response Curves of ATM/DSF and **5** (Figure S2) ………………….............page S3

Nephelometric Solubility Measurements (Figure S3) ……………………………..page S4

NMR Spectra of the Inclusion Complexes (Figure S4) ……………………………page S5

Determination of Inclusion Complex Structural Parameters (Figure S5) ………....page S6

Experimental Structure of the Inclusion Complex [**37**/(β-CD)$_2$] (Figure S6) ……..page S7

CCDC-Database Search of Experimental Gold(I)-dtc Complexes (Table S1) …….page S8

Crystal and structure refinement data of **17** and **23** ………………………………..page S10

Crystal and structure refinement data of β-cyclodextrin inclusion complexes …....page S11

Syntheses of the Gold(I)-dtc Complexes …………………………………………..page S12

References ……………………………………………………………………….page S28

NMR Spectra of the Gold(I)-dtc Complexes ………………………………………page S29

IR spectra of [Au(*N*, *N*-diethyl)dtc]$_2$ (5)

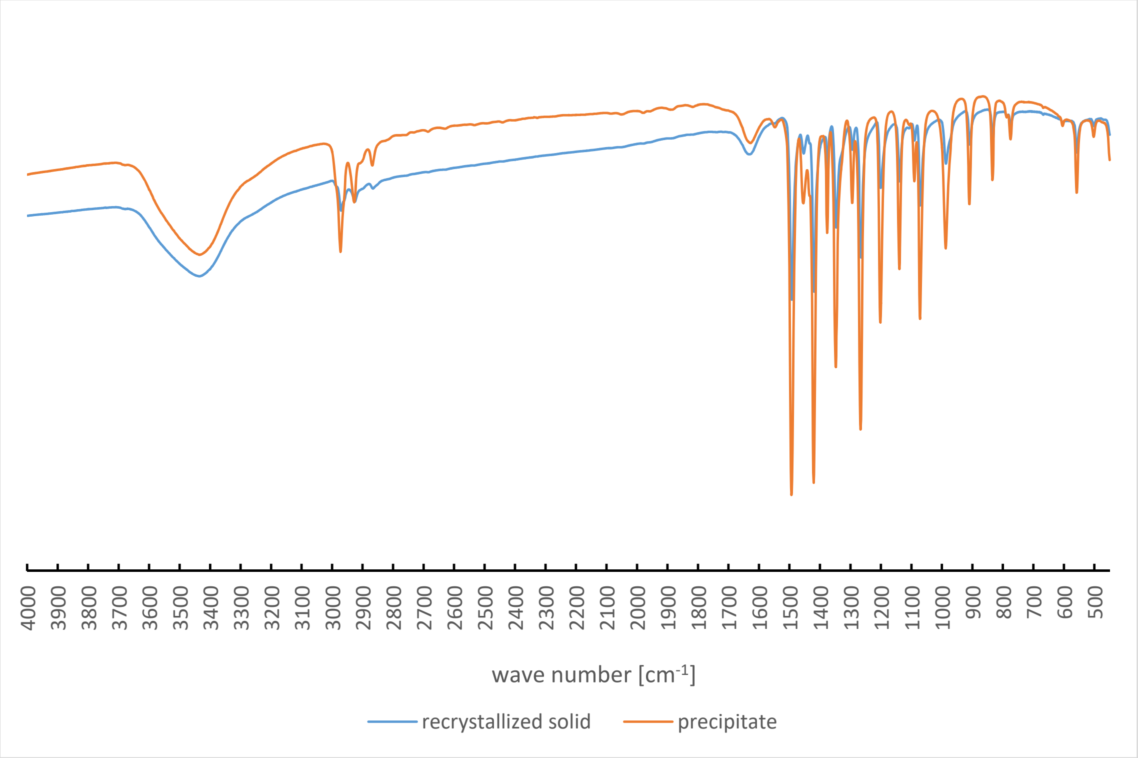

Figure S1: Solid state IR-spectra (KBr pellet) of the isolated reaction product from ATM and DSF (1:1); see Scheme 1. The spectrum of the obtained precipitate (orange) is identical to the spectrum of the material obtained by recrystallization from hot DMF (blue) that was identified as gold(I)-dtc complex **5** by X-ray crystallography.

Dose-Response Curves of ATM/DSF and 5

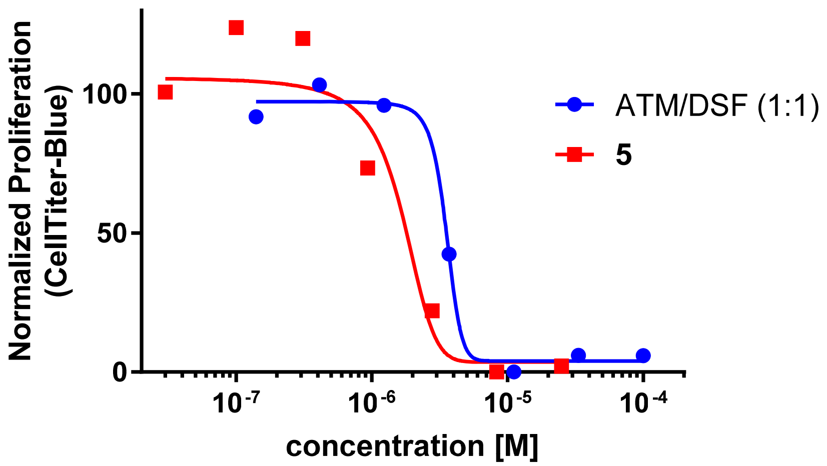

Figure S2: Dose-response curves of the stoichiometric mixture of ATM and DSF (blue) as well as isolated **5** (red) for the treatment of Calu-6 cells (both in the presence of 30-fold excess of 2-hydroxypropyl-β-CD).

Nephelometric Solubility Measurements

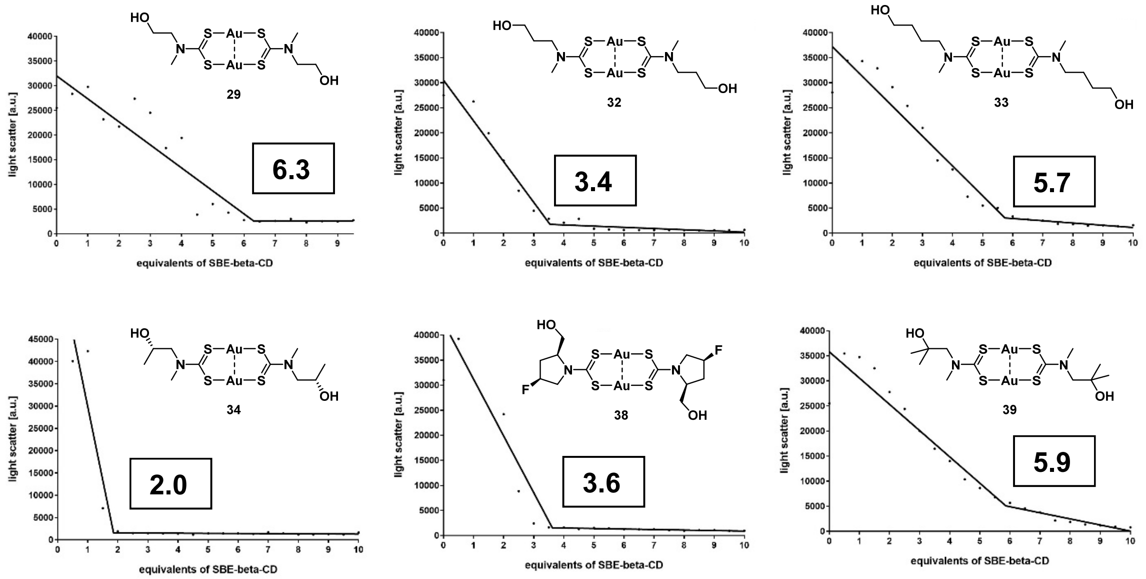

Figure S3: Nephelometric solubility data of gold(I)-dtc complexes **29**, **32**, **33**, **34**, **38** and **39**. A precipitate formed in each well where the concentration of SBE-β-CD was too low in order to solubilize the (fixed) amount of gold(I)-dtc complex. The light scattering intensity is proportional to the amount of precipitation. A visual inspection of the nephelo well-plate was necessary to identify wells with bubble formation – these data points were neglected in the later analysis. Stock solutions (50 mM) of the gold(I)-dtc complexes in DMSO were prepared using a heated sonication bath (56 °C). A 10 mM stock solution of sulfobutylether-β-cyclodextrin (SBE-β-CD, captisol®) in PBS (gibco, DPBS, Dulbecco's Phosphate Buffered Saline; Life Technologies, Ltd., UK) was diluted in PBS to make 2 mL aliquots of SBE-β-CD in 21 different concentrations ranging from 10 mM to 0 mM (in 0.5 mM increments), of which 100 µL were transferred into the assay wells of a 384 well plate (Corning ® black/clear flat bottom polysterene, not treated microplate, low flange, without lid, nonsterile; product number 3762). To each well was added 2 µL of the complex stock solution. A manual 16-channel Finnpipette (Thermo Scientific) was used for mixing by pipetting up and down (eight times). Finally, 40 µL of each well were transferred into the 384well Nephelo-plate and measured. Measurements were performed on a Nephelostar Galaxy (BMG Labtech). Raw data (light scatter intensities) were recorded with the corresponding software version 4.30-0; subsequent data processing was done with GraphPad Prism (Version 7.05) using the segmental linear regression model.

NMR Spectra of the Inclusion Complexes

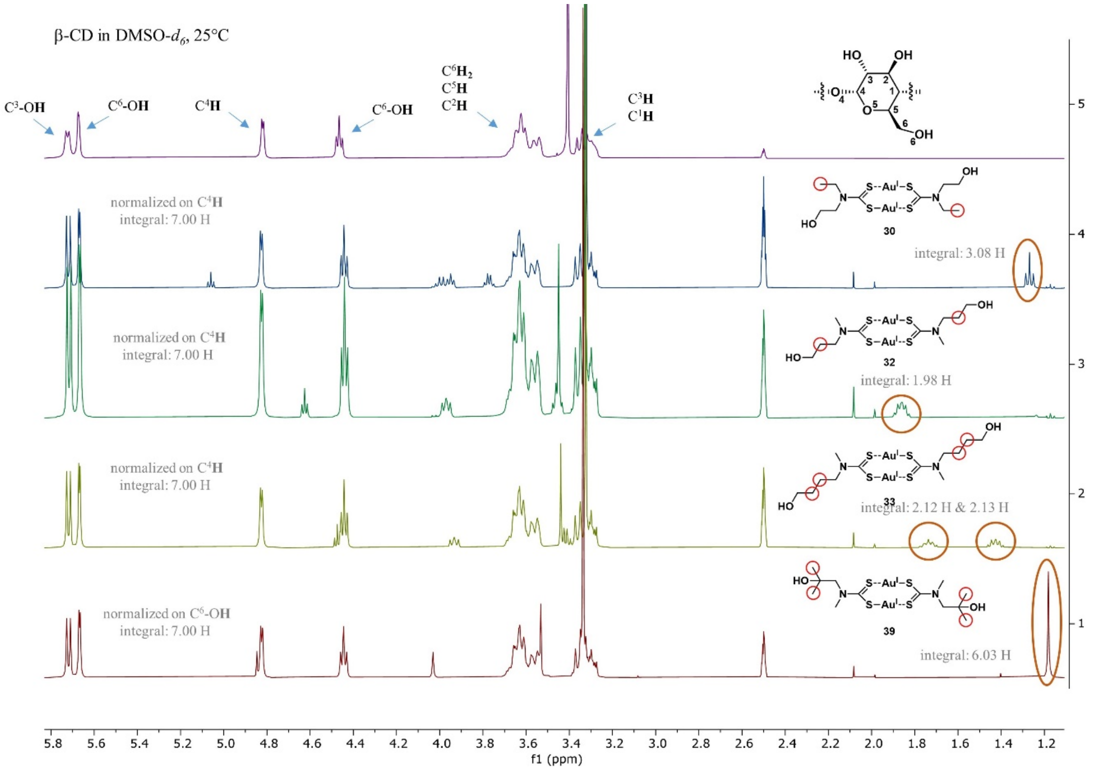

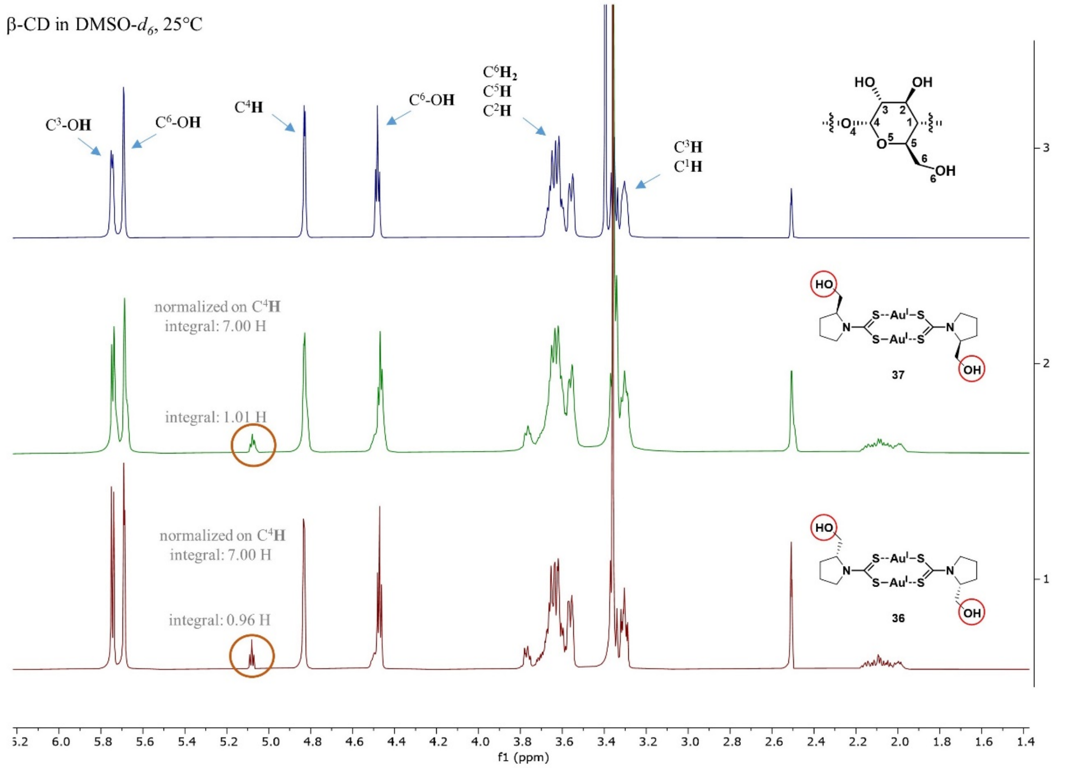

Figure S4: NMR spectra of β-CD and the crystalline material obtained by co-crystallization of β-CD and gold(I)-dtc complexes **30**, **32**, **33** and **39** (top) as well as **36** and **37** (bottom). The

NMR spectra show a distinct 2:1 host–guest ratio. However, a difference of the chemical shifts of β-CD or the gold(I)-dtc complexes compared to the individual compounds was not observed. This is indicative for a dissociation of the inclusion complexes in DMSO that might successfully displace the organic ligands from the cavity of the CD cones.[1]

Determination of Inclusion Complex Structural Parameters

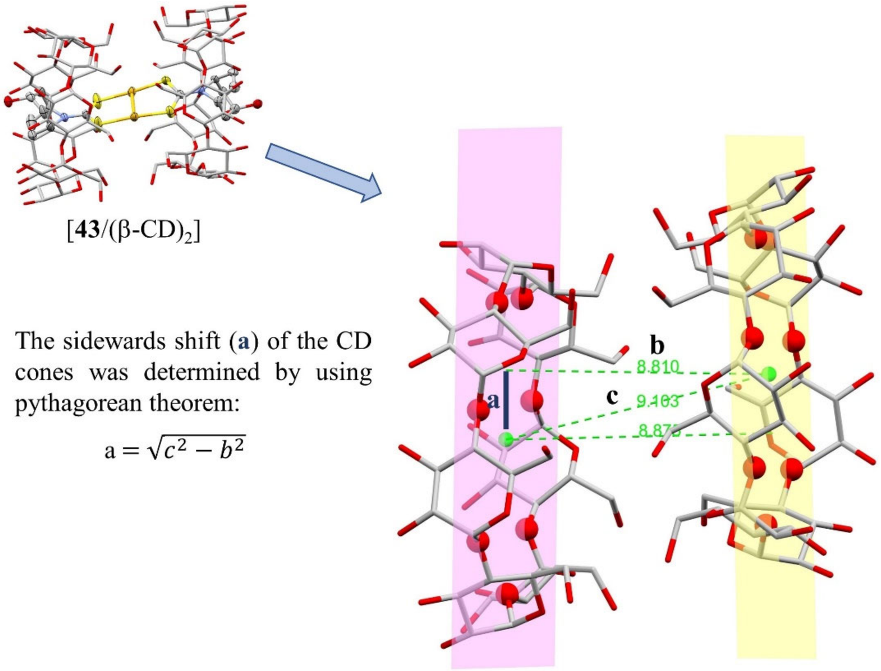

Figure S5: Determination of the structural parameters for the description of the arrangement of the CD cones within the inclusion complexes on the example of [**43**/(β-CD)$_2$] using Mercury.[2] For clarity the gold(I)-dtc complex was deleted. For each CD cone a mean plane (purple and yellow, respectively) through all seven glycosidic oxygens (highlighted as red balls) as well as the corresponding centroid (green balls) were defined. The angle between the CD cones is defined as the angle between both formerly defined planes. The sidewards shift of the CD cones is calculated using the Pythagorean theorem as depicted above – the mean of both possible values is reported (the difference in both values is a result of the angle between both planes).

Experimental Structure of the Inclusion Complex [37/(β-CD)$_2$]

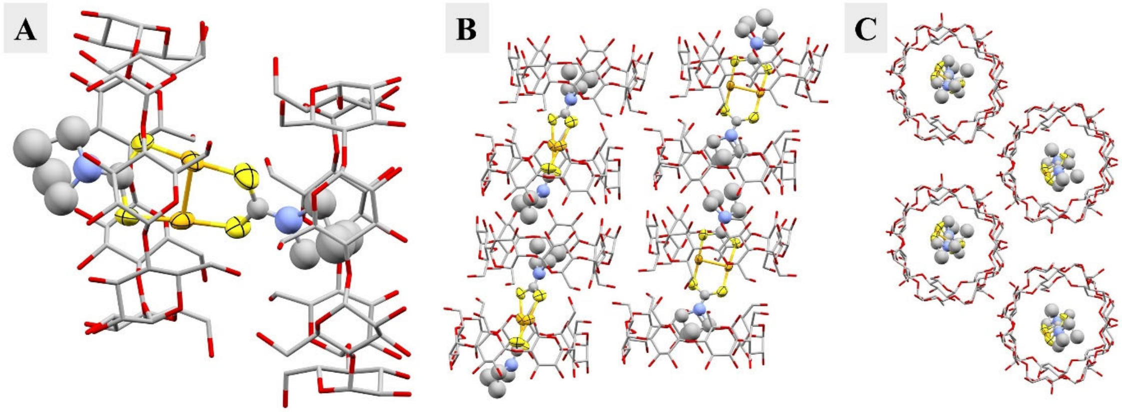

Figure S6: A. Depiction of the experimental structure of [**37**/(β-CD)$_2$]. **B.** Lateral view. **C.** Axial view. Hydrogens and water molecules are omitted for clarity and the β-cyclodextrin cones are shown as capped-stick model whereas the gold(I)-dtc complex **37** is shown in ellipsoids (on a 50% probability level). Color code: carbon, grey; oxygen, red; nitrogen, blue; sulfur, yellow; gold, orange.[2]

Table S1: CCDC-Database Search of Experimental Gold(I)-dtc Complexes.

Entry	Compound Name (as appearing in CCDC database)	Stacking of eight-membered chelate ring	Twist angle τ [°]*,§	S-Au-S [°]#,§	Au-Au [Å]§	Database Identifier	Deposition Number	Associated Reference
1	catena-(bis(μ2-Diethyldithiocarbamate)-di-gold)	Linear, perpendicular	27.2<br>27.5<br>15.8 / 30.7<br>13.9 / 31.1<br>14.5 / 32.2<br>13.6 / 32.6<br>27.0<br>27.9	176.8<br>177.7<br>173.3 / 174.9<br>173.4 / 176.8<br>172.4 / 175.0<br>171.7 / 175.3<br>176.7<br>176.5	2.79<br>2.78<br>2.77 / 2.76<br>2.76 / 2.73<br>2.74 / 2.72<br>2.71 /2.75<br>2.79<br>2.78	JEKJOY01<br>JEKJOY02<br>JEKJOY03<br>JEKJOY04<br>JEKJOY05<br>JEKJOY06<br>JEKJOY07<br>JEKJOY08	980959<br>980960<br>980962<br>980963<br>980964<br>980965<br>980958<br>1417942	Paliwoda *et al.*[3]
2	catena-[bis(μ2-Diethyldithiocarbamato-gold(i)) dichloromethane solvate]	Linear, perpendicular	26.9<br>22.2 / 31.9 / 31.0 / 19.1	176.6<br>175.1 / 177.6 / 176.8 / 174.4	2.79<br>2.78 / 2.77 / 2.76 / 2.78	VOJBUT01<br>VOJBUT02	980957<br>1996352	Paliwoda *et al.*[3];
3	bis(μ2-di-*n*-propyldithiocarbamato)-di-gold(i)	Linear, coplanar	37.5<br>33.9	178.1<br>179.7	2.81<br>2.76	AUDTHC11<br>AUDTHC10	203752<br>1103883	Ho *et. al.*[4]; Hesse *et al.*[5]
	[Au^{I}(4-methylpiperazinyl)dtc]$_2$ **23**	Linear, coplanar	33.6	179.7	2.81		2068757	**This work**
4	catena-[bis(μ2-Diprop-2-en-1-ylcarbamodithioato)-di-gold]	Linear, perpendicular	27.5	176.8	2.79	AHARUY	830718	Naeem *et al.*[6]
5	bis(μ2-*N*,*N*-Dipentyl-dithiocarbamato-S,S')-di-gold(i) dimethylsulfoxide solvate	Linear, perpendicular	26.7 / 33.8	176.9 / 178.8	2.78 / 2.77	PUCVIS	1238709	Mansour *et al.*[7]
6	bis(μ2-*N*,*N*-Dipentyl-dithiocarbamato-S,S')-di-gold(i) acetonitrile solvate	Linear, helical				PUCVUE	1238711	Mansour *et al.*[7]
7	catena(bis(μ2-*N*,*N*-bis(Methoxyethyl)-dithiocarbamato-S,S')-di-gold(i))	Linear, twisted	35.0	174.0	2.79	PUCZUI	1238727	Bishop *et al.*[8]
8	catena-[bis(μ-1,4,7,10,13-pentaoxa-16-azacyclooctadecane-16-carbodithioato)-di-gold acetonitrile solvate]	Linear, (almost) perpendicular	3.64	170.2	2.72	GOXFIK<br>GOXFOQ<br>NORTEV	1027267<br>1027268<br>1027266	Tzeng *et al.*[9]
9	catena-[tetrakis(μ-(2,6-dimethylphenyl)((2,6-dimethylphenyl)carbonoimidoyl)carbamodithioato)-tetra-gold]	staggered	15.1 / 26.5	172.6 / 175.7	2.76 / 2.76	TUVPAD	1008664	Lane *et al.*[10]
10	bis(μ2-(Methyl(2-(pyridin-2-yl)ethyl)carbamodithioato))-di-gold isophthalic acid	Staggered (co-crystallized with isophthalic acid)	14.8 / 15.7	171.5 / 172.0	2.75 / 2.78	EYURAS	813945	Han *et al.*[11]
11	bis(μ2-(Aza-15-crown-5)dithiocarbamato)-di-gold(i)	**Isolated** (no intermolecular aurophilic bonding)	0.2	171.9	2.78	EBIWAO	218770	Tzeng *et al.*[12]
12	bis(μ-dimethylcarbamodithioato)-di-gold bis(bis(dimethylcarbamodithioato)-gold) bis(hexafluorophosphate)	**Isolated** (co-crystallized with Au(III) complex)	1.0	171.9	2.78	UBALOB	1063074	Langaro *et al.*[13]

13	hexakis(μ_2-*N*-(3,6,9,12-Tetraoxatetradecane-1,14-diyl)dithiocarbamato)-hexa-gold(i)	No eight-membered ring chelate	–	–	–	VOCRUB	645163	Arias *et al.*[14]
	[Au^I(*N*,*N*-dibenzyl)dtc]$_2$ **17**	**Isolated**	2.3	171.9	2.81		2068758	**This work**

* τ can be calculated by taking the mean of both dihedral angles S(1)-Au(1)-Au(2)-S(2) and S(3)-Au(2)-Au(1)-S(4); see Figure 4.

# reported is the mean value of both angles S(1)-Au(1)-S(4) and S(2)-Au(2)-S(3); see Figure 4.

§ in deposited structures with two individual gold(I)-dtc complexes found in the asymmetric unit (with different structural parameters) both values are reported.

Table S2: Crystal and structure refinement data of gold(I)-dtc complexes **17** and **23**.

	**17**		**23**	
**Crystal Data**				
Chemical formula	$C_{30}H_{28}Au_2N_2S_4$		$C_{12}H_{22}Au_2N_4S_4$	
MW [g/mol]	938.72		744.51	
Temperature [K]	200(2)		200(2)	
Crystal system, space group	monoclinic, *P*2$_1$/c		monoclinic, *C*2/c	
Unit cell dimensions [Å, °]	a = 6.5798(5)	α = 90	a = 19.7541(13)	α = 90
	b = 13.6628(10)	β = 92.2018(10)	b = 6.0457(4)	β = 105.1442(12)
	c = 16.2717(12)	γ = 90	c = 15.7288(10)	γ = 90
Volume [Å^3]	1461.72(19)		1813.2(2)	
Density (calculated) [g/cm^3]	2.133		2.727	
Z	2		4	
Radiation type	Mo K$_\alpha$, λ = 0.71073 Å			
Absorption coefficient μ [mm^{-1}]	10.334		16.624	
Crystal shape/color	brick/yellow		needle/yellow	
Crystal size [mm^3]	0.108 x 0.096 x 0.065		0.226 x 0.093 x 0.056	
**Structure Refinement**				
Theta range for data collection	1.9 to 30.9 deg		2.1 to 26.4 deg	
Index ranges	-8 ≤ h ≤ 8,		-24 ≤ h ≤ 24,	
	-19 ≤ k ≤ 19,		-7 ≤ k ≤ 7,	
	-22 ≤ l ≤ 22		-15 ≤ l ≤ 19	
Reflections collected	18578		6051	
Independent reflections	4242 (R(int) = 0.0335)		1869 (R(int) = 0.0261)	
Observed reflections	3076 (I > 2 σ (I))		1693 (I > 2 σ (I))	
Absorption correction	Semi-empirical from equivalents			
Max. and min. transmission	0.56 and 0.46		0.49 and 0.31	
Refinement method	Full-matrix least-squares on F^2			
Data/restraints/parameters	4242 / 0 / 172		1869 / 0 / 102	
Goodness-of-fit on F^2	1.02		1.08	
Final R indices (I>2sigma(I))	R1 = 0.024, wR2 = 0.044		R1 = 0.018, wR2 = 0.038	
Largest diff. peak and hole [eÅ^{-3}]	0.78 and -0.89		0.62 and -1.08	

Table S3: Crystal and structure refinement data of the inclusion complexes of **33**, **37**, **39**, **43** with β-cyclodextrin.

	[**33**/(β-CD)$_2$]		[**37**/(β-CD)$_2$]		[**39**/(β-CD)$_2$]		[**43**/(β-CD)$_2$]	
**Crystal Data**								
Chemical formula	$C_{96}H_{164}Au_2N_2O_{88}S_4$		$C_{94}H_{98}Au_2N_2O_{83}S_4$		$C_{96}H_{164}Au_2N_2O_{96}S_4$		$C_{100.81}H_{165.65}Au_2N_2O_{104.06}S_4$	
MW [g/mol]	3276.45		3105.91		3404.46		3592.85	
Temperature [K]	200(2)		200(2)		200(2)		200(2)	
Crystal system, space group	Triclinic, *P*1		Monoclinic, *P*2$_1$		Orthorhombic, *C*222$_1$		Triclinic, *P*1	
Unit cell dimensions [Å, °]	a = 15.1527(6)	α = 87.237(3)	a = 15.5987(9)	α = 90	a = 19.3358(5)	α = 90	a = 5.1082(4)	α = 90.432(2)
	b = 15.4306(7)	β = 81.827(3)	b = 24.3169(9)	β = 110.918(4)	b = 24.1261(6)	β = 90	b = 5.7119(5)	β = 111.501(2)
	c = 15.7202(6)	γ = 76.744(3)	c = 18.9348(10)	γ = 90	c = 1.3324(11)	γ = 90	c = 8.2600(5)	γ = 106.547(2)
Volume [Å^3]	3540.9(3)		6708.8(6)		28611.4(11)		3835.3(2)	
Density (calculated) [g/cm^3]	1.54		1.54		1.58		1.56	
Z	1		2		8		1	
Radition type	Cu K$_\alpha$, λ = 1.54178 Å							
Absorption coefficient μ [mm^{-1}]	5.35		5.60		5.37		5.08	
Crystal shape/color	plate/colorless		Plate/colorless		brick/colorless		plank/colorless	
Crystal size [mm^3]	0.068 x 0.063 x 0.024		0.060 x 0.055 x 0.015		0.078 x 0.046 x 0.041		0.150 x 0.031 x 0.015	
**Structure Refinement**								
Theta range for data collection	4.4 to 57.9 deg		2.5 to 47.2 deg		2.9 to 57.9 deg		2.6 to 67.0 deg	
Index ranges	$-16 \le h \le 16$,		$-14 \le h \le 14$		$-16 \le h \le 21$,		$-17 \le h \le 17$,	
	$-16 \le k \le 16$,		$-23 \le k \le 23$		$-26 \le k \le 18$,		$-9 \le k \le 18$,	
	$-17 \le l \le 17$		$-11 \le l \le 18$		$-67 \le l \le 63$		$-21 \le l \le 21$	
Reflections collected	35665		26387		58456		32245	
Independent reflections	9336 (R(int) = 0.0619)		11004 (R(int) = 0.1641)		18416 (R(int) = 0.1187)		14881 (R(int) = 0.0400)	
Observed reflections	4749 (I > 2 σ (I))		4975 (I > 2 σ (I))		12216 (I > 2 σ (I))		11676 (I > 2 σ (I))	
Absorption correction	Semi-empirical from equivalents							
Max. and min. transmission	1.94 and 0.56		1.68 and 0.46		2.06 and 0.48		1.69 and 0.61	
Refinement method	Full-matrix least-squares on F^2							
Data/restraints/parameters	35665 / 5512 / 1789		11004 / 5284 / 1643		18416 / 4288 / 1847		14881 / 1483 / 1978	
Goodness-of-fit on F^2	0.91		0.94		1.07		0.98	
Final R indices (I>2sigma(I))	R1 = 0.071, wR2 = 0.176		R1 = 0.092, wR2 = 0.210		R1 = 0.083, wR2 = 0.190		R1 = 0.041, wR2 = 0.096	
Absolute structure parameter	0.011(6)		0.243(12)		0.162(7)		0.014(6)	
Largest diff. peak and hole [eÅ^{-3}]	0.87 and -0.89		1.39 and -0.76		1.33 and -0.85		0.75 and -0.74	

Syntheses of the Gold(I)-dtc Complexes[15]

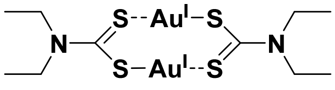

$C_{10}H_{20}Au_2N_2S_4$
M = 690.46 g/mol

[Au^I(*N,N*-diethyl)dtc]$_2$ 5: A solution of ATM (2.0 g, 5.13 mmol, 1.0 equiv.), dissolved in 80.0 mL of water, and a solution of sodium diethyldithiocarbamate trihydrate (1.39 g, 6.15 mmol, 1.2 equiv.), dissolved in 20.0 mL of water, were combined and vigorously stirred at room temperature over night. The obtained orange powder was filtered and thoroughly washed with water and dried under high vacuum over night (1.55 g, 2.24 mmol, 87%). This material was recrystallized from hot DMF (approximately 500 mL) to afford orange needles as the pure product in a yield of 82% (1.46 g, 2.11 mmol). **HRMS-ESI** (*m/z*) [Au^{III} + 2 dtc$^-$]$^+$ calcd. for $C_{10}H_{20}AuN_2S_4$: 493.0175; Found: 493.0170. **Anal.** calcd for $C_{10}H_{20}Au_2N_2S_4$: C, 17.39; H, 2.92; N, 4.06; Found: C, 17.45; H, 3.02; N, 4.22.

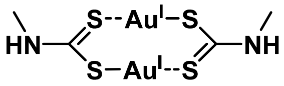

$C_4H_8Au_2N_2S_4$
M = 606.30 g/mol

[Au^I(*N*-methyl)dtc]$_2$ 6: A solution of ATM (352.7 mg, 0.9 mmol, 1.0 equiv.) and a solution of metam (174.4 mg, 1.35 mmol, 1.5 equiv.), dissolved in 10.0 mL of water each, were combined and vigorously stirred at room temperature for 2 h. The obtained slightly greenish powder was thoroughly washed with water, a small amount of ethanol and diethyl ether successively and dried under high vacuum over night. The product was obtained in a yield of 68% (183.6 mg, 0.30 mmol). **^{1}H NMR** (400 MHz, DMSO-d_6) δ 10.71 (q, J = 5.2 Hz, 2H), 3.02 (d, J = 4.7 Hz, 6H) ppm. **^{13}C NMR** (101 MHz, DMSO-d_6) δ 204.4, 35.7 ppm. **HRMS-ESI** (*m/z*) [Au^{III} + 2 dtc$^-$]$^+$ calcd. for $C_4H_8AuN_2S_4$: 408.9236; Found: 408.9232. **Anal.** calcd. for $C_4H_8Au_2N_2S_4$: C, 7.92; H, 1.33; N, 4.62; Found C, 8.10; H, 1.49; N, 4.59.

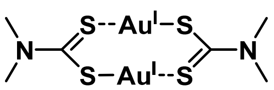

$C_6H_{12}Au_2N_2S_4$
M = 634.35 g/mol

[Au^I(*N,N*-dimethyl)dtc]$_2$ 7: Chloro(dimethyl sulfide)gold(I) (325.1 mg, 1.1 mmol, 1.0 equiv.) and dimethyldithiocarbamate (157.5 mg, 1.1 mmol, 1.0 equiv.) were dissolved in 20.0 mL of acetonitrile each. The solutions were combined upon which a yellow solid precipitated. The mixture was stirred at room temperature for 3 h and afterwards filtered through a sintered glass funnel (pore 4). The solid was thoroughly washed with water, a small amount of ethanol and diethyl ether successively and dried under high vacuum over night. The product was obtained as a yellow powder in a yield of 35% (121.8 mg, 0.19 mmol). **HRMS-ESI** (*m/z*) [Au^{III} + 2 dtc$^-$]$^+$ calcd. for $C_6H_{12}AuN_2S_4$: 436.9549; Found: 436.9561. **Anal.** calcd. for $C_6H_{12}Au_2N_2S_4$: C, 11.36; H, 1.91; N, 4.42; Found: C, 11.61; H, 2.00; N, 4.43.

Compound S1

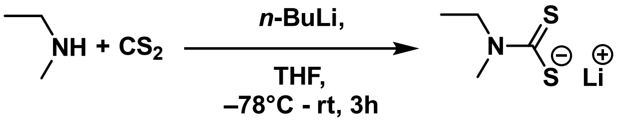

N-ethylmethyl amine (0.69 g, 1.0 mL, 11.67 mmol, 1.0 equiv.) was dissolved in 50.0 mL of dry THF and cooled to –78°C. Then *n*-butyllithium (2.5 M in hexane, 4.67 mL, 11.67 mmol, 1.0 equiv.) was added dropwise and the resulting mixture was stirred for 15 minutes whereupon carbon disulfide (0.89 g, 0.7 mL, 11.67 mmol, 1.0 equiv.) was added dropwise. The resulting mixture was stirred at –78°C for 15 minutes and afterwards allowed to warm up to room temperature. After a total reaction time of 3 h the solution was concentrated and dried under high vacuum over night. The product was obtained as a reddish solid in quantitative yield and was used in the next step without further purification. **^{1}H NMR** (400 MHz, D_2O) δ 4.09 (q, *J* = 7.2 Hz, 2H), 3.45 (s, 3H), 1.21 (t, *J* = 7.2 Hz, 3H) ppm.

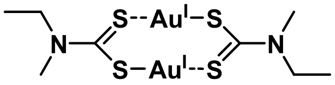

$C_8H_{16}Au_2N_2S_4$
M = 662.40 g/mol

[Au^I(*N*-ethyl, *N*-methyl)dtc]$_2$ 8: A solution of ATM (527.0 mg, 1.35 mmol, 1.0 equiv.) and a solution of lithium *N*-ethyl, *N*-methyl dithiocarbamate **S1** (286.6 mg, 2.03 mmol, 1.5 equiv.), dissolved in 10.0 mL of water each, were combined and vigorously stirred at room temperature over night. The obtained orange powder was thoroughly washed with water, a small amount of ethanol and diethyl ether successively and dried under high vacuum over night. The crude product was obtained in a yield of 73% (327.6 mg, 0.99 mmol). This material was further purified by precipitation from hot 1,2-dichloroethane to afford a fluffy, orange colored solid as the pure product. **HRMS-ESI** (*m/z*) [Au^{III} + 2 dtc$^-$]$^+$ calcd. for $C_8H_{16}AuN_2S_4$: 464.9862; Found: 464.9860. **Anal.** Calcd. for $C_8H_{16}Au_2N_2S_4$: C, 14.51; H, 2.43; N, 4.23; Found: C, 14.59; H, 2.66; N, 4.15.

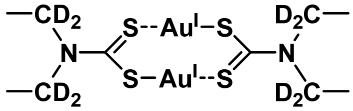

$C_{10}H_{12}D_8Au_2N_2S_4$
M = 698.52 g/mol

[Au^I(*N*,*N*-diethyl)dtc]$_2$-*d$_8$* 9: (i) α-deuteration of diethylamine:

Diethylamine (0.21 g, 0.30 mL, 2.9 mmol, 1.0 equiv.) and $RuCl_2(PPh_3)_3$ (200.0 mg, 0.21 mmol, 0.07 equiv.) were mixed in 4.0 mL of D_2O and heated under microwave irradiation at 150°C (internal pressure of 8 bar) for 3 h. After cooling to room temperature the suspension was filtered through syringe filter (Millex ®-HV, 0.45 µm, Merck-Millipore). The incorporation of deuterium was determined by ^{1}H-NMR (in D_2O) at this stage which was approx. 90%D (see Figure S7). An aliquot (2.0 mL) of this solution was used for the next step.[16]

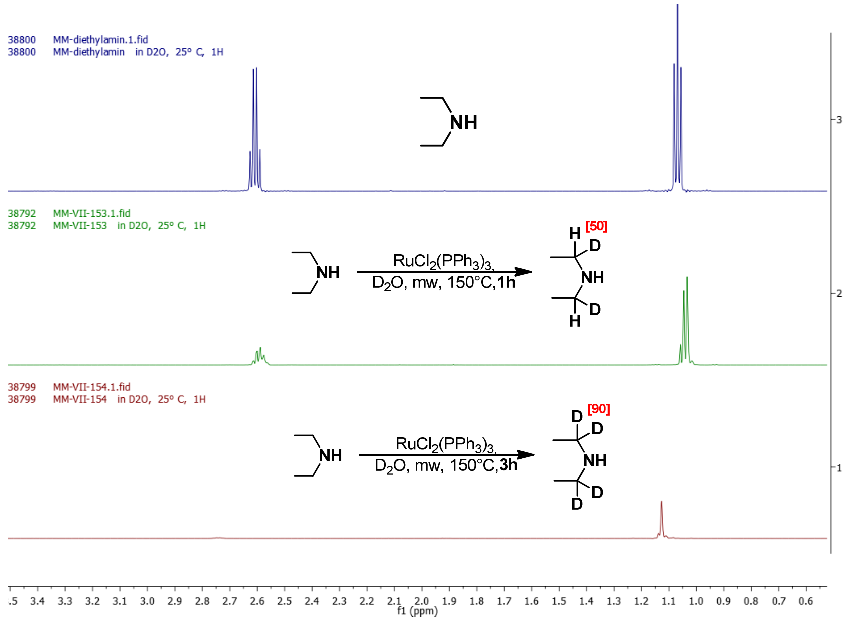

Determination of deuterium incorporation at the α-position of diethyl amine by ^{1}H NMR.

(ii) *In-situ* dithiocarbamate and subsequent complex formation

Diethylamine-d_4 (2.0 mL of D_2O-solution, ~1.45 mmol, ~1.2 equiv.) was diluted with 15.0 mL of water and potassium hydroxide (81.4 mg, 1.45 mmol, 1.2 equiv.) as well as carbon disulfide (110.4 mg, 0.09 mL, 1.45 mmol, 1.2 equiv.) were added to the mixture which was stirred at rt for 2 h. Then ATM (471.3 mg, 1.21 mmol, 1.0 equiv.) dissolved in 20.0 mL of H_2O was added to the mixture. An orange-colored precipitate formed immediately. The suspension was stirred at room temperature for 3 h and then filtered through a sintered glass funnel (pore 4). The resulting solid was thoroughly washed with H_2O (50 mL) and dried under high vacuum. The product was obtained as an orange-colored solid in a yield of 35% (294.6 mg, 0.42 mmol). **HRMS-ESI** (*m/z*) [Au^{III} + 2 dtc^-]$^+$ calcd. for $C_{10}H_{12}D_8AuN_2S_4$: 501.0677; Found: 501.0677. **Anal.** calcd. for $C_{10}H_{12}D_8Au_2N_2S_4$: C, 17.19; H, 2.89*; N, 4.01; Found: C, 17.31; H, 3.18; N, 3.96 (*a device-dependent correction factor f = 0.504 for the measurement of deuterium was applied).

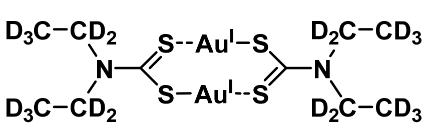

$C_{10}D_{20}Au_2N_2S_4$
M = 710.60 g/mol

[Au^I(*N,N*-diethyl)dtc]$_2$-d_{20} 10: The complex was synthesized according to the general procedure using diethanolamine-d_{10} (0.32 g, 0.40 mL, 3.85 mmol, 1.2 equiv.), potassium hydroxide (0.22 g, 3.85 mmol, 1.2 equiv.), carbon disulfide (0.24 g, 0.19 mL, 3.21 mmol, 1.0 equiv.) in 20.0 mL of water and ATM (1.25 g, 3.21 mmol, 1.0 equiv.) in 60.0 mL of water. A yellow solid was obtained as the pure product in a yield of 30% (678.0 mg, 0.95 mmol). **HRMS-ESI** (*m/z*) [M + Na]$^+$ calcd. for $C_{10}D_{20}Au_2N_2NaS_4$: 733.0988; Found: 733.0993. **Anal.** calcd. for $C_{10}D_{20}Au_2N_2S_4$: C, 16.90; H,

2.86*; N, 3.94; Found: C, 17.02; H, 3.14; N, 4.04 (*a device-dependent correction factor f = 0.504 for the measurement of deuterium was applied).

Compound S2

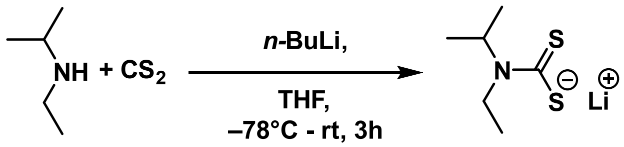

N-ethyl, *N*-isopropyl amine (0.72 g, 1.0 mL, 8.26 mmol, 1.0 equiv.) was dissolved in 20.0 mL of dry THF and cooled to –78°C. Then *n*-butyllithium (2.5 M in hexane, 3.30 mL, 8.26 mmol, 1.0 equiv.) was added dropwise and the resulting mixture was stirred for 15 minutes whereupon carbon disulfide (0.63 g, 0.5 mL, 8.26 mmol, 1.0 equiv.) was added dropwise. The resulting mixture was stirred at –78°C for 15 minutes and afterwards allowed to warm up to room temperature. After a total reaction time of 3 h the solution was concentrated and dried under high vacuum over night. The product was obtained as an off-white solid in quantitative yield and was used for the next step without further purification. **^{1}H NMR** (400 MHz, D_2O) δ 5.86 (hept, *J* = 6.8 Hz, 1H), 3.90 (q, *J* = 7.1 Hz, 2H), 1.28 (t, *J* = 7.0 Hz, 3H), 1.20 (d, *J* = 6.8 Hz, 6H) ppm.

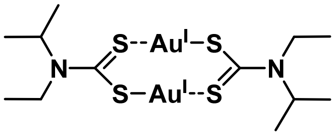

$C_{12}H_{24}Au_2N_2S_4$
M = 718.51 g/mol

[Au^I(*N*-ethyl, *N*-isopropyl)dtc]$_2$ 11: A solution of ATM (230.4 mg, 0.59 mmol, 1.0 equiv.) and a solution of lithium (*N*-ethyl, *N*-isopropyl)dithiocarbamate **S2** (150.0 mg, 0.89 mmol, 1.5 equiv.), dissolved in 10.0 mL of water each, were combined and vigorously stirred at room temperature for 2 h. The obtained orange to brown powder was filtered and thoroughly washed with water. This material was recrystallized from chloroform/hexane affording dark-orange to brown crystals as the pure product in a yield of 19% (39.8 mg, 0.06 mmol). **HRMS-ESI** (*m/z*) [Au^{III} + 2 dtc$^-$]$^+$ calcd. for $C_{12}H_{24}AuN_2S_4$: 521.0488; Found: 521.0483. **Anal.** calcd. for $C_{12}H_{24}Au_2N_2S_4$: C, 20.06; H, 3.37; N, 3.90; Found: C, 19.61; H, 3.44; N, 3.80.

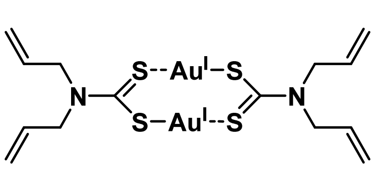

$C_{14}H_{20}Au_2N_2S_4$
M = 738.50 g/mol

[Au^I(*N*,*N*-diallyl)dtc]$_2$ 12:[6] The complex was synthesized according to the general procedure using diallyl amine (0.79 g, 1.0 mL, 8.1 mmol; 1.0 equiv.), potassium hydroxide (0.5 g, 8.93 mmol, 1.1 equiv.), carbon disulfide (0.75 g, 0.59 mL, 9.81 mmol, 1.2 equiv.) in 40.0 mL of water and ATM (0.5 g, 1.28 mmol, 1.0 equiv.) in 20.0 mL of water. A dark-yellow solid was obtained as the pure product in a yield of 71% (337.6 mg, 0.46 mmol). **^{1}H NMR** (400 MHz, $CDCl_3$) δ 5.91 (ddt, *J* = 17.2, 10.2, 5.8 Hz, 4H), 5.31 (dq, *J* = 10.2, 1.2 Hz, 4H), 5.25 (dq, *J* = 17.1, 1.4 Hz, 4H), 4.50 (dt, *J* = 5.8,

1.4 Hz, 8H) ppm. **^{13}C NMR** (100 MHz, $CDCl_3$) δ 206.2, 123.0, 119.6, 58.8 ppm. **HRMS-ESI** (*m/z*) [Au^{III} + 2 dtc^-]$^+$ calcd. for $C_{14}H_{20}AuN_2S_4$: 541.0175; Found: 541.0171. **Anal.** calcd. for $C_{14}H_{20}Au_2N_2S_4$: C, 22.77; H, 2.73; N, 3.79; Found: C, 23.25; H, 2.68; N, 4.01.

Compound S3

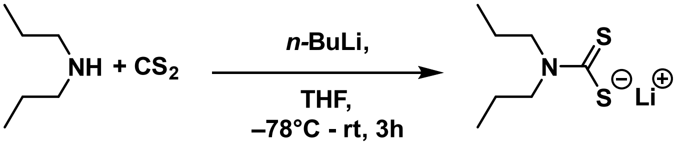

Dipropylamine (1.11 g, 1.5 mL, 10.97 mmol, 1.0 equiv.) was dissolved in 20.0 mL of dry THF and cooled to –78°C. Then *n*-butyllithium (2.5 M solution in hexane, 4.4 mL, 10.97 mmol, 1.0 equiv.) was added dropwise and the resulting mixture was stirred for 15 minutes whereupon carbon disulfide (0.84 g, 0.66 mL, 10.97 mmol, 1.0 equiv.) was added dropwise. The resulting mixture was stirred at –78°C for 15 minutes and afterwards allowed to warm up to room temperature. After a total reaction time of 3 h the solution was concentrated and dried under high vacuum over night. The product was obtained as an off-white solid in quantitative yield and was used for the next step without further purification. **^{1}H NMR** (400 MHz, D_2O) δ 3.99 – 3.90 (m, 4H), 1.79 – 1.68 (m, 4H), 0.89 (t, J = 7.5 Hz, 6H) ppm.

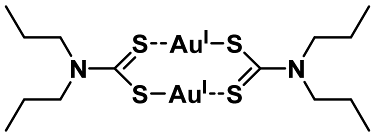

$C_{14}H_{28}Au_2N_2S_4$
M = 746.57 g/mol

[Au^I(*N*,*N*-di-*n*-propyl)dtc]$_2$ 13: A solution of ATM (478.0 mg, 1.23 mmol, 1.0 equiv.) and a solution of lithium (*N*,*N*-di-*n*-propyl) dithiocarbamate **S3** (337.1 mg, 1.84 mmol, 1.5 equiv.), dissolved in 10.0 mL of water each, were combined and vigorously stirred at room temperature over night. The obtained bright yellow powder was thoroughly washed with water, ethanol and diethyl ether successively. The crude product was obtained in a yield of 87% (400.9 mg, 0.54 mmol). Recrystallization from hot 1,2-dichloroethane afforded bright yellow needles as the pure product. **HRMS-ESI** (*m/z*) [Au^{III} + 2 dtc^-]$^+$ calcd. for $C_{14}H_{28}AuN_2S_4$: 549.0801; Found: 549.0797. **Anal.** calcd for $C_{14}H_{28}Au_2N_2S_4$: C, 22.52; H, 3.78; N, 3.75; Found: C, 22.31; H, 4.01; N, 3.64.

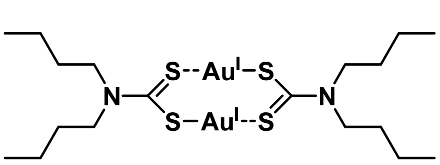

$C_{18}H_{36}Au_2N_2S_4$
M = 802.67 g/mol

[Au^I(*N*,*N*-di-*n*-butyl)dtc]$_2$ 14: A solution of ATM (407.5 mg, 1.04 mmol, 1.0 equiv.), dissolved in 10.0 mL of water, and a solution of sodium di-*n*-butyl dithiocarbamate (aqueous solution, 45 wt%; 791.7 mg, 0.73 mL, 1.57 mmol, 1.5 equiv.) were combined and vigorously stirred at room temperature over night. Afterwards, the reaction mixture was extracted with chloroform (2 x 20 mL). The combined organic phases were washed with brine (20 mL), dried over $MgSO_4$ and concentrated. The residue was recrystallized from hot 1,2-dichloroethane to

afford orange needles as the pure product in a yield of 35% (145.6 mg, 0.18 mmol). **HRMS-ESI** (*m/z*) [Au^{III} + 2 dtc^-]$^+$ calcd. for $C_{18}H_{36}AuN_2S_4$: 605.1427; Found: 605.1422. **Anal.** calcd. for $C_{18}H_{36}Au_2N_2S_4$: C, 26.93; H, 4.52; N, 3.49; Found: C, 26.83; H, 4.73; N, 3.30.

Compound S4[17]

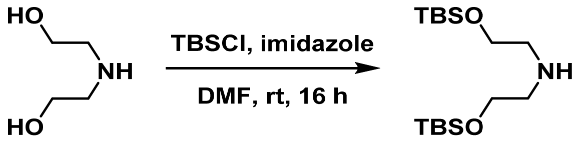

Diethanolamine (2.81 g, 26.73 mmol, 1.0 equiv.) was dissolved in 50.0 mL of dry DMF. Subsequently *tert*-butyldimethylsilyl chloride (8.06 g, 53.45 mmol, 2.0 equiv.) and imidazole (3.64 g, 53.45 mmol, 1.0 equiv.) were added. The resulting mixture was stirred at room temperature over night. The reaction mixture was diluted with 200 mL of ethyl acetate, washed with saturated $NaHCO_3$ solution (3 x 200 mL) and brine (200 mL), dried with $MgSO_4$, concentrated and dried under high vacuum over night. A white to colorless, viscous oil was obtained as the product in a yield of 96% (8.58 g, 25.71 mmol) which was used for the next step without further purification. **^{1}H NMR** (400 MHz, $CDCl_3$) δ 3.8 – 3.7 (m, 4H), 2.8 – 2.7 (m, 4H), 0.89 (s, 18H), 0.05 (s, 12H) ppm.

Compound S5

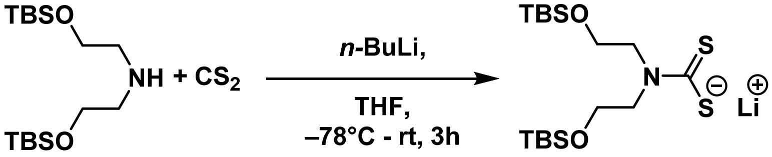

Compound **S4** (4.84 g, 14.51 mmol, 1.0 equiv.) was dissolved in 50.0 mL of dry THF and cooled to –78°C. Then *n*-butyllithium (2.5 M solution in hexane, 5.8 mL, 14.51 mmol, 1.0 equiv.) was added dropwise and the resulting mixture was stirred for 15 minutes whereupon carbon disulfide (1.1 g, 0.72 mL, 14.51 mmol, 1.0 equiv.) was added dropwise. The resulting mixture was stirred at –78°C for 15 minutes and afterwards allowed to warm up to room temperature. After a total reaction time of 3 h the solution was concentrated and dried under high vacuum over night. The product was obtained as an off-white solid in quantitative yield and was used in the next without further purification. **^{1}H NMR** (400 MHz, $CDCl_3$) δ 4.2 (t, J = 5.8 Hz, 4H), 4.0 (t, J = 5.9 Hz, 4H), 0.88 (s, 18H), 0.05 (s, 12H) ppm.

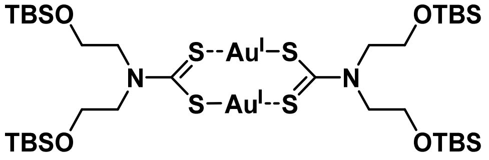

$C_{34}H_{76}Au_2N_2O_4S_4Si_4$
M = 1211.51 g/mol

[Au^{I}(*N*,*N*-*bis*(2-((*tert*-butyldimethylsilyl)oxy) ethyl) dtc]$_2$ 15: A solution of ATM (0.5 g, 1.28 mmol, 1.0 equiv) and a solution of compound **S5** (798.2 mg, 1.92 mmol, 1.5 equiv.), dissolved in 10.0 mL of water each, were combined and vigorously stirred at room temperature for 2 h. The obtained precipitate was filtered and washed with a small amount of water and dried under high vacuum over night. A grey powder was obtained as the pure product in a yield of 44% (509.7 mg, 0.42 mmol). **^{1}H NMR** (400 MHz, $CDCl_3$) δ 4.17 (t, *J* = 5.5 Hz, 8H), 4.01 (t, *J* = 5.5 Hz, 8H), 0.90 (s, 36H), 0.08 (s, 24H) ppm. **^{13}C NMR** (101 MHz, $CDCl_3$) δ 204.9, 61.3, 60.5, 26.0, 18.3, -5.2 ppm. **HRMS-ESI** (*m/z*) [Au^{III} + 2 dtc^{-}]$^{+}$ calcd. for $C_{34}H_{76}AuN_2O_4S_4Si_4$: 1013.3430; Found: 1013.3435. **Anal.** calcd. for $C_{34}H_{76}Au_2N_2O_4S_4Si_4$: C, 33.71; H, 6.32; N, 2.31; Found: C, 33.53; H, 6.39; N, 2.25.

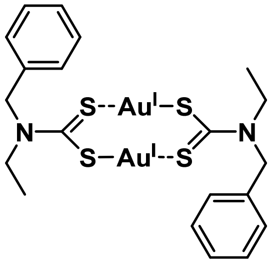

$C_{20}H_{24}Au_2N_2S_4$
M = 814.60 g/mol

[Au^{I}(*N*-benzyl, *N*-ethyl)dtc]$_2$ 16: The complex was synthesized according to the general procedure using *N*-ethylbenzylamine (124.4 mg, 0.14 mL, 0.92 mmol, 1.2 equiv.), potassium hydroxide (51.8 mg, 0.92 mmol, 1.2 equiv.), carbon disulfide (70.0 mg, 0.06 mL, 0.92 mmol, 1.2 equiv.) in 10.0 mL of water and ATM (300.0 mg, 0.77 mmol, 1.0 equiv.) in 15.0 mL of water. A bright yellow solid was obtained as the pure product in a yield of 84% (262.8 mg, 0.32 mmol). **^{1}H NMR** (400 MHz, C_6D_6) δ 7.1 – 7.0 (m, 10H), 4.7 (s, 4H), 3.4 (q, *J* = 7.1 Hz, 4H), 0.8 (t, *J* = 7.1 Hz, 6H) ppm. **HRMS** (m/z) [Au^{III} + 2 dtc^{-}]$^{+}$ calcd. for $C_{20}H_{24}AuN_2S_4$: 617.0488; Found: 617.0488. **Anal.** calcd. for $C_{20}H_{24}Au_2N_2S_4$: C, 29.49; H, 2.97; N, 3.44; Found: C, 29.51; H, 3.22; N, 3.37.

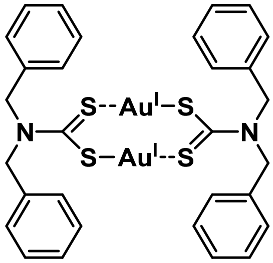

$C_{30}H_{28}Au_2N_2S_4$
M = 938.74 g/mol

[Au^{I}(*N*,*N*-dibenzyl)dtc]$_2$ 17: The complex was synthesized according to the general procedure using ATM (505.1 mg, 1.29 mmol, 1.0 equiv.) dissolved in 10.0 mL of water and sodium dibenzyl dithiocarbamate (573.7 mg, 1.94 mmol, 1.5 equiv.) dissolved in 10.0 mL of methanol. The obtained bright yellow powder was thoroughly washed with water, a small amount of ethanol and diethyl ether succesively. Recrystallization from chloroform/hexane afforded brown crystals as the pure product in a yield of 64% (390.5 mg, 0.42 mmol). **^{1}H NMR** (400 MHz, $CDCl_3$) δ 7.39 – 7.16 (m, 20H), 5.07 (s, 8H) ppm. **^{13}C NMR** (100 MHz, $CDCl_3$) δ 207.7, 134.6, 129.0, 128.1, 127.8, 58.7 ppm. **Anal.** calcd. for $C_{30}H_{28}Au_2N_2S_4$: C, 38.38; H, 3.01; N, 2.98; Found: C, 38.31; H, 3.28; N, 2.86.

Compound S6

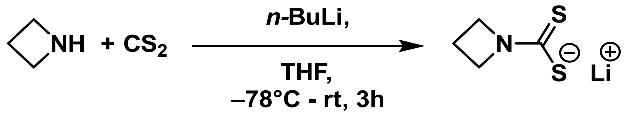

Azetidine (0.85 g, 1.0 mL, 14.89 mmol, 1.0 equiv.) was dissolved in 50.0 mL of dry THF and cooled to –78°C. Then *n*-butyllithium (2.5 M solution in hexane, 6.0 mL, 14.89 mmol, 1.0 equiv.) was added dropwise and the resulting mixture was stirred for 15 minutes whereupon carbon disulfide (1.13 g, 0.9 mL, 14.89 mmol, 1.0 equiv.) was added dropwise. The resulting mixture was stirred at –78°C for 15 minutes and afterwards allowed to warm up to room temperature. After a total reaction time of 3 h the solution was concentrated and dried under high vacuum over night. The product was obtained as a white solid in quantitative yield and was used for the next step without further purification. **^{1}H NMR** (400 MHz, D_2O) δ 4.19 – 4.13 (m, 4H), 2.18 – 2.09 (m, 2H) ppm.

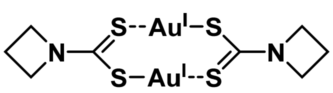

$C_8H_{12}Au_2N_2S_4$
M = 658.37 g/mol

[Au^I(azetidinyl)dtc]$_2$ 18: A solution of ATM (646.0 mg, 1.66 mmol, 1.0 equiv.) and a solution of lithium azetidinyl dithiocarbamate **S6** (345.7 mg, 2.48 mmol, 1.5 equiv.), dissolved in 10.0 mL of water each, were combined and vigorously stirred at room temperature for 2 h. The obtained bright yellow powder was filtered and thoroughly washed with water, a small amount of ethanol and diethyl ether successively and dried under high vacuum over night. The product was obtained in a yield of 78% (427.9 mg, 0.65 mmol). **Anal.** calcd. for $C_8H_{12}Au_2N_2S_4$: C, 14.59; H, 1.84; N, 4.25; Found: C, 14.68; H, 2.24; N, 4.05.

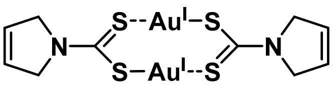

$C_{10}H_{12}Au_2N_2S_4$
M = 682.39 g/mol

[Au^I(3-pyrrolinyl)dtc]$_2$ 19: The complex was synthesized according to the general procedure using 3-pyrroline (0.18 g, 0.2 mL, 2.6 mmol, 1.2 equiv.), carbon disulfide (0.2 g, 0.16 mL, 2.6 mmol, 1.2 equiv.), potassium hydroxide (146.1 mg, 2.6 mmol, 1.2 equiv.) in 10.0 mL of water and ATM (845.2 mg, 2.17 mmol, 1.0 equiv.) in 15.0 mL of water. A shiny red solid was obtained as the pure product in a yield of 48% (355.2 mg, 0.517 mmol). **Anal.** calcd. for $C_{10}H_{12}Au_2N_2S_4$: C, 17.60; H, 1.77; N, 4.11; Found: C, 17.75; H, 2.06; N, 3.85.

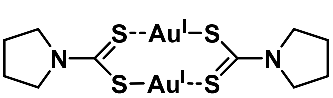

$C_{10}H_{16}Au_2N_2S_4$
M = 686.43 g/mol

[Au^I(pyrrolidinyl)dtc]$_2$ 20:[18] A solution of ATM (400.0 mg, 1.03 mmol, 1.0 equiv.) and a solution of ammonium pyrrolidine dithiocarbamate (336.8 mg, 2.05 mmol, 2.0 equiv.), dissolved in 5.0 mL of water each, were combined and vigorously stirred at room temperature over night. The obtained orange powder was centrifuged. The supernatant was taken away and the residue was resuspended in water and again centrifuged (procedure repeated twice). The obtained orange solid was dried under high vacuum over night and afterwards recrystallized form hot DMF to

afford orange-colored needles as the pure product in a yield of 38% (133.8 mg, 0.20 mmol). **Anal.** calcd. for $C_{10}H_{16}Au_2N_2S_4$: C, 17.50; H, 2.35; N, 4.08; Found: C, 17.73; H, 2.53; N, 4.23.

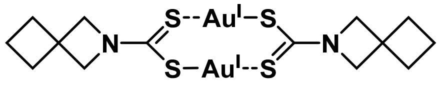

$C_{14}H_{20}Au_2N_2S_4$
M = 738.50 g/mol

[Au^{I}(2-azaspiro[3.3]heptanyl)dtc]$_2$ 21: The complex was synthesized according to the general procedure using 2-azaspiro[3.3]heptane hydrochloride (122.9 mg, 0.92 mmol, 1.2 equiv.), potassium hydroxide (103.6 mg, 1.84 mmol, 2.4 equiv.), carbon disulfide (70.0 mg, 0.06 mL, 0.92 mmol, 1.2 equiv.) in 10.0 mL of water and ATM (300.0 mg, 0.77 mmol, 1.0 equiv.) in 15.0 mL of water. A pale yellow solid was obtained as the pure product in a yield of 72% (203.6 mg, 0.28 mmol). **HRMS-ESI** (*m/z*) [Au^{III} + 2 dtc$^-$]$^+$ calcd. for $C_{14}H_{20}AuN_2S_4$: 541.0175; Found: 541.0176. **Anal.** calcd. for $C_{14}H_{20}Au_2N_2S_4$: C, 22.77; H, 2.73; N, 3.79; Found: C, 23.05; H, 2.86; N, 3.89.

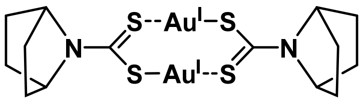

$C_{14}H_{20}Au_2N_2S_4$
M = 738.50 g/mol

[Au^{I}(7-azabicyclo[2.2.1]heptanyl)dtc]$_2$ 22: The complex was synthesized according to the general procedure using 7-azabicyclo[2.2.1]heptane hydrochloride (123.0 mg, 0.92 mmol, 1.2 equiv.), potassium hydroxide (103.2 mg, 1.84 mmol, 2.4 equiv.), carbon disulfide (70.0 mg, 0.06 mL, 0.92 mmol, 1.2 equiv.) in 10.0 mL of water and ATM (300.0 mg, 0.77 mmol, 1.0 equiv.) in 15.0 mL of water. A bright red solid was obtained as the pure product in a yield of 42% (124.6 mg, 0.16 mmol). **HRMS-ESI** (*m/z*) [Au^{III} + 2 dtc$^-$]$^+$ calcd. for $C_{14}H_{20}AuN_2S_4$: 541.0175; Found: 541.0173. **Anal.** calcd. for $C_{14}H_{20}Au_2N_2S_4 + H_2O$: C, 22.23; H, 2.93; N, 3.70; Found: C, 22.17; H, 2.91; N, 3.64.

Compound S7

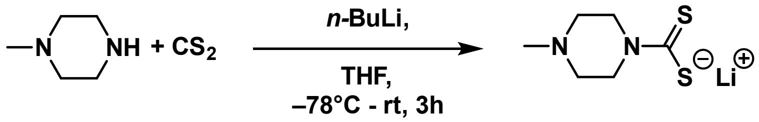

N-methylpiperidine (1.35 g, 1.5 mL, 13.48 mmol, 1.0 equiv.) was dissolved in 20.0 mL of dry THF and cooled to –78°C. Then *n*-butyllithium (2.5 M solution in hexane, 5.4 mL, 13.48 mmol, 1.0 equiv.) was added dropwise and the resulting mixture was stirred for 15 minutes whereupon carbon disulfide (1.03 g, 0.81 mL, 13.48 mmol, 1.0 equiv.) was added dropwise. The resulting mixture was stirred at –78°C for 15 minutes and afterwards allowed to warm up to room temperature. After a total reaction time of 3 h the solution was concentrated and dried under high vacuum over night. The product was obtained as an off-white solid in quantitative yield and was used for the next step without further purification. **^{1}H NMR** (400 MHz, D_2O) δ 4.35 (br. s, 4H), 2.53 (br. s, 4H), 2.29 (s, 3H) ppm.

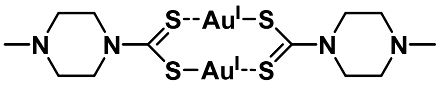

$C_{12}H_{22}Au_2N_4S_4$
M = 744.51 g/mol

[Au^{I}(4-methylpiperazinyl)dtc]$_2$ 23: A Solution of ATM (502.0 mg, 1.29 mmol, 1.0 equiv.) and a solution of lithium 4-methylpiperazinyl dithiocarbamate **S7** (351.7 mg, 1.93 mmol, 1.5 equiv.), dissolved in 10.0 mL of water each, were combined and vigorously stirred at room temperature for 2 h. The obtained bright yellow powder was filtered and thoroughly washed with water, a small amount of ethanol and diethyl ether succesively. The crude product was obtained in a yield of 86% (414.8 mg, 0.56 mmol). Recrystallization from hot 1,2-dichloroethane afforded very fine, bright yellow needles as the pure product. **HRMS-ESI** (*m/z*) [Au^{III} + 2 dtc^{-}]$^{+}$ calcd. for $C_{12}H_{22}AuN_4S_4$: 547.0393; Found: 547.0380. **Anal.** calcd. for $C_{12}H_{22}Au_2N_4S_4$: C, 19.36; H, 2.98; N, 7.53; Found: C, 19.42; H, 3.07; N, 7.85.

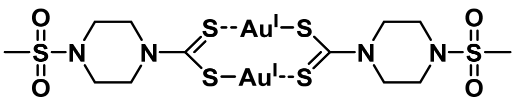

$C_{12}H_{22}Au_2N_4O_4S_6$
M = 872.63 g/mol

[Au^{I}(4-methylsulfonylpiperazinyl)dtc]$_2$ 24: The complex was synthesized according to the general procedure using 4-methylsulfonylpiperazin (315.3 mg, 1.92 mmol, 1.5 equiv.), potassium hydroxide (118.5 mg, 2.11 mmol, 1.6 equiv.), carbon disulfide (0.15 g, 0.12 mL, 1.92 mmol, 1.5 equiv.) in 20.0 mL of water and ATM (0.5 g, 1.28 mmol, 1.0 equiv.) in 20.0 mL of water. A pale-brown solid was obtained as the pure product in a yield of 69% (385.9 mg, 0.442 mmol). **HRMS-ESI** (*m/z*) [Au^{III} + 2 dtc^{-}]$^{+}$ calcd. for $C_{12}H_{22}AuN_4O_4S_6$: 674.9631; Found: 674.9627. **Anal.** calcd. for $C_{12}H_{22}Au_2N_4O_4S_6$: C, 16.52; H, 2.54; N, 6.42; Found: C, 16.49; H, 2.59; N, 6.51.

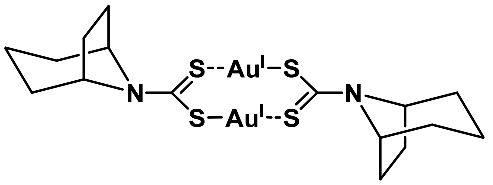

$C_{16}H_{24}Au_2N_2S_4$
M = 766.56 g/mol

[Au^{I}(8-azabicyclo[3.2.1]octanyl)dtc]$_2$ 25: The complex was synthesized according to the general procedure using 8-azabicyclo[3.2.1]octane hydrochloride (135.8 mg, 0.92 mmol, 1.2 equiv.), potassium hydroxide (103.6 mg, 1.84 mmol, 2.4 equiv.), carbon disulfide (70.0 mg, 0.06 mL, 0.92 mmol, 1.2 equiv.) in 10.0 mL of water and ATM (300.0 mg, 0.77 mmol, 1.0 equiv.) in 15.0 mL of water. A pale yellow solid was obtained as the pure product in a yield of 83% (247.0 mg, 0.32 mmol). **HRMS-ESI** (*m/z*) [Au^{III} + 2 dtc^{-}]$^{+}$ calcd. for $C_{16}H_{24}AuN_2S_4$: 569.0488; Found: 569.0486. **Anal.** calcd. for $C_{16}H_{24}Au_2N_2S_4$: C, 25.07; H, 3.16; N, 3.65; Found: C, 25.22; H, 3.12; N, 3.70.

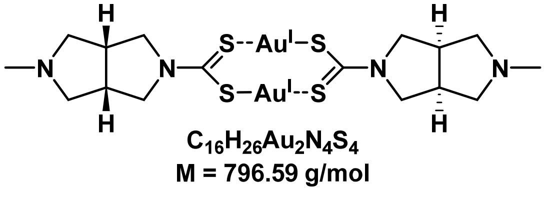

$C_{16}H_{26}Au_2N_4S_4$
M = 796.59 g/mol

[Au^{I}((*3aR, 6aS*)-2-methyl octahydro pyrrolo[3,4C] pyrrolidinyl)dtc]$_2$ 26: The complex was synthesized according to the general procedure using (*3aR, 6aS*)-2-methyl octahydropyrrolo[3, 4C]pyrrole (116.1 mg, 0.92 mmol, 1.2 equiv.), potassium hydroxide (51.8 mg, 0.92 mmol, 1.2 equiv.), carbon disulfide (70.0 mg, 0.06 mL, 0.92 mmol, 1.2 equiv.)

in 10.0 mL of water and ATM (300.0 mg, 0.77 mmol, 1.0 equiv.) in 15.0 mL of water. A bright yellow solid was obtained as the pure product in a yield of 57% (174.5 mg, 0.22 mmol). **HRMS-ESI** (*m/z*) $[M]^+$ calcd. for $C_{16}H_{27}Au_2N_4S_4$: 797.0444, Found: 797.0452; (*m/z*) $[Au^{III} + 2\ dtc^-]^+$ calcd. for $C_{16}H_{26}AuN_4S_4$: 599.0706; Found: 599.0706. **Anal.** calcd. for $C_{16}H_{26}Au_2N_4S_4$: C, 24.12; H, 3.29; N, 7.03; Found: C, 23.80; H, 3.20; N, 7.00.

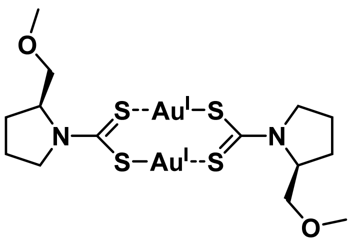

$C_{14}H_{24}Au_2N_2O_2S_4$
M = 774.53 g/mol

[Au^I((*S*)-(+)-2-(methoxymethyl)pyrrolidinyl)dtc]$_2$ 27: The complex was synthesized according to the general procedure using (*S*)-(+)-2-(methoxymethyl)pyrrolidine (106.0 mg, 0.11 mL, 0.92 mmol, 1.2 equiv.), potassium hydroxide (51.8 mg, 0.92 mmol, 1.2 equiv.), carbon disulfide (70.0 mg, 0.06 mL, 0.92 mmol, 1.2 equiv.) in 10.0 mL of water and ATM (300.0 mg, 0.77 mmol, 1.0 equiv.) in 15.0 mL of water. A bright yellow solid was obtained as the pure product in a yield of 52% (156.0 mg, 0.20 mmol). **HRMS-ESI** (*m/z*) $[Au^{III} + 2\ dtc^-]^+$ calcd. for $C_{14}H_{24}AuN_2O_2S_4$: 577.0391; Found: 577.0393. **Anal.** calcd. for $C_{14}H_{24}Au_2N_2O_2S_4$: C, 21.71; H, 3.12; N, 3.62; Found: C, 21.60; H, 3.26; N, 3.49.

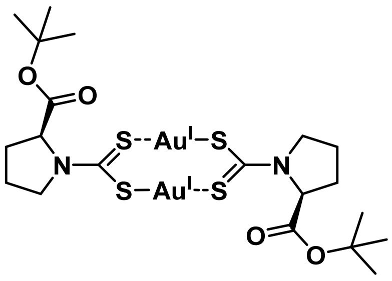

$C_{20}H_{32}Au_2N_2O_4S_4$
M = 886.66 g/mol

[Au^I(*tert*-butyl (*L*)-prolinyl)dtc]$_2$ 28: The complex was synthesized according to the general procedure using (*L*)-proline *tert*-butyl ester (200.0 mg,1.17 mmol, 1.2 equiv.), potassium hydroxide (71.8 mg, 1.28 mmol, 1.1 equiv.), carbon disulfide (101.3 mg, 0.08 mL, 1.33 mmol, ~1.4 equiv.) in 5.0 mL of water and ATM (380.0 mg, 0.98 mmol, 1.0 equiv) in 10.0 mL of water. A yellow solid was obtained as the pure product in a yield of 89% (385.7 mg, 0.435 mmol). **HRMS-ESI** (*m/z*) $[Au^{III} + 2\ dtc^-]^+$ calcd. for $C_{20}H_{32}AuN_2O_4S_4$: 689.0910; Found: 689.0907. **Anal.** calcd. for $C_{20}H_{32}Au_2N_2O_4S_4$: C, 27.09; H, 3.64; N, 3.16; Found: C, 27.17; H, 3.84; N, 3.06.

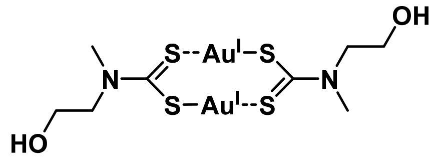

$C_8H_{16}Au_2N_2O_2S_4$
M = 694.4011 g/mol

[Au^I(*N*-2-hydroxyethyl, *N*-methyl)dtc]$_2$ 29: The complex was synthesized according to the general procedure using 2-methylaminoethanol (86.6 mg, 0.09 mL, 1.15 mmol, 1.5 equiv.), potassium hydroxide (64.5 mg, 1.15 equiv.), carbon disulfide (76.0 mg, 0.06 mL, 1.0 equiv.) in 10.0 mL of water and ATM (300.0 mg, 0.77 mmol, 1.0 equiv.) in 15.0 mL of water. A greenish solid was obtained which was recrystallized from hot ethanol to afford small orange-red needles as the pure product in a yield of 73% (196.5 mg, 0.28 mmol). **^{1}H NMR** (400 MHz, DMSO-*d6*) δ 5.04 (t, *J* = 5.5 Hz, 2H), 4.02 (t, *J* = 5.7 Hz, 4H), 3.74 (q, *J* = 5.6 Hz, 4H), 3.49 (s, 6H) ppm. **^{13}C NMR** (100 MHz, DMSO-*d6*) δ 201.4, 61.4, 57.9, 47.0 ppm. **HRMS-ESI** (*m/z*)

$[Au^{III} + 2\ dtc^-]^+$ calcd. for $C_8H_{16}AuN_2O_2S_4$: 496.9760; Found: 496.9757. **Anal.** calcd. for $C_8H_{16}Au_2N_2O_2S_4$: C, 13.84; H, 2.32; N, 4.03; Found: C, 13.76; H, 2.43; N, 3.98.

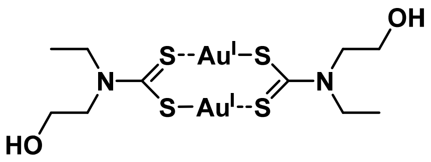

$C_{10}H_{20}Au_2N_2O_2S_4$
M = 722.46 g/mol

[Au^I(*N*-ethyl, *N*-2-hydroxyethyl)dtc]$_2$ 30: The complex was synthesized according to the general procedure using 2-ethylaminoethanol (82.0 mg, 0.09 mL, 0.92 mmol, 1.2 equiv.), potassium hydroxide (51.8 mg, 0.92 mmol, 1.2 equiv.), carbon disulfide (70.0 mg, 0.06 mL, 0.92 mmol, 1.2 equiv.) in 10.0 mL of water and ATM (300.0 mg, 0.77 mmol, 1.0 equiv.) in 15.0 mL of water. A bright red solid was obtained as the pure product in a yield of 44% (123.5 mg, 0.17 mmol). **^{1}H NMR** (400 MHz, DMSO-*d*$_6$) δ 5.06 (t, *J* = 5.4 Hz, 2H), 3.99 (q, *J* = 7.2 Hz, 4H), 3.95 (t, *J* = 6.0 Hz, 4H), 3.77 (q, *J* = 5.7 Hz, 4H), 1.27 (t, *J* = 7.0 Hz, 6H) ppm. **^{13}C NMR** (100 MHz, DMSO-*d*$_6$) δ 201.0, 58.7, 57.8, 53.8, 11.2 ppm. **HRMS-ESI** (*m/z*) $[Au^{III} + 2\ dtc^-]^+$ calcd. for $C_{10}H_{20}AuN_2O_2S_4$: 525.0073; Found: 525.0080. **Anal.** calcd. for $C_{10}H_{20}Au_2N_2O_2S_4 + H_2O$: C, 16.22; H, 2.99; N, 3.78; Found: C, 16.15; H, 2.83; N, 3.64.

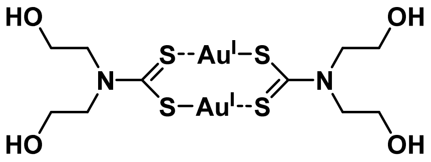

$C_{10}H_{20}Au_2N_2O_4S_4$
M = 754.4531 g/mol

[Au^I(*N*,*N*-di-2-hydroxyethyl)dtc]$_2$ 31: The complex was synthesized according to the general procedure using diethanolamine (96.7 mg, 0.92 mmol, 1.2 equiv.), potassium hydroxide (51.8 mg, 0.92 mmol, 1.2 equiv.), carbon disulfide (70.0 mg, 0.06 mL, 0.92 mmol, 1.2 equiv.) in 10.0 mL of water and ATM (300.0 mg, 0.77 mmol, 1.0 equiv.) in 15.0 mL of water. A gold-colored solid was obtained as the pure product in a yield of 34% (98.5 mg, 0.13 mmol). **^{1}H NMR** (400 MHz, DMSO-*d*$_6$) δ 5.06 (t, *J* = 5.3 Hz, 4H), 4.06 (t, *J* = 5.8 Hz, 8H), 3.78 (dt, *J* = 5.6 Hz, 8H) ppm. **^{13}C NMR** (100 MHz, DMSO-*d*$_6$) δ 201.7, 60.8, 57.7 ppm. **HRMS-ESI** (*m/z*) $[Au^{III} + 2\ dtc^-]^+$ calcd. for $C_{10}H_{20}AuN_2O_4S_4$: 556.9971; Found: 556.9977. **Anal.** calcd. for $C_{10}H_{20}Au_2N_2O_4S_4$: C, 15.92; H, 2.67; N, 3.71; Found: C, 15.62; H, 2.88; N, 3.61.

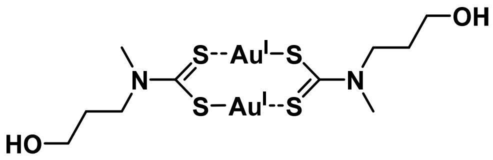

$C_{10}H_{20}Au_2N_2O_2S_4$
M = 722.46 g/mol

[Au^I(*N*-3-hydroxyethyl, *N*-methyl)dtc]$_2$ 32: The complex was synthesized according to the general procedure using 3-methylamino-1-propanol (82.0 mg, 0.08 mL, 0.92 mmol, 1.2 equiv.), potassium hydroxide (51.8 mg, 0.92 mmol, 1.2 equiv.), carbon disulfide (70.0 mg, 0.06 mL, 0.92 mmol, 1.2 equiv.) in 10.0 mL of water and ATM (300.0 mg, 0.77 mmol, 1.0 equiv.) in 15.0 mL of water. A greenish to brown solid was obtained as the pure product in a yield of 76% (210.9 mg, 0.29 mmol). **^{1}H NMR** (400 MHz, DMSO-*d*$_6$) δ 4.63 (t, *J* = 5.0 Hz, 2H), 4.01 – 3.92 (m, 4H), 3.49 – 3.43 (m, 10H), 1.93 – 1.81 (m, 4H) ppm. **^{13}C NMR** (100 MHz, DMSO-*d*$_6$) δ 200.9, 58.1, 57.0, 45.7, 29.5 ppm. **HRMS-ESI** (m/z) $[Au^{III} + 2\ dtc^-]^+$ calcd. for $C_{10}H_{20}AuN_2O_2S_4$: 525.0073; Found: 525.0070. **Anal.** calcd. for $C_{10}H_{20}Au_2N_2O_2S_4$: C, 16.62; H, 2.79; N, 3.88; Found: C, 16.38; H, 2.88; N, 3.87.

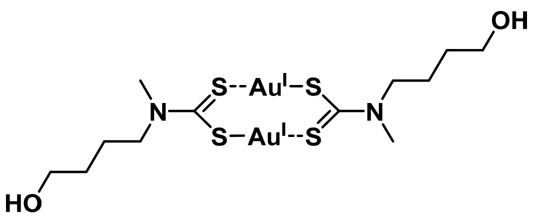

$C_{12}H_{24}Au_2N_2O_2S_4$
M = 750.51 g/mol

[Au^{I}(*N*-4-hydroxybutyl, *N*-methyl)dtc]$_2$ 33: The complex was synthesized according to the general procedure using 4-(methylamino)-1-butanol (94.9 mg, 0.92 mmol, 1.2 equiv.), potassium hydroxide (51.8 mg, 0.92 mmol, 1.2 equiv.), carbon disulfide (70.0 mg, 0.06 mL, 0.92 mmol, 1.2 equiv.) in 10.0 mL of water and ATM (300.0 mg, 0.77 mmol, 1.0 equiv.) in 15.0 mL of water. A yellow to orange-colored solid was obtained as the pure product in a yield of 42% (123.7 mg, 0.16 mmol). **^{1}H NMR** (400 MHz, DMSO-*d*$_6$) δ 4.48 (t, *J* = 5.1 Hz, 2H), 3.93 (dd, *J* = 9.0, 6.2 Hz, 4H), 3.47 – 3.38 (m, 10H), 1.82 – 1.68 (m, 4H), 1.43 (p, *J* = 6.7 Hz, 4H) ppm. **^{13}C NMR** (101 MHz, DMSO-*d*$_6$) δ 200.9, 60.3, 59.2, 45.3, 29.5, 23.1 ppm. **HRMS-ESI** (*m/z*) [Au^{III} + 2 dtc^{-}]$^{+}$ calcd. for $C_{12}H_{24}AuN_2O_2S_4$: 553.0386; Found: 553.0389. **Anal.** calcd. for $C_{12}H_{24}Au_2N_2O_2S_4$: C, 19.20; H, 3.22; N, 3.73; Found: C, 19.07; H, 3.37; N, 3.65.

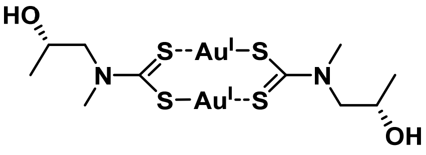

$C_{10}H_{20}Au_2N_2O_2S_4$
M = 722.47 g/mol

[Au^{I}(*N*-2-(*S*)-hydroxypropyl, *N*-methyl)dtc]$_2$ 34: The complex was synthesized according to the general procedure using (2*S*)-1-(methylamino)propan-2-ol hydrochloride (115.0 mg, 0.92 mmol, 1.2 equiv.), potassium hydroxide (103.6 mg, 1.84 mmol, 2.4 equiv.), carbon disulfide (70.0 mg, 0.06 mL, 0.92 mmol, 1.2 equiv.) in 10.0 mL of water and ATM (300.0 mg, 0.77 mmol, 1.0 equiv.) in 15.0 mL of water. A gold-colored solid was obtained as the pure product in a yield of 47% (129.6 mg, 0.18 mmol). **^{1}H NMR** (400 MHz, DMSO-*d*$_6$) δ 5.06 (d, *J* = 5.3 Hz, 2H), 4.25 – 4.05 (m, 4H), 3.62 (dd, *J* = 13.5, 8.5 Hz, 2H), 3.50 (s, 6H), 1.08 (d, *J* = 6.3 Hz, 6H) ppm. **^{13}C NMR** (100 MHz, DMSO-*d*$_6$) δ 201.6, 66.1, 64.4, 47.6, 21.2 ppm. **HRMS-ESI** (*m/z*) [Au^{III} + 2 dtc^{-}]$^{+}$ calcd. for $C_{10}H_{20}AuN_2O_2S_4$: 525.0073; Found: 525.0072. **Anal.** calcd. for $C_{10}H_{20}Au_2N_2O_2S_4$: C, 16.62; H, 2.79; N, 3.88; Found: C, 16.35; H, 2.85; N, 3.93.

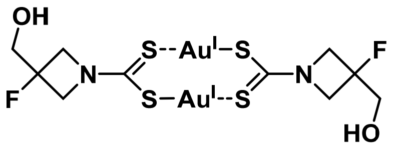

$C_{10}H_{14}Au_2F_2N_2O_2S_4$
M = 754.4039 g/mol

[Au^{I}(3-fluoro-, 3-hydroxymethylazetidinyl)dtc]$_2$ 35: The complex was synthesized according to the general procedure using (3-fluoroazetidin-3-yl)methanol (96.7 mg, 0.92 mmol, 1.2 equiv.), carbon disulfide (70.0 mg, 0.92 mmol, 1.2 equiv.) and potassium hydroxide (51.8 mg, 0.92 mmol, 1.2 equiv.) in 10.0 mL of water and ATM (300.0 mg, 0.77 mmol, 1.0 equiv.) in 15.0 mL of water. This complex did not immediately precipitate from the reaction mixture as for most compounds. Therefor the reaction mixture was allowed to stand at room temperature for 2 days, whereupon a white powder precipitated. The solid was filtered through a sintered glass frit (pore size 4), washed with 10 mL of water and dried under high vacuum. The pure product was obtained as an off-white solid (which upon dissolution in DMSO turns yellow) in a yield of 36% (105.2 mg, 0.139 mmol). **^{1}H NMR** (400 MHz, DMSO-*d*$_6$) δ 5.4 (t, *J* = 6.1 Hz, 2H), 4.5 – 4.3 (m, 8H), 3.7

(dd, J = 19.5, 5.2 Hz, 4H) ppm. **^{13}C NMR** (101 MHz, DMSO-d_6) δ 200.2 (d, J = 3.2 Hz), 89.4 (d, J = 208.2 Hz), 64.6 (d, J = 28.2 Hz), 61.7 (d, J = 26.6 Hz) ppm. **HRMS-ESI** (*m/z*) $[M + Na]^+$ calcd. for $C_{10}H_{14}Au_2F_2N_2NaO_2S_4$: 776.9130; Found: 776.9136. **Anal.** calcd. for $C_{10}H_{14}Au_2F_2N_2O_2S_4$: C, 15.92; H, 1.87; N, 3.71; Found: C, 15.80; H, 1.98; N, 3.78.

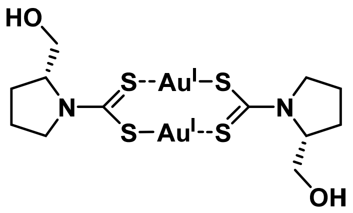

$C_{12}H_{20}Au_2N_2O_2S_4$
M = 746.48 g/mol

[Au^I((*R*)-prolinoyl)dtc]$_2$ 36: The complex was synthesized according to the general procedure using (*R*)-(–)-prolinol (93.1 mg, 0.92 mmol, 1.2 equiv.), potassium hydroxide (51.8 mg, 0.92 mmol, 1.2 equiv.), carbon disulfide (70.0 mg, 0.06 mL, 0.92 mmol, 1.2 equiv.) in 10.0 mL of water and ATM (300.0 mg, 0.77 mmol, 1.0 equiv.) in 15.0 mL of water. A yellow to gold-colored solid was obtained as the pure product in a yield of 53% (150.6 mg, 0.20 mmol). **^{1}H NMR** (400 MHz, DMSO-d_6) δ 5.06 (t, J = 5.7 Hz, 2H), 4.53 – 4.46 (m, 2H), 3.81 – 3.73 (m, 4H), 3.74 – 3.52 (m, 4H), 2.22 – 1.94 (m, 8H) ppm. **^{13}C NMR** (100 MHz, DMSO-d_6) δ 198.2, 68.3, 59.0, 56.6, 27.7, 23.1 ppm. **HRMS-ESI** (*m/z*) $[Au^{III} + 2\ dtc^-]^+$ calcd. for $C_{12}H_{20}AuN_2O_2S_4$: 549.0073; Found: 549.0082. **Anal.** calcd. for $C_{12}H_{20}Au_2N_2O_2S_4$: C, 19.31; H, 2.70; N, 3.75; Found: C, 19.30; H, 3.00; N, 3.75.

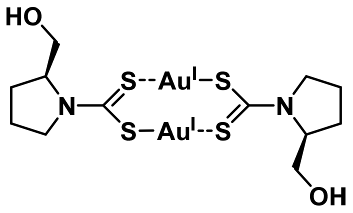

$C_{12}H_{20}Au_2N_2O_2S_4$
M = 746.48 g/mol

[Au^I((*S*)-prolinoyl)dtc]$_2$ 37: The complex was synthesized according to the general procedure using (*S*)-(+)-prolinol (93.1 mg, 0.92 mmol, 1.2 equiv.), potassium hydroxide (51.8 mg, 0.92 mmol, 1.2 equiv.), carbon disulfide (70.0 mg, 0.06 mL, 0.92 mmol, 1.2 equiv.) in 10.0 mL of water and ATM (300.0 mg, 0.77 mmol, 1.0 equiv.) in 15.0 mL of water. A pale-yellow solid was obtained which was recrystallized from hot ethanol (with a few drops of DMF) to afford red-brownish crystals as the pure product in a yield of 82% (236.8 mg, 0.32 mmol). **^{1}H NMR** (400 MHz, DMSO-d_6) δ 5.11 – 5.01 (m, 2H), 4.54 – 4.44 (m, 2H), 3.81 – 3.74 (m, 4H), 3.74 – 3.52 (m, 4H), 2.24 – 1.93 (m, 8H) ppm. **^{13}C NMR** (100 MHz, DMSO-d_6) δ 198.2, 68.2, 59.0, 56.6, 27.7, 23.1 ppm. **HRMS-ESI** (*m/z*) $[Au^{III} + 2\ dtc^-]^+$ calcd. for $C_{12}H_{20}AuN_2O_2S_4$: 549.0073; Found: 549.0070. **Anal.** calcd. for $C_{12}H_{20}Au_2N_2O_2S_4$: C, 19.31; H, 2.70; N, 3.75; Found: C, 19.17; H, 3.00; N, 3.68.

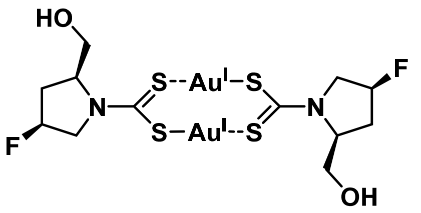

$C_{12}H_{18}Au_2F_2N_2O_2S_4$
M = 782.47 g/mol

[Au^I(4-(*S*)-fluoro-(*S*)-prolinoyl)dtc] 38: The complex was synthesized according to the general procedure using ((2*S*, 4*S*)-4-fluoropyrrolidin-2-yl)methanol hydrochloride (143.2 mg, 0.92 mmol, 1.2 equiv.), potassium hydroxide (103.6 mg, 1.84 mmol, 2.4 equiv.), carbon disulfide (70.0 mg, 0.06 mL, 0.92 mmol, 1.2 equiv.) in 10.0 mL of water and ATM (300.0 mg, 0.77 mmol, 1.0 equiv.) in 15.0 mL of water. A gold-colored solid was obtained as the pure product in a yield of 64% (192.5 mg, 0.246 mmol).

^{1}H NMR (400 MHz, DMSO-*d*$_6$) δ 5.4 (dt, *J* = 53.9, 4.3 Hz, 2H), 5.1 (t, *J* = 5.8 Hz, 2H), 4.6 (td, *J* = 9.5, 8.2, 4.1 Hz, 2H), 4.2 (ddd, *J* = 35.5, 15.4, 4.6 Hz, 2H), 4.1 (dt, *J* = 9.0, 4.1 Hz, 2H), 4.0 (dd, *J* = 25.5, 15.4 Hz, 2H), 2.5 – 2.3 (m, 6H) ppm. **^{13}C NMR** (101 MHz, DMSO-*d*$_6$) δ 200.6, 93.1 (d, *J* = 174.4 Hz), 67.4, 62.7 (d, *J* = 24.6 Hz), 59.0 (d, *J* = 4.3 Hz), 33.7 (d, *J* = 19.0 Hz) ppm. **HRMS-ESI** (*m/z*) [Au^{III} + 2 dtc$^-$]$^+$ calcd. for $C_{12}H_{18}AuF_2N_2O_2S_4$: 584.9885; Found: 584.9894. **Anal.** calcd. for $C_{12}H_{18}Au_2F_2N_2O_2S_4$ + H_2O: C, 18.01; H, 2.52; N, 3.50; Found: C, 18.22; H, 2.66; N, 3.63.

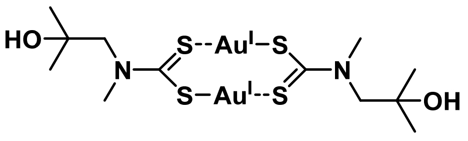

$C_{12}H_{24}Au_2N_2O_2S_4$
M = 750.52 g/mol

[Au^I(*N*-2-hydroxy, 2-methylpropyl, *N*-methyl)dtc]$_2$ 39: The complex was synthesized according to the general procedure using 2-methyl-1-(methylamino)propan-2-ol (94.9 mg, 0.92 mmol, 1.2 equiv.), potassium hydroxide (51.8 mg, 0.92 mmol, 1.2 equiv.), carbon disulfide (70.0 mg, 0.06 mL, 0.92 mmol, 1.2 equiv.) in 10.0 mL of water and ATM (300.0 mg, 0.77 mmol, 1.0 equiv.) in 15.0 mL of water. A gold-colored solid was obtained as the pure product in a yield of 47% (134.5 mg, 0.18 mmol). **^{1}H NMR** (400 MHz, DMSO-*d*$_6$) δ 4.84 (s, 2H), 4.03 (s, 4H), 3.53 (s, 6H), 1.18 (s, 12H) ppm. **^{13}C NMR** (101 MHz, DMSO-*d*$_6$) δ 202.8, 71.3, 67.8, 47.2, 27.8 ppm. **HRMS-ESI** (*m/z*) [Au^{III} + 2 dtc$^-$]$^+$ calcd. for $C_{12}H_{24}AuN_2O_2S_4$: 553.0386; Found: 553.0382. **Anal.** calcd. for $C_{12}H_{24}Au_2N_2O_2S_4$ + H_2O: C, 18.75; H, 3.41; N, 3.65; Found: C, 18.73; H, 3.39; N, 3.61.

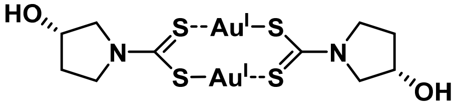

$C_{10}H_{16}Au_2N_2O_2S_4$
M = 718.42 g/mol

[Au^I((*S*)-3-hydroxypyrrolidinyl)dtc]$_2$ 40: The complex was synthesized according to the general procedure using (*S*)-(+)-3-pyrrolidinol (80.2 mg, 0.92 mmol, 1.2 equiv.), potassium hydroxide (51.8 mg, 0.92 mmol, 1.2 equiv.), carbon disulfide (70.0 mg, 0.06 mL, 0.92 mmol, 1.2 equiv.) in 10.0 mL of water and ATM (300.0 mg, 0.77 mmol, 1.0 equiv.) in 15.0 mL of water. A bright yellow solid was obtained as the pure product in a yield of 61% (167.6 mg, 0.23 mmol). **HRMS-ESI** (*m/z*) [Au^{III} + 2 dtc$^-$]$^+$ calcd. for $C_{10}H_{16}AuN_2O_2S_4$: 520.9760; Found: 520.9777. **Anal.** calcd. for $C_{10}H_{16}Au_2N_2O_2S_4$ + H_2O: C, 16.31; H, 2.46; N, 3.80; Found: C, 16.21; H, 2.46; N, 3.71.

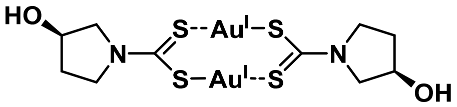

$C_{10}H_{16}Au_2N_2O_2S_4$
M = 718.42 g/mol

[Au^I((*R*)-3-hydroxypyrrolidinyl)dtc]$_2$ 41: The complex was synthesized according to the general procedure using (*R*)-(–)-3-pyrrolidinol (80.2 mg, 0.92 mmol, 1.2 equiv.), potassium hydroxide (51.8 mg, 0.92 mmol, 1.2 equiv.), carbon disulfide (70.0 mg, 0.06 mL, 0.92 mmol, 1.2 equiv.) in 10.0 mL of water and ATM (300.0 mg, 0.77 mmol, 1.0 equiv.) in 15.0 mL of water. A bright yellow solid was obtained as the pure product in a yield of 65% (179.2 mg, 0.25 mmol). **HRMS-ESI** (*m/z*) [Au^{III} + 2 dtc$^-$]$^+$ calcd. for $C_{10}H_{16}AuN_2O_2S_4$: 520.9760; Found: 520.9760. **Anal.** calcd. for $C_{10}H_{16}Au_2N_2O_2S_4$: C, 16.72; H, 2.24; N, 3.90; Found: C, 16.43; H, 2.40; N, 3.86.

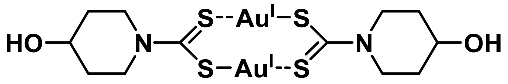

$C_{12}H_{20}Au_2N_2O_2S_4$
M = 746.48 g/mol

[Au^{I}(4-hydroxypiperidinyl)dtc]$_2$ 42: The complex was synthesized according to the general procedure using 4-hydroxypiperidine (101.2 mg, 1.0 mmol, 1.2 equiv.), potassium hydroxide (61.7 mg, 1.1 mmol, 1.2 equiv.), carbon disulfide (63.2 mg, 0.05 mL, 0.83 mmol, 1.0 equiv.) in 5.0 mL of water and ATM (325.1 mg, 0.83 mmol, 1.0 equiv.) in 10.0 mL of water. A bright-yellow powder was obtained as the pure product in a yield of 60% (225.4 mg, 0.30 mmol). **HRMS-ESI** (*m/z*) [Au^{III} + 2 dtc^{-}]$^{+}$ calcd. for $C_{12}H_{20}AuN_2O_2S_4$: 549.0073; Found: 549.0074. **Anal.** calcd. for $C_{12}H_{20}Au_2N_2O_2S_4$: C, 19.31; H, 2.70; N, 3.75; Found: C, 19.49; H, 2.65; N, 3.94.

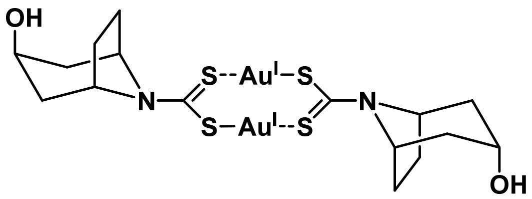

$C_{16}H_{24}Au_2N_2O_2S_4$
M = 798.55 g/mol

[Au^{I}(tropinyl)dtc]$_2$ 43: The complex was synthesized according to the general procedure using nortropin hydrochloride (148.7 mg, 0.92 mmol, 1.2 equiv.), potassium hydroxide (103.6 mg, 1.84 mmol, 2.4 equiv.), carbon disulfide (70.0 mg, 0.06 mL, 0.92 mmol, 1.2 equiv.) in 10.0 mL of water and ATM (300.0 mg, 0.77 mmol, 1.0 equiv.) in 15.0 mL of water. A pale yellow solid was obtained as the pure product in a yield of 83% (254.2 mg, 0.32 mmol). **HRMS-ESI** (*m/z*) [Au^{III} + 2 dtc^{-}]$^{+}$ calcd. for $C_{16}H_{24}AuN_2O_2S_4$: 601.0386; Found: 601.0386. **Anal.** calcd. for $C_{16}H_{24}Au_2N_2O_2S_4$: C, 24.06; H, 3.03; N, 3.51; Found: C, 23.89; H, 3.31; N, 3.42.

References

[1] M. V. Rekharsky, Y. Inoue, *Chem. Rev.* **1998**, *98*, 1875-1918.

[2] *The experimental structures are depicted using Mercury 4.2.0 (http://www.ccdc.cam.ac.uk/mercury/).*

[3] D. Paliwoda, P. Wawrzyniak, A. Katrusiak, *J. Phys. Chem. Lett.* **2014**, *5*, 2182-2188.

[4] S. Y. Ho, E. R. T. Tiekink, *Z. Kristallogr. NCS* **2002**, *217*, 589-590.

[5] R. Hesse, P. Jennische, *Acta Chem. Scand.* **1972**, *26*, 3855-3864.

[6] S. Naeem, S. A. Serapian, A. Toscani, A. J. P. White, G. Horgarth, J. D. E. T. Wilton-Ely, *Inorg. Chem.* **2014**, *53*, 2404-2416.

[7] M. A. Mansour, W. B. Connick, R. J. Lachicotte, H. J. Gysling, R. Eisenberg, *J. Am. Chem. Soc.* **1998**, *120*, 1329-1330.

[8] P. Bishop, P. Marsh, A. K. Brisdon, B. J. Brisdon, M. F. Mahon, *J. Chem. Soc., Dalton Trans.* **1998**, 675-682.

[9] B.-C. Tzeng, A. Chao, *Chem. Eur. J.* **2015**, *21*, 2083-2089.

[10] A. C. Lane, C. L. Barnes, M. V. Vollmer, J. R. Walensky, *Inorganics* **2014**, *2*, 540-551.

[11] S. Han, O.-S. Jung, Y.-A. Lee, *Transition Met. Chem.* **2011**, *36*, 691-697.

[12] B.-C. Tzeng, W.-H. Liu, J.-H. Liao, G.-H. Lee, S.-M. Peng, *Cryst. Growth Des.* **2004**, *4*, 573-577.

[13] A. P. Langaro, A. K. R. Souza, C. Y. Morassuti, S. M. Lima, G. A. Casagrande, V. M. Deflon, L. A. O. Nunes, L. H. Da Cunha Andrade, *J. Phys. Chem. A* **2016**, *120*, 9249-9256.

[14] J. Arias, M. Bardají, P. Espinet, *Inorg. Chem.* **2008**, *47*, 1597-1606.

[15] a) E. Amtmann, N. Gunkel, A. K. Miller, M. Morgen,"Compositions Comprising a Metal Source, Dithiocarbamate and Cyclodextrin", WO 2018/069525, **2018**; b) E. Amtmann, N. Gunkel, A. K. Miller, M. Morgen, "Improved Dithiocarbamate Based Compounds, Therapies and Diagnostics", WO 2020/161337, **2020**.

[16] T. Masaaki, O. Koichiro, M. Seijiro, *Chem. Lett.* **2005**, *34*, 192-193.

[17] S. Chen, N. J. S. Huby, N. Kong, J. A. Moliterni, O. J. Morales, "Substituted Hydantoins", WO 2009/021887, **2009**.

[18] F. Baril-Robert, M. A. Radtke, C. Reber, *J. Phys. Chem. C* **2012**, *116*, 2192-2197.

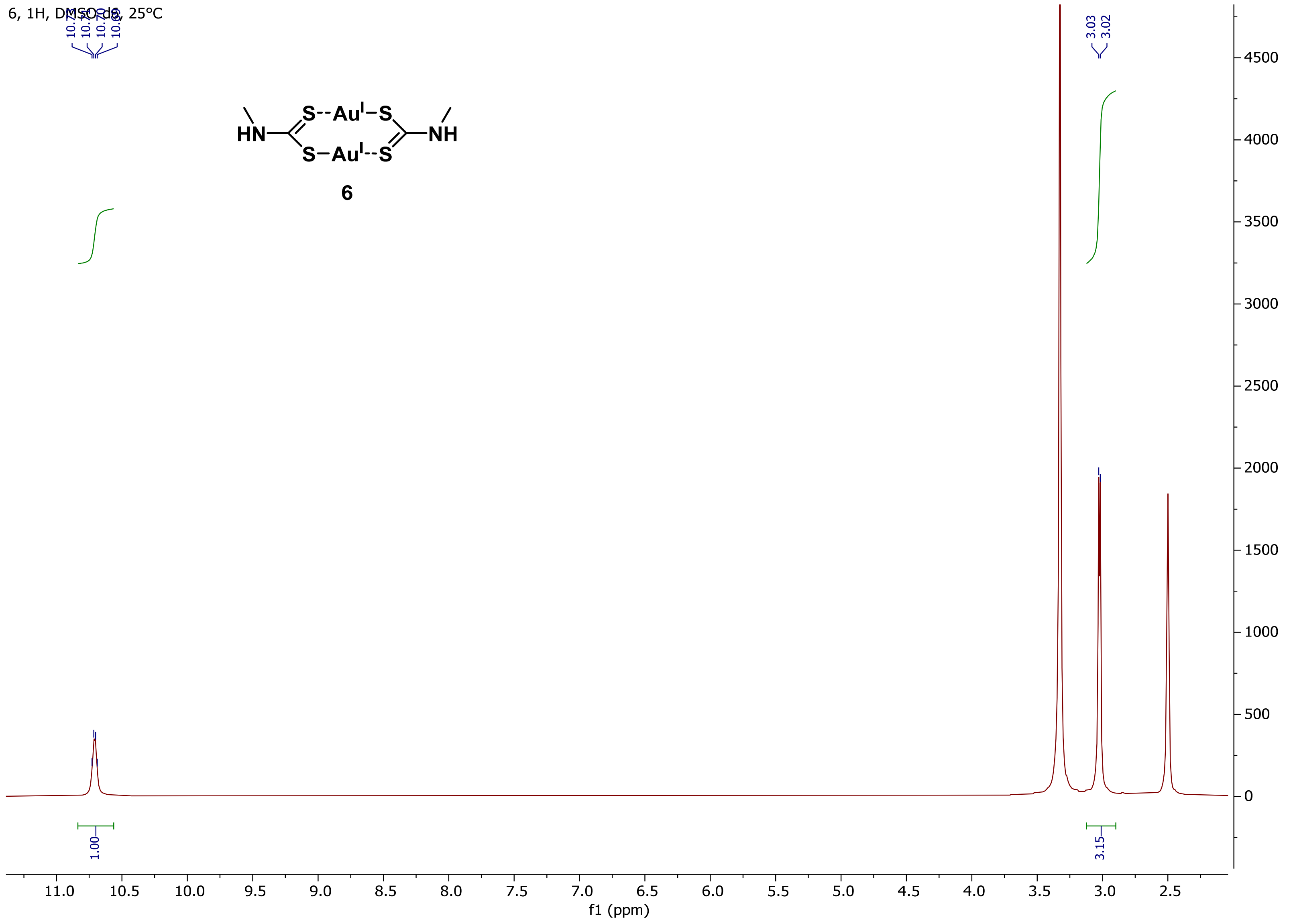
6, 1H, DMSO-d6, 25°C
3.03
3.02
HN
NH
S
Au^I
6
1.00
3.15
11.0
10.5
10.0
9.5
9.0
8.5
8.0
7.5
7.0
6.5
6.0
5.5
5.0
4.5
4.0
3.5
3.0
2.5
f1 (ppm)
4500
4000
3500
3000
2500
2000
1500
1000
500
0

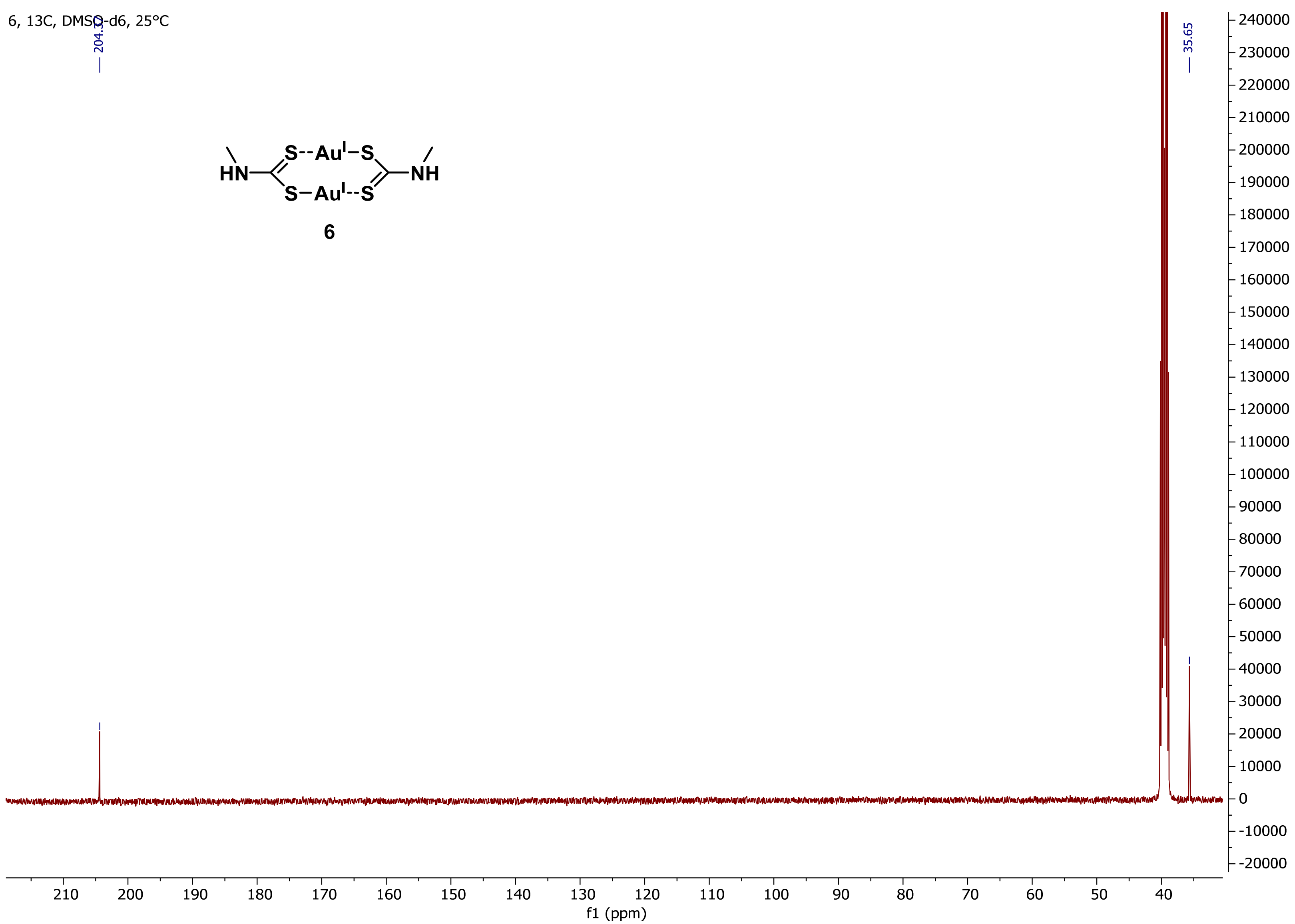
6, 13C, DMSO-d6, 25°C
35.65
HN
NH
S
Au
6
f1 (ppm)

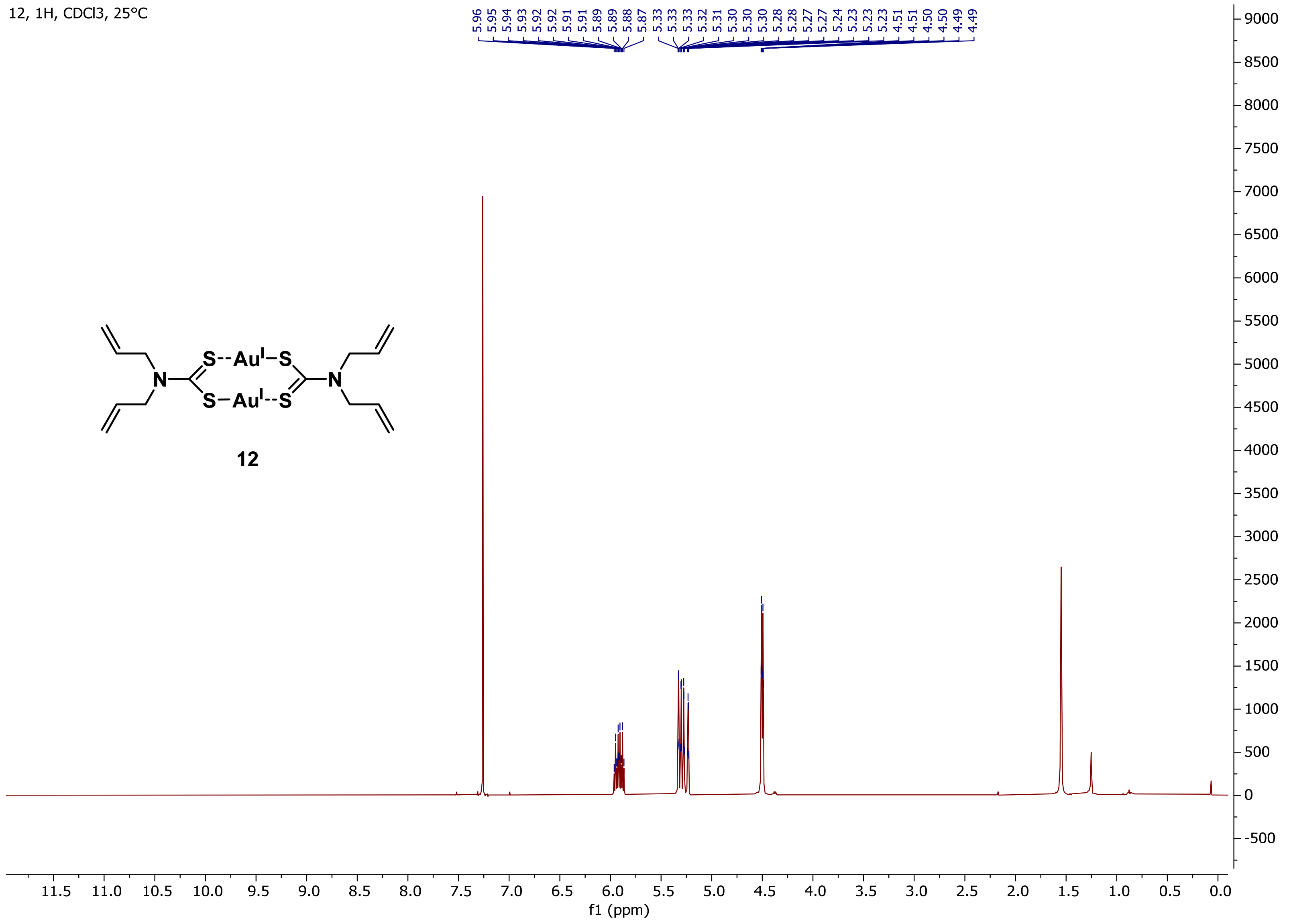
12, 1H, CDCl3, 25°C
5.96 5.95 5.94 5.93 5.92 5.92 5.91 5.91 5.89 5.89 5.88 5.87 5.33 5.33 5.33 5.32 5.31 5.30 5.30 5.30 5.28 5.28 5.27 5.27 5.24 5.23 5.23 5.23 4.51 4.51 4.50 4.49 4.49
12
f1 (ppm)

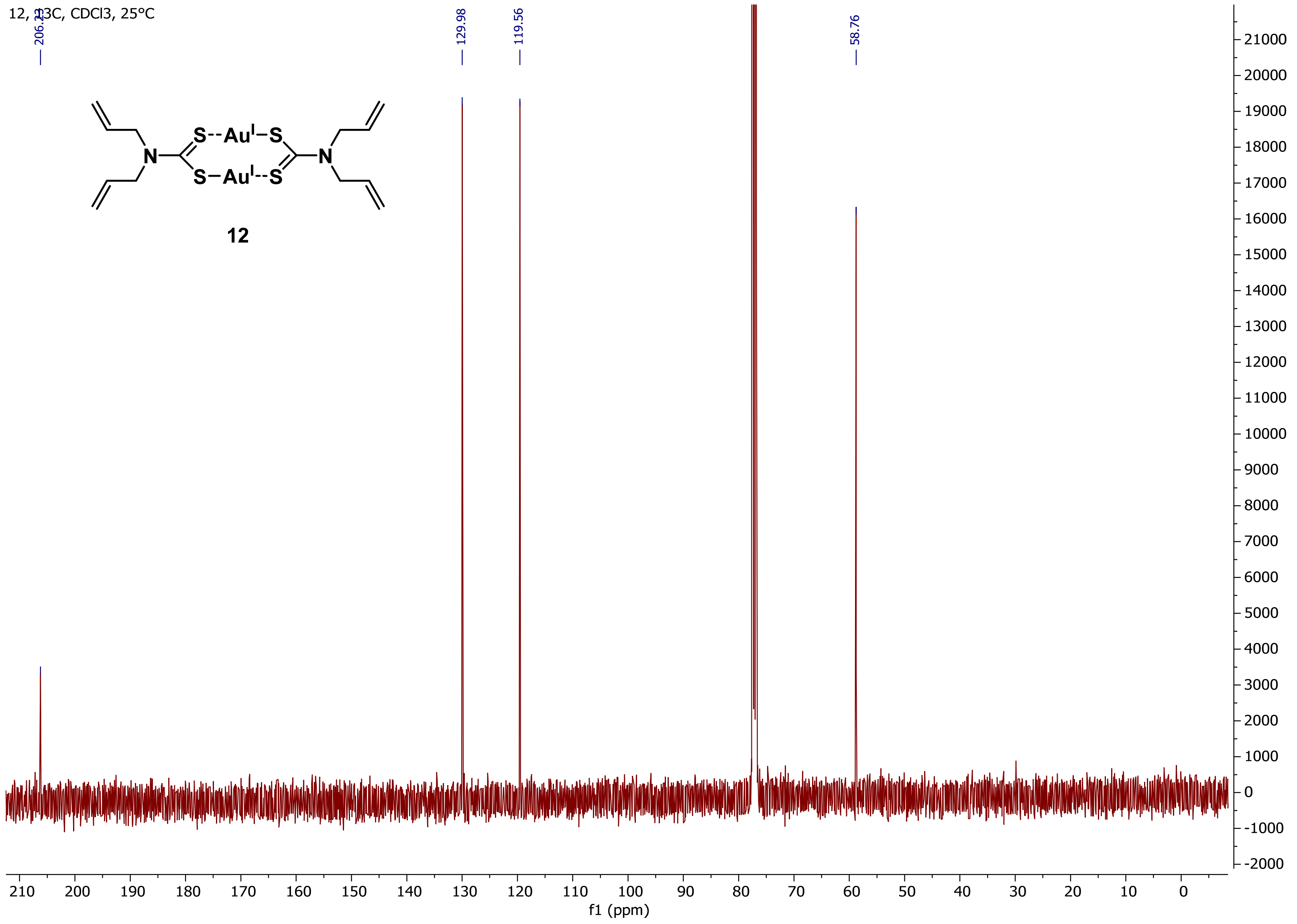
12, 13C, CDCl3, 25°C
206.23
129.98
119.56
58.76
S--Au^I–S
N
S–Au^I--S
12
21000
20000
19000
18000
17000
16000
15000
14000
13000
12000
11000
10000
9000
8000
7000
6000
5000
4000
3000
2000
1000
0
-1000
-2000
210 200 190 180 170 160 150 140 130 120 110 100 90 80 70 60 50 40 30 20 10 0
f1 (ppm)

15, 1H, CDCl3, 25°C

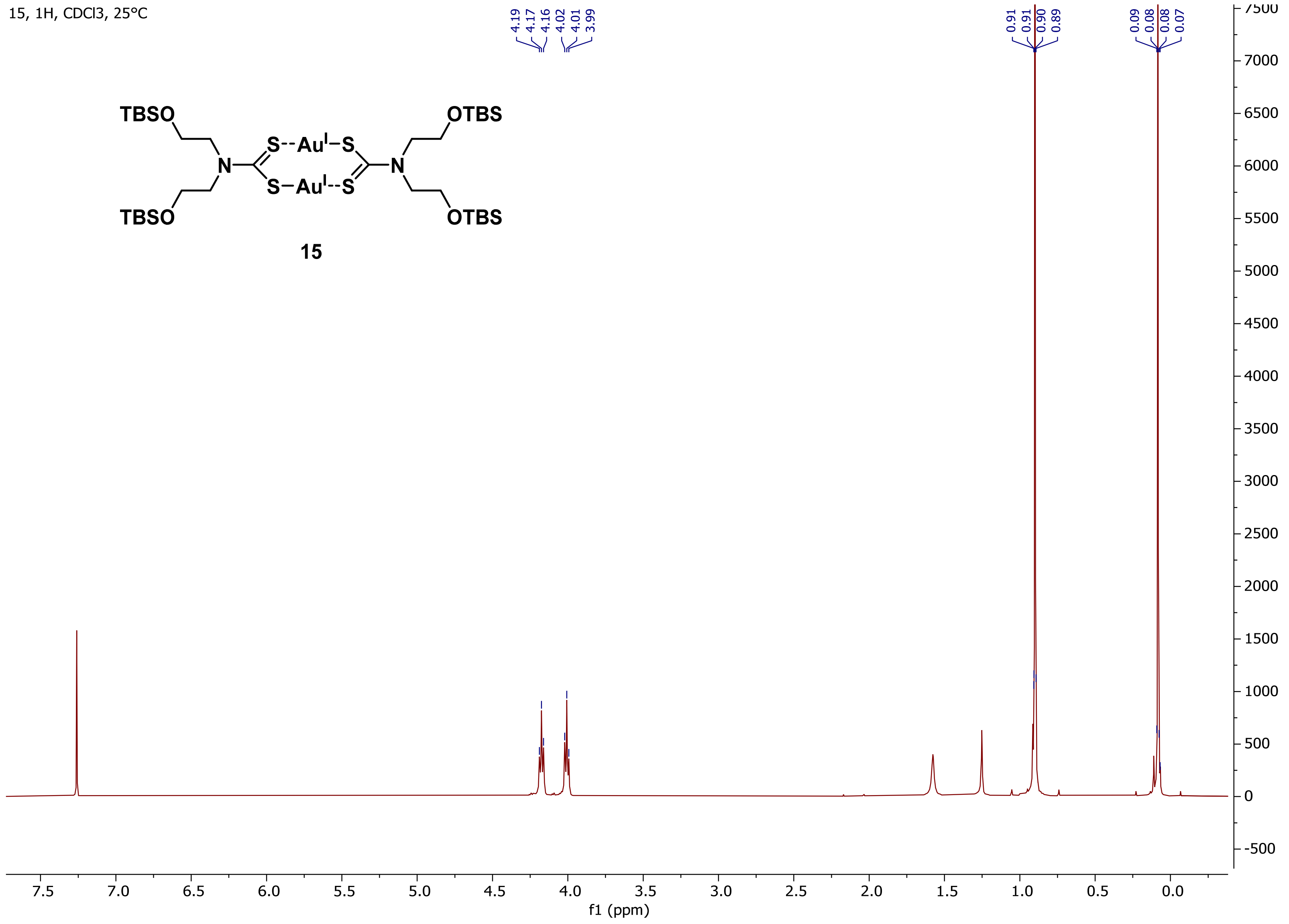

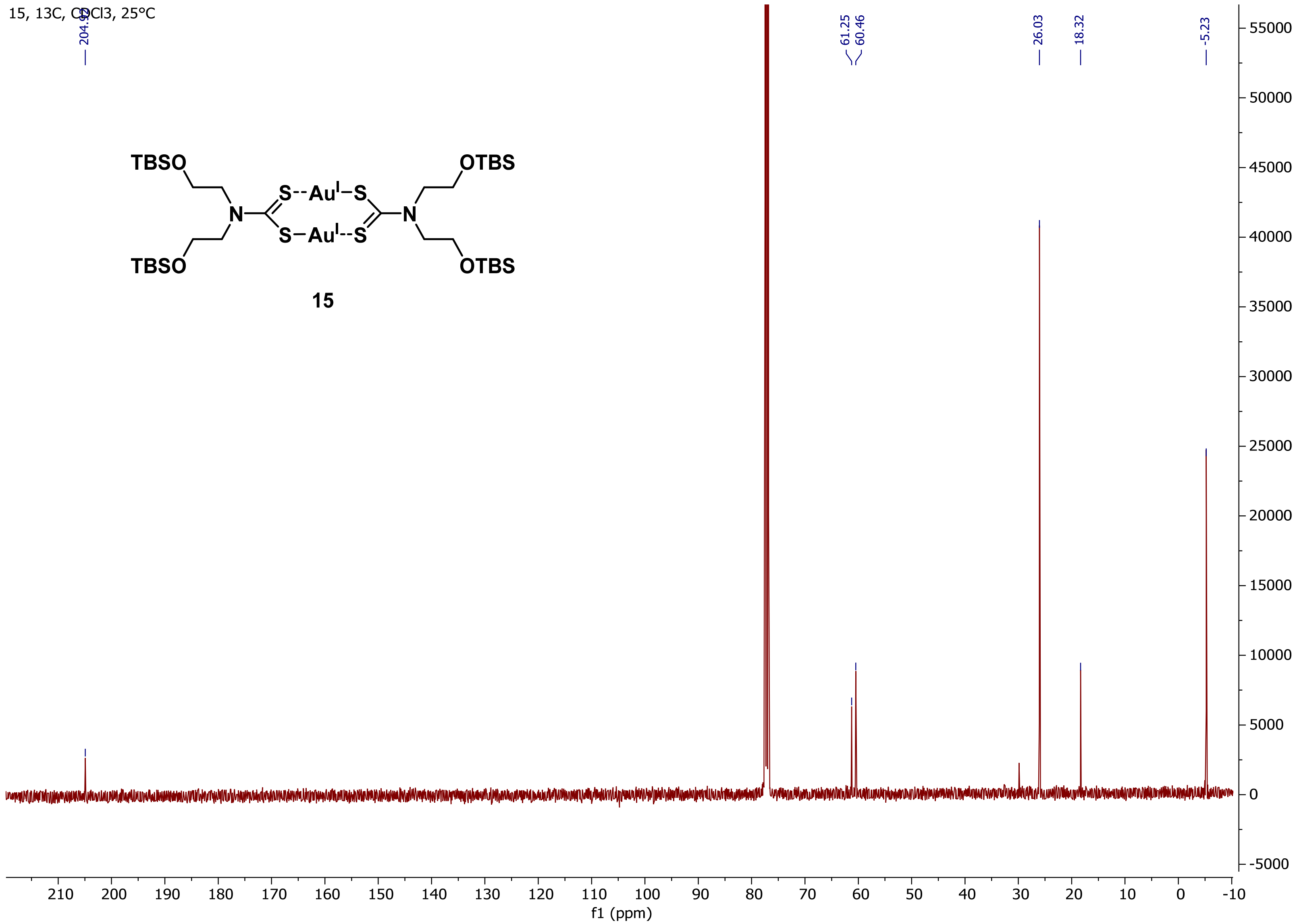
15, 13C, CDCl3, 25°C
204.92
61.25
60.46
26.03
18.32
-5.23
TBSO
OTBS
N
S
Au
15
f1 (ppm)

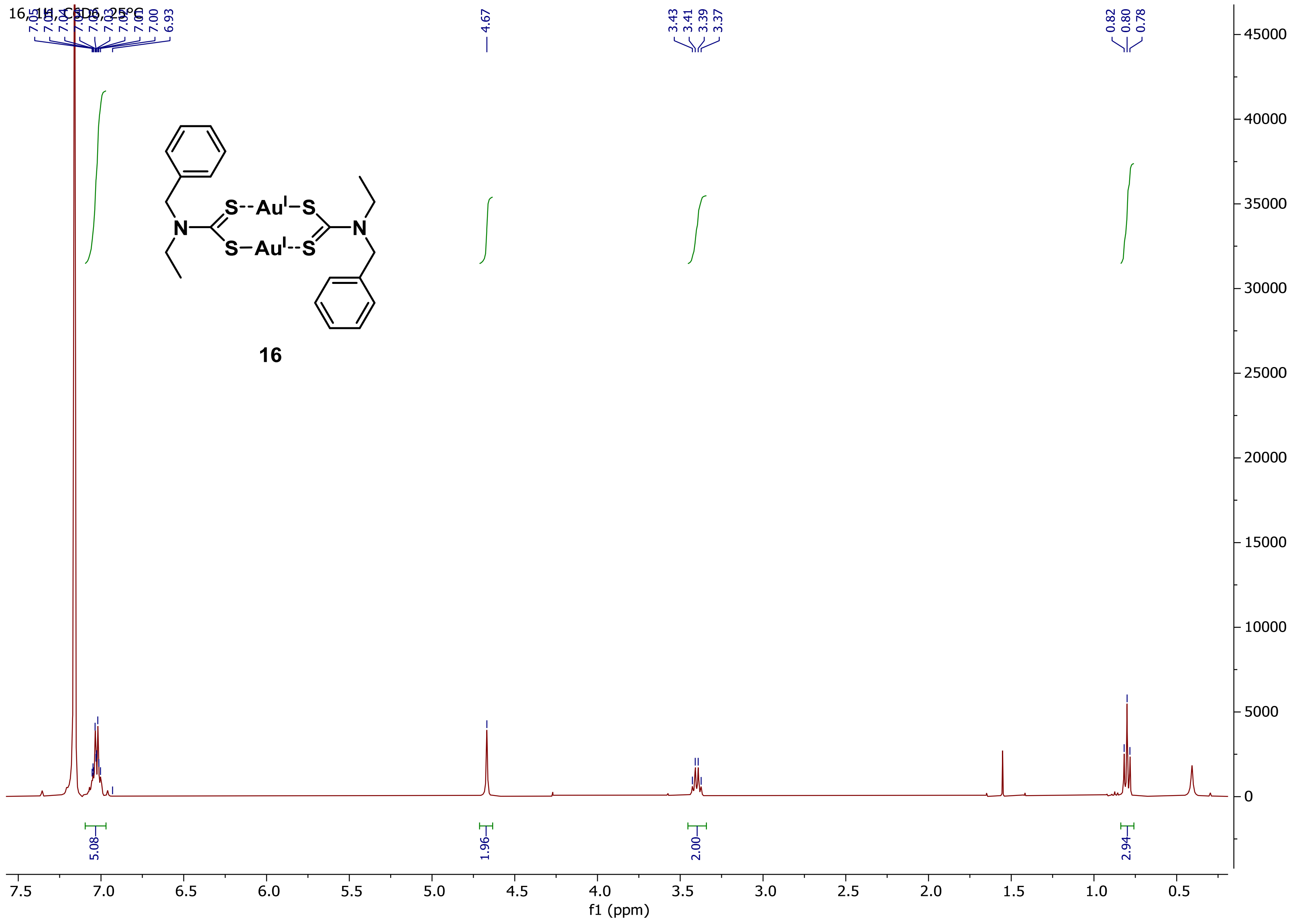
16_1H_C6D6_25°C
7.05
7.04
7.04
7.03
7.03
7.02
7.02
7.01
7.00
6.93
4.67
3.43
3.41
3.39
3.37
0.82
0.80
0.78
S
Au
N
16
5.08
1.96
2.00
2.94
7.5
7.0
6.5
6.0
5.5
5.0
4.5
4.0
3.5
3.0
2.5
2.0
1.5
1.0
0.5
f1 (ppm)
45000
40000
35000
30000
25000
20000
15000
10000
5000
0

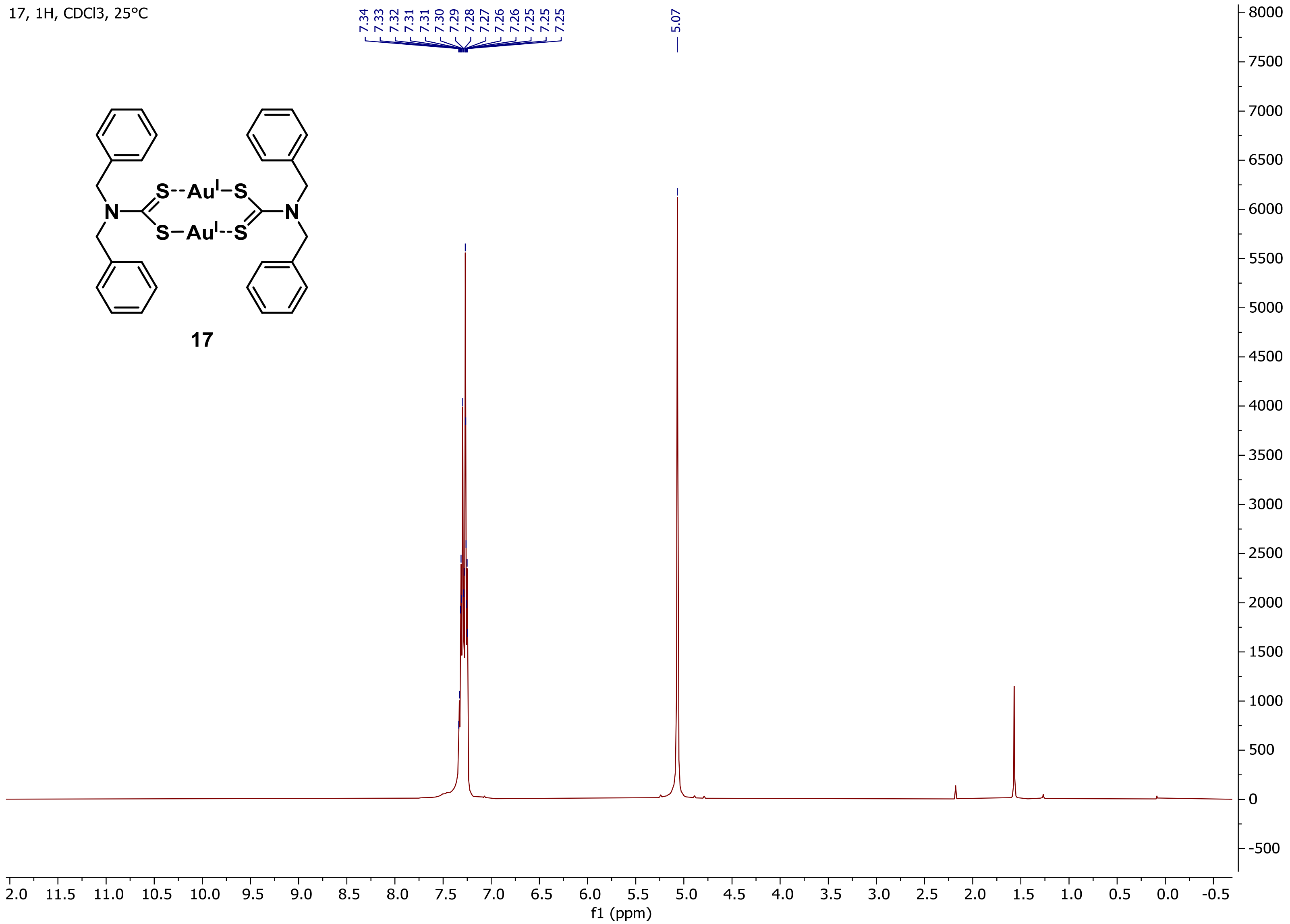
17, 1H, CDCl3, 25°C
7.34
7.33
7.32
7.31
7.31
7.30
7.29
7.28
7.27
7.26
7.26
7.25
7.25
7.25
5.07
S
Au
I
N
17
8000
7500
7000
6500
6000
5500
5000
4500
4000
3500
3000
2500
2000
1500
1000
500
0
-500
2.0 11.5 11.0 10.5 10.0 9.5 9.0 8.5 8.0 7.5 7.0 6.5 6.0 5.5 5.0 4.5 4.0 3.5 3.0 2.5 2.0 1.5 1.0 0.5 0.0 -0.5
f1 (ppm)

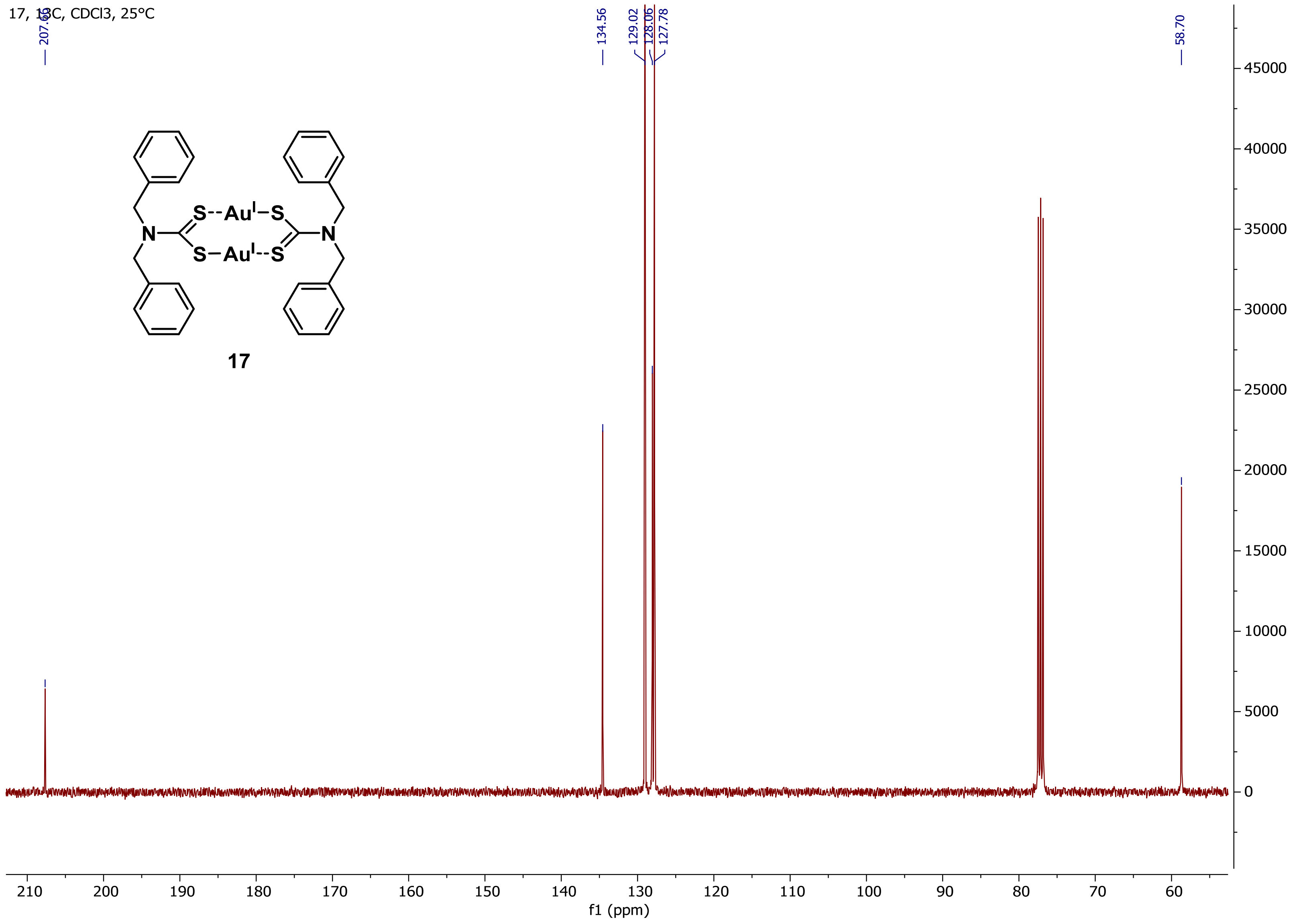
17, 13C, CDCl3, 25°C
207.68
134.56
129.02
128.06
127.78
58.70
S
Au
N
17
f1 (ppm)

29, 1H, DMSO-d6, 25°C

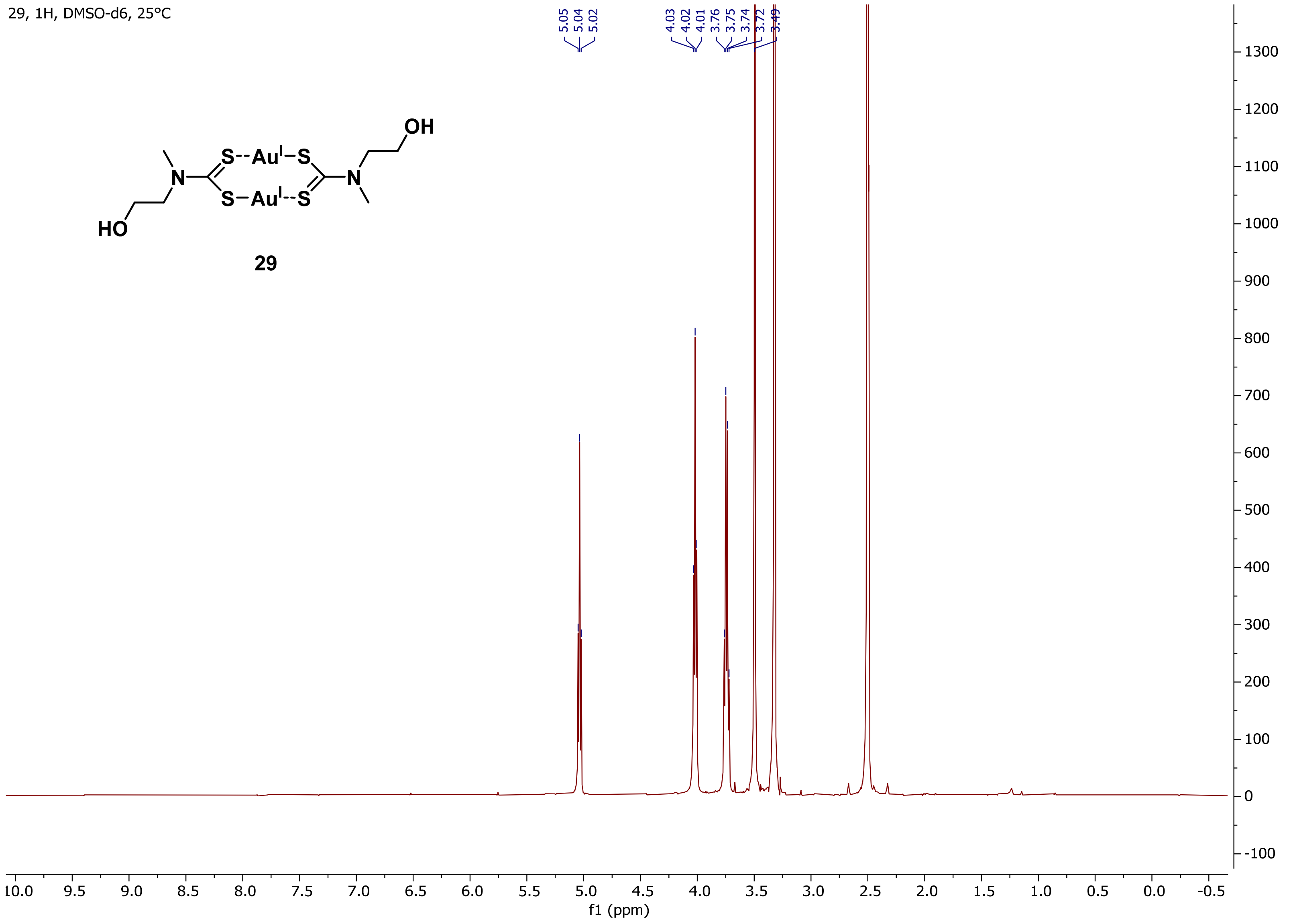

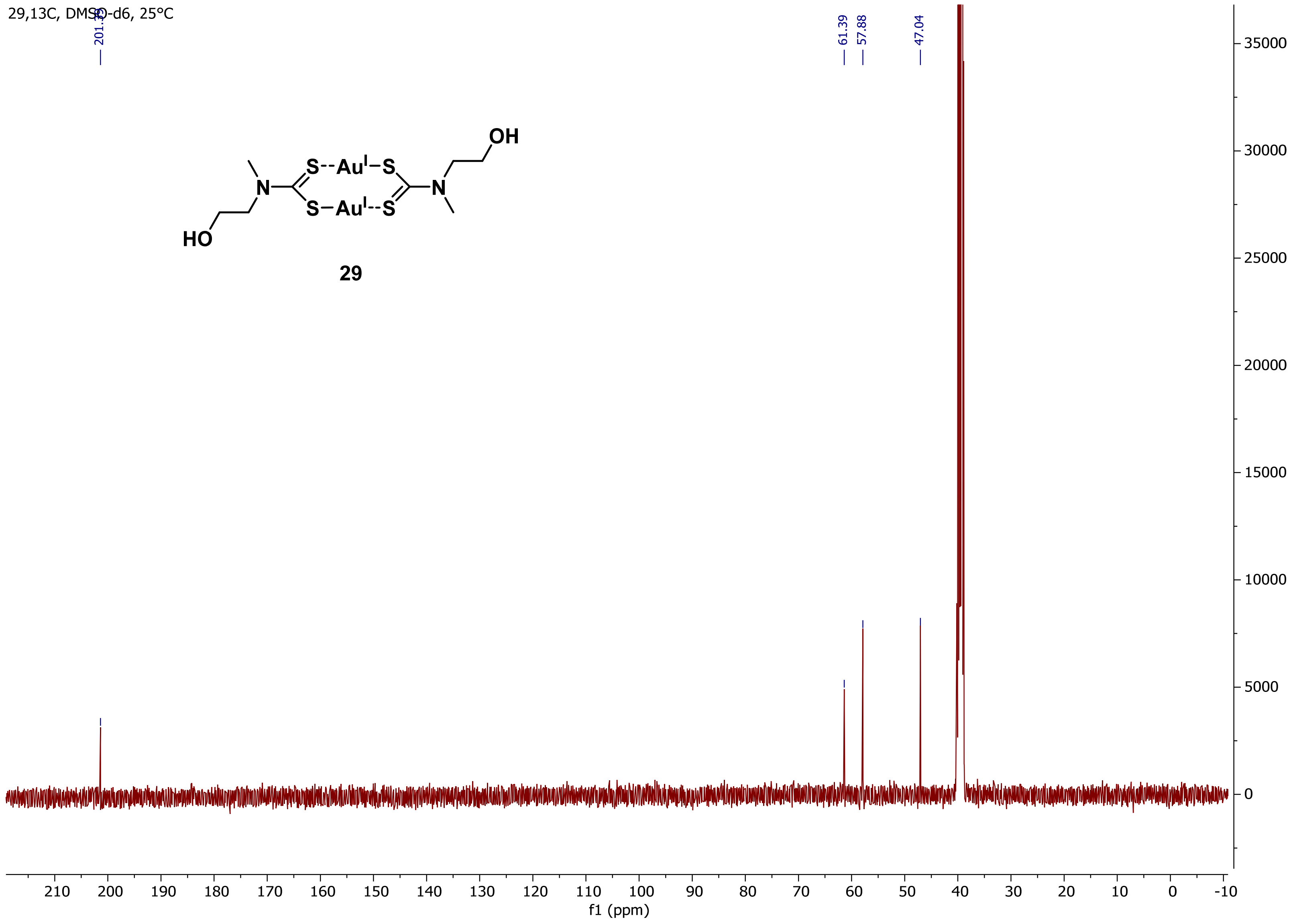
29,13C, DMSO-d6, 25°C
201.39
61.39
57.88
47.04
OH
S
Au
I
N
HO
29
35000
30000
25000
20000
15000
10000
5000
0
210 200 190 180 170 160 150 140 130 120 110 100 90 80 70 60 50 40 30 20 10 0 -10
f1 (ppm)

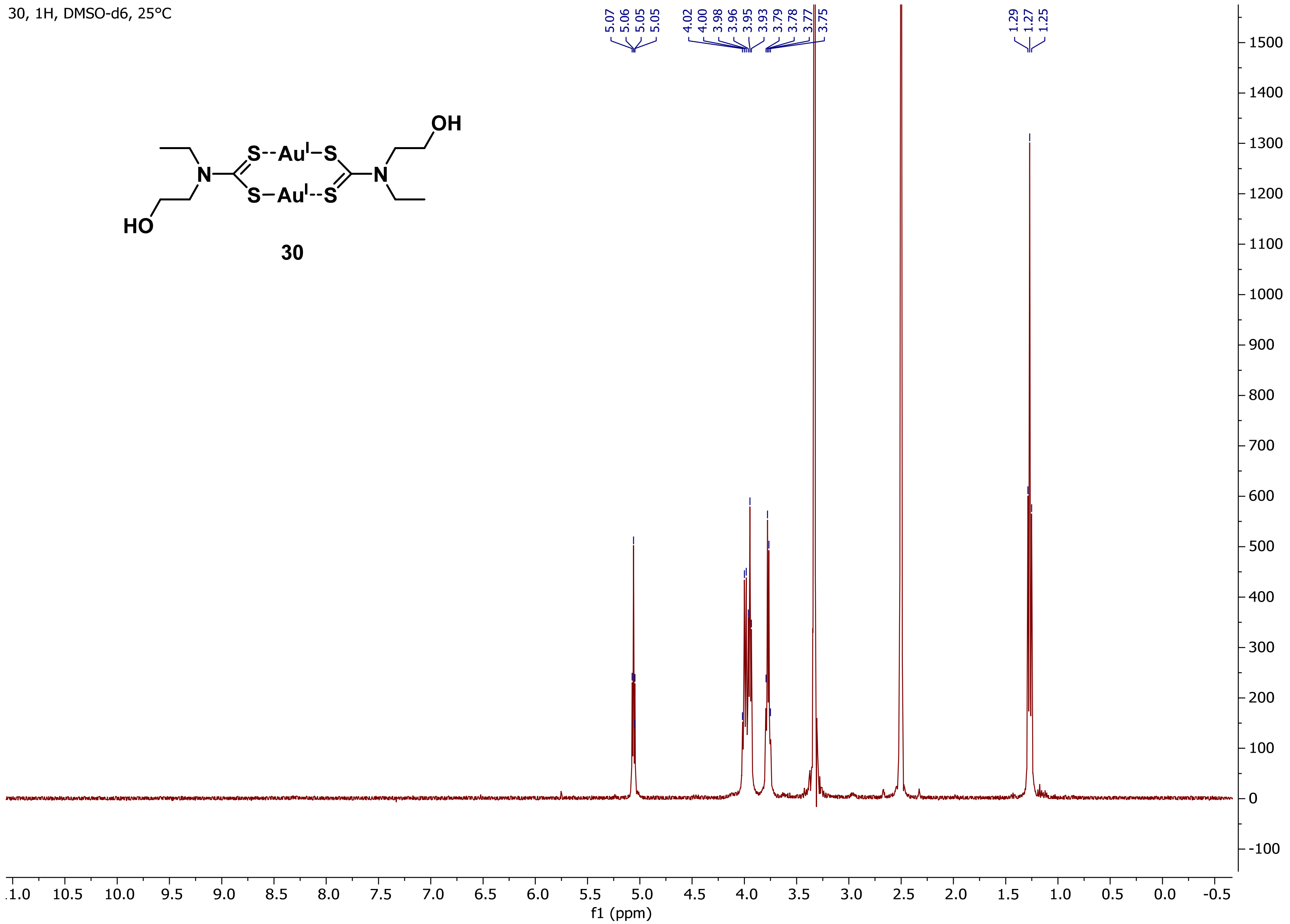
30, 1H, DMSO-d6, 25°C
OH
S--Au^I–S
N
S–Au^I--S
HO
30
5.07
5.06
5.05
5.05
4.02
4.00
3.98
3.96
3.95
3.93
3.79
3.78
3.77
3.75
1.29
1.27
1.25
f1 (ppm)

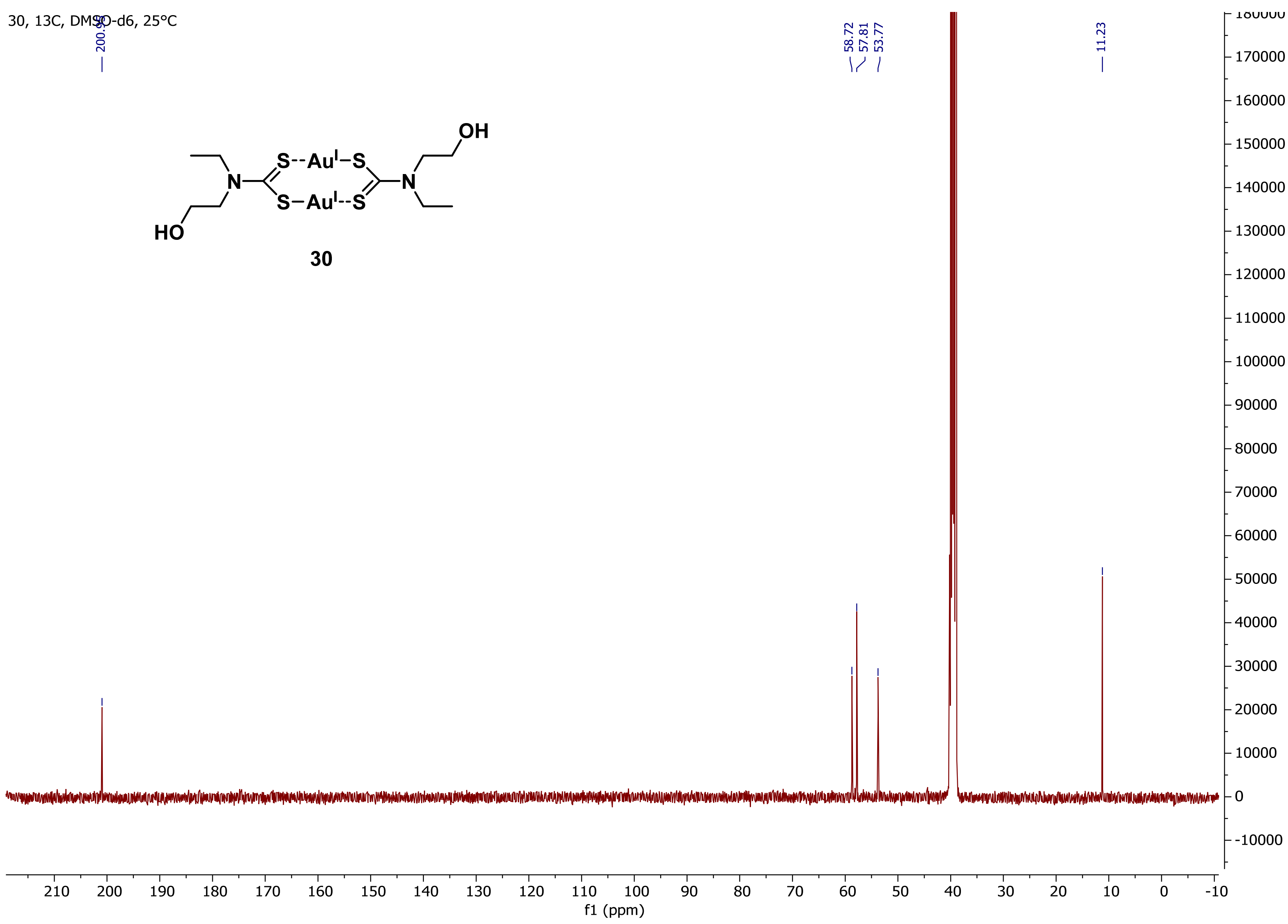
30, 13C, DMSO-d6, 25°C
200.93
58.72
57.81
53.77
11.23
OH
S--Au^{I}–S
N
S–Au^{I}--S
HO
30
f1 (ppm)

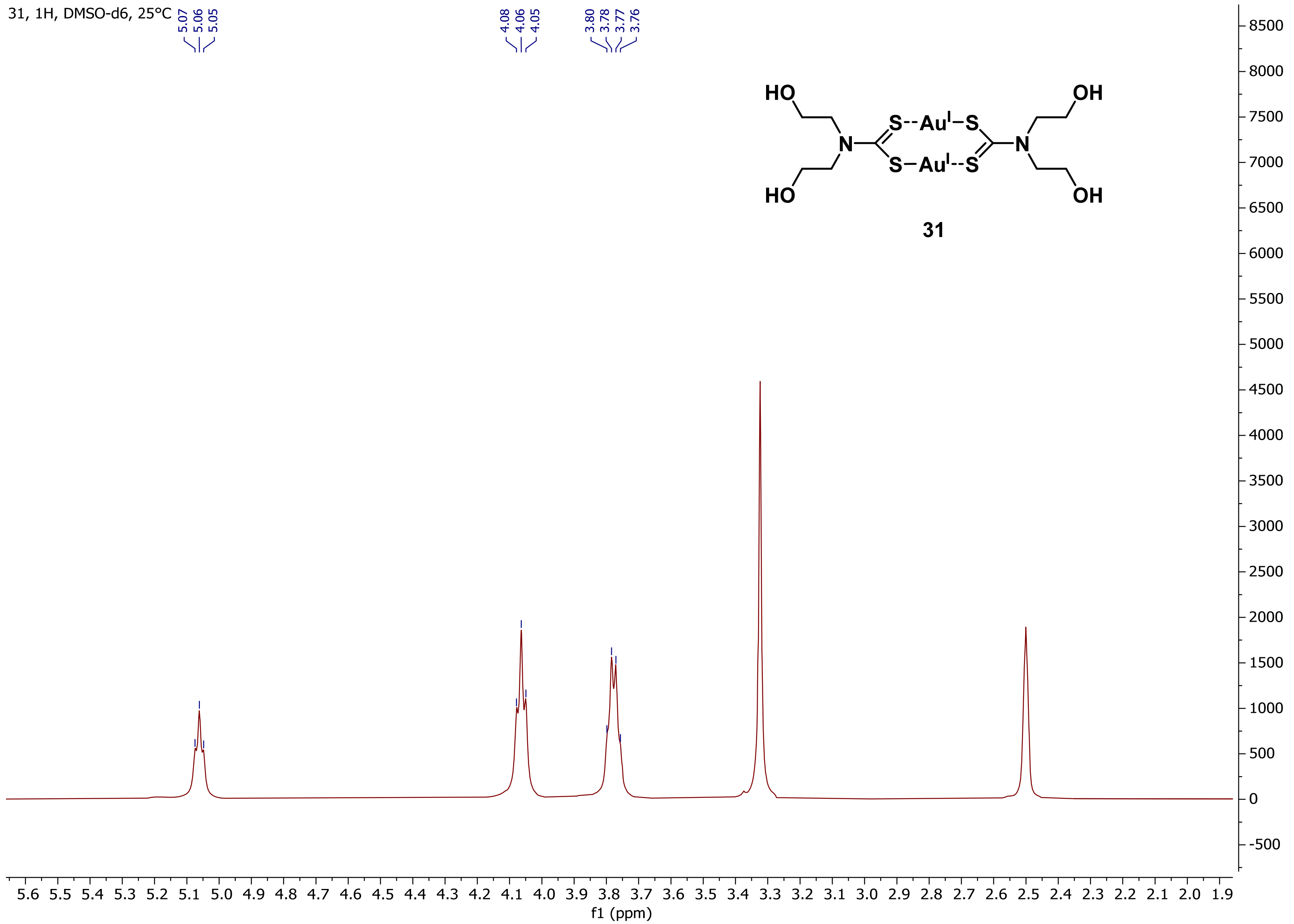

31, 1H, DMSO-d6, 25°C
5.07
5.06
5.05
4.08
4.06
4.05
3.80
3.78
3.77
3.76
HO
OH
HO
OH
N
N
S
S
S
S
Au
Au
31
f1 (ppm)

31, 13C, DMSO-d6, 25°C

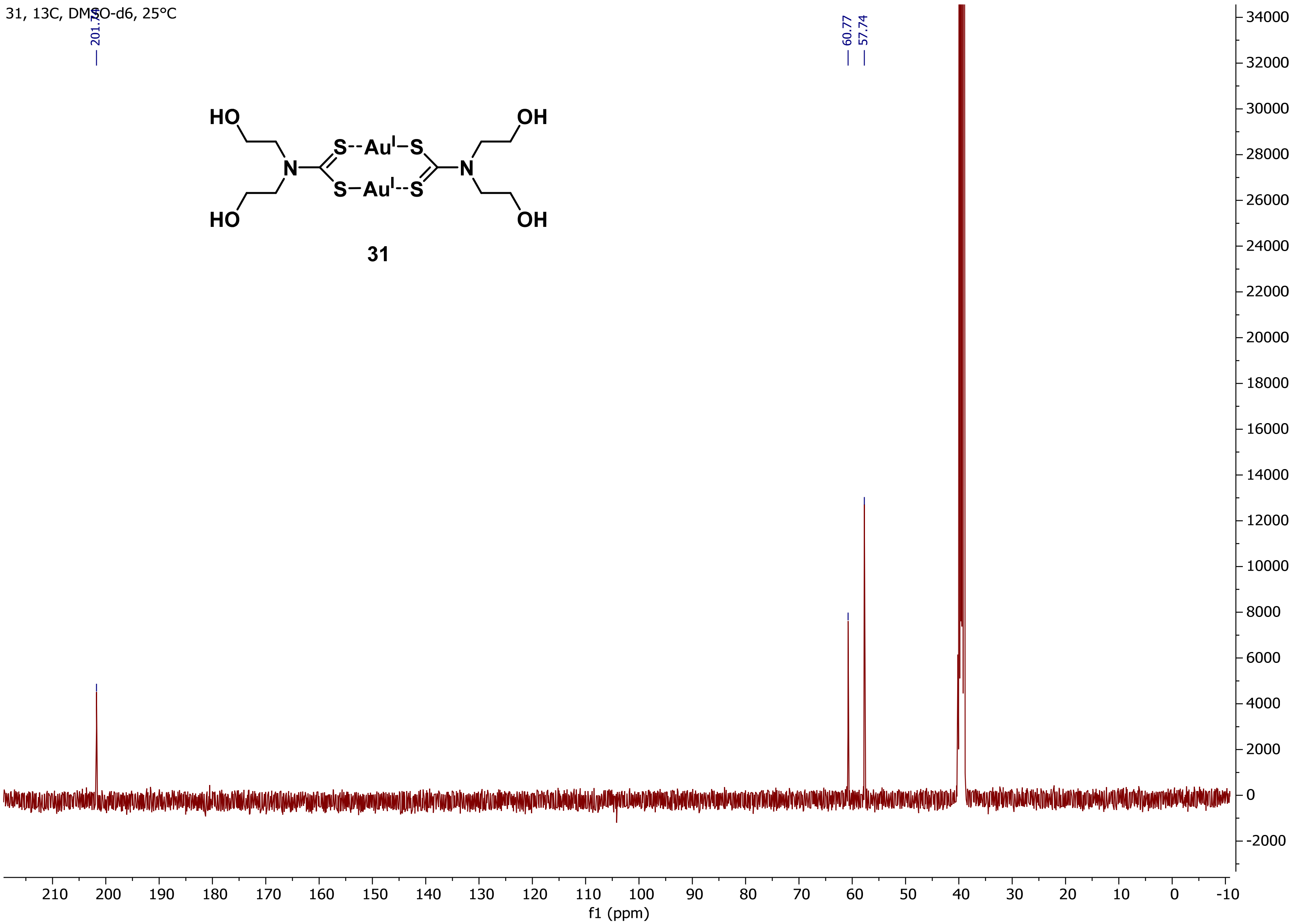

32, 1H, DMSO-d6, 25°C

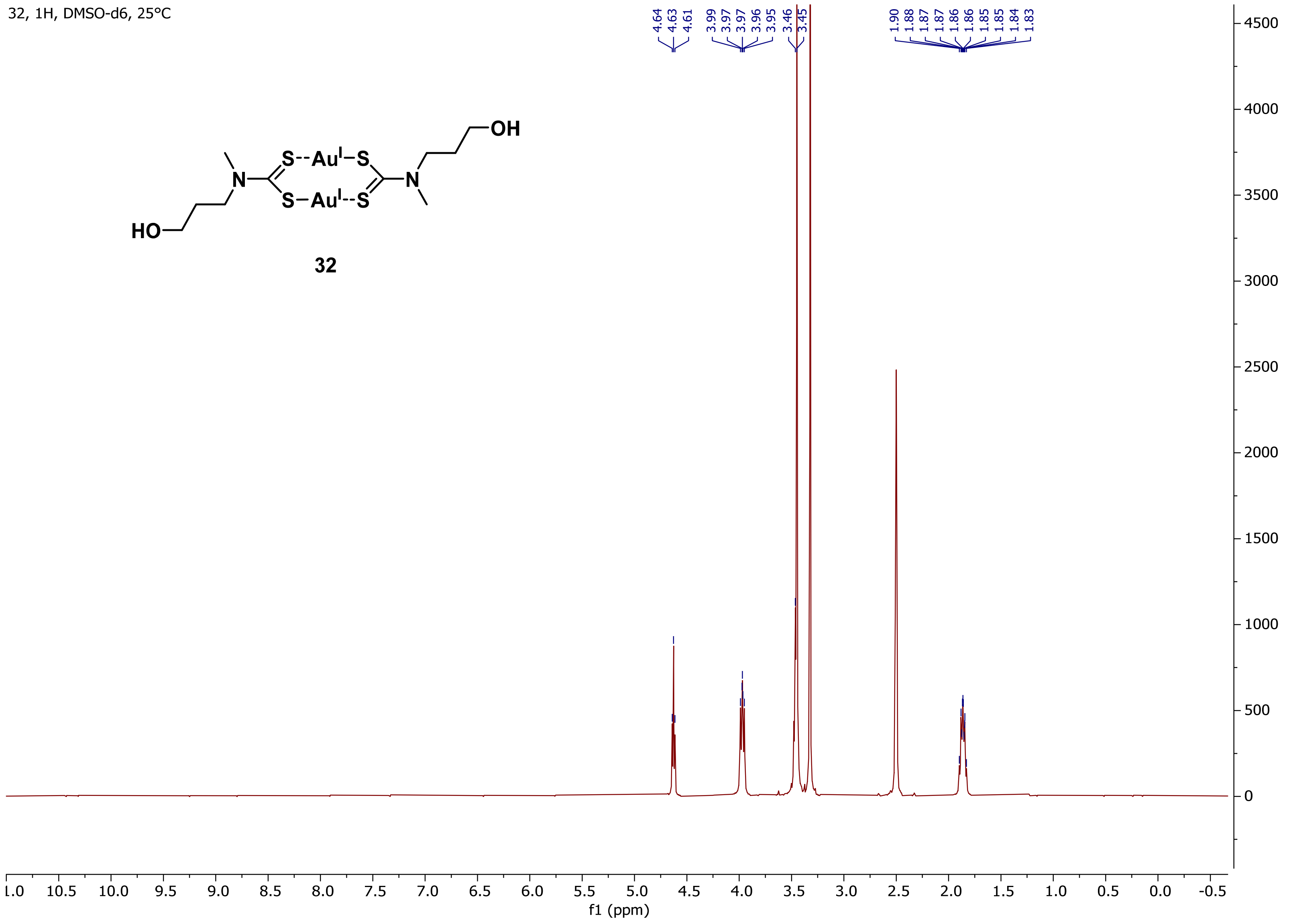

32

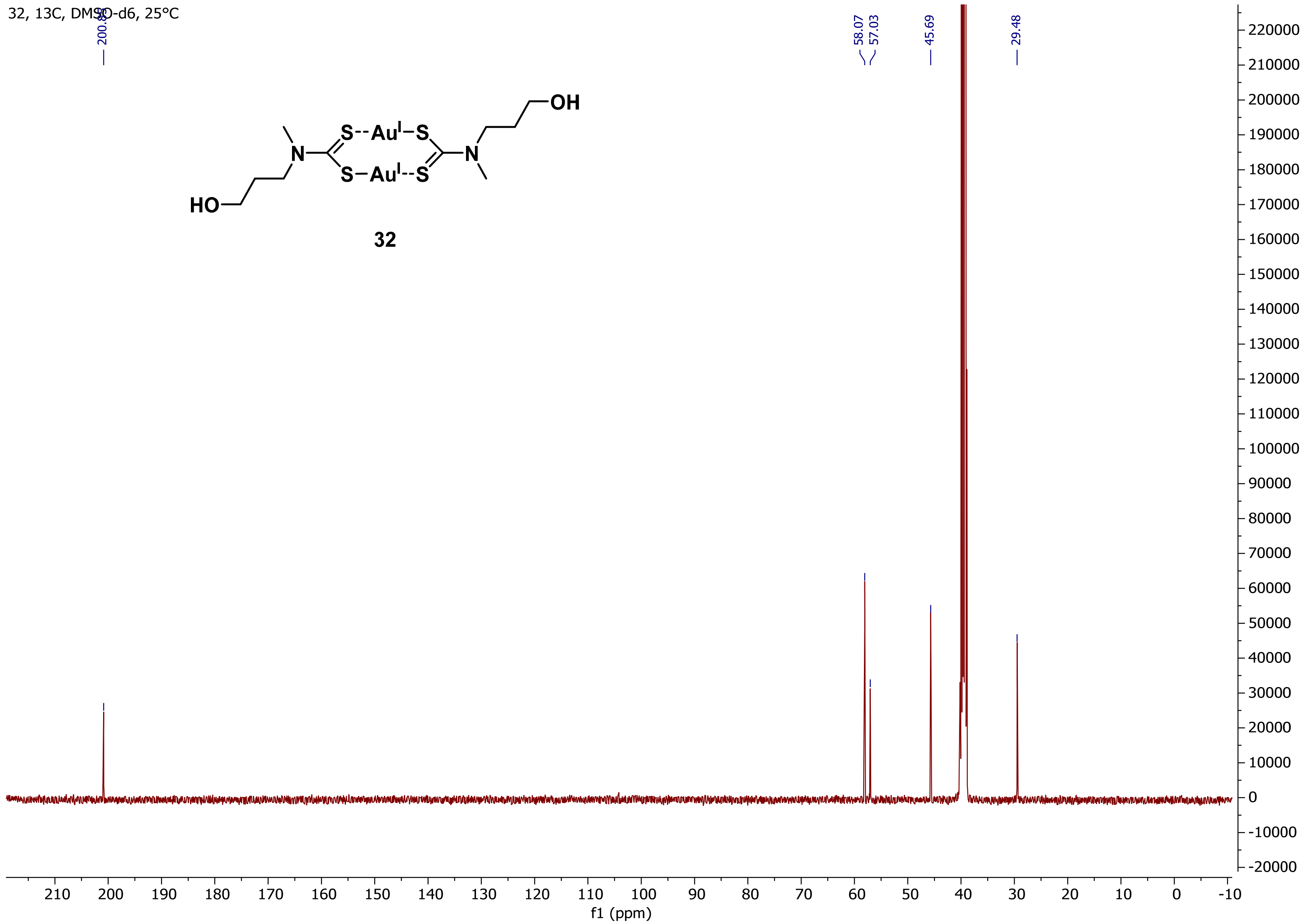

32, 13C, DMSO-d6, 25°C
58.07
57.03
45.69
29.48
HO
OH
N
S
Au^I
32
f1 (ppm)

33, 1H, DMSO-d6, 25°C

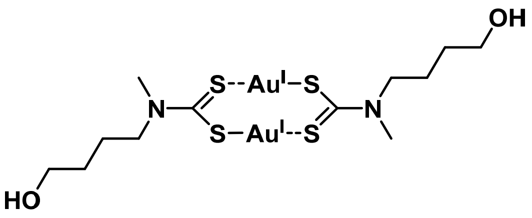

33

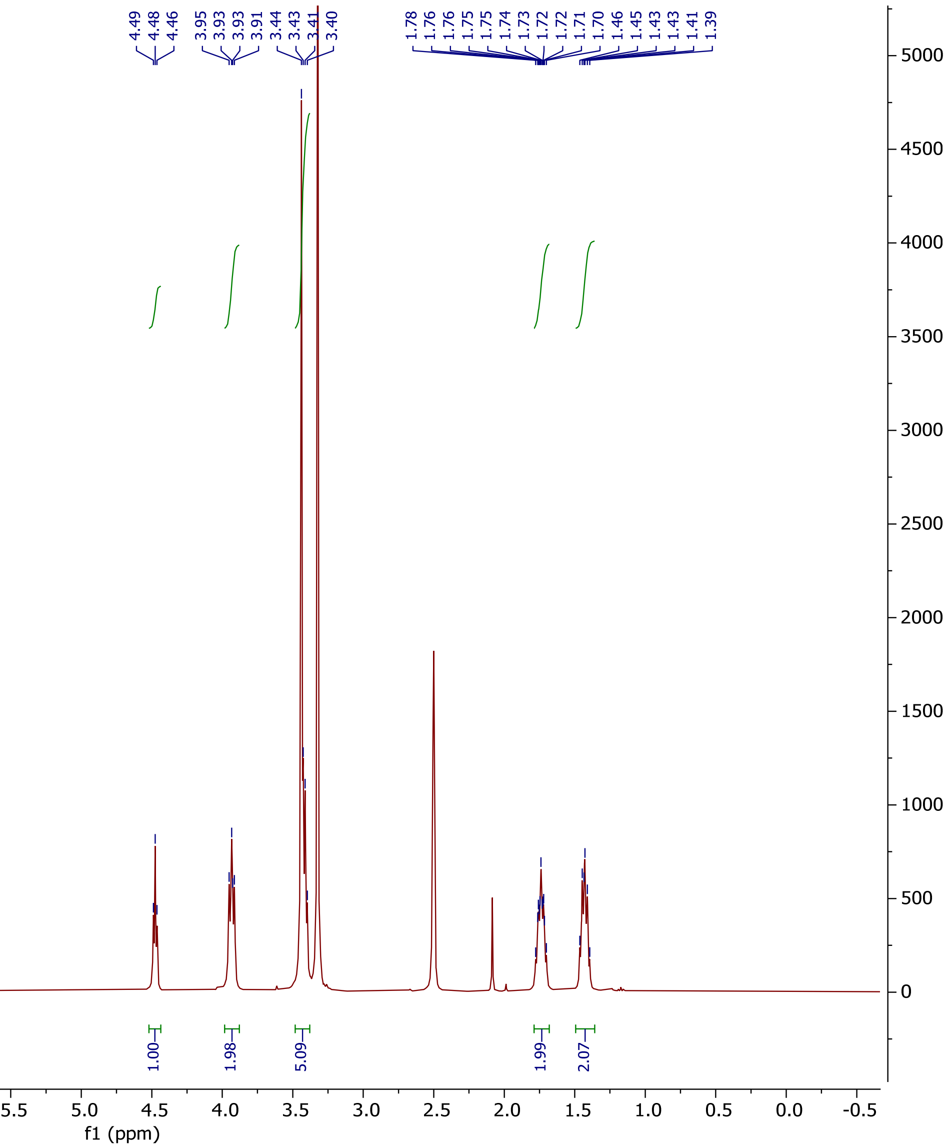

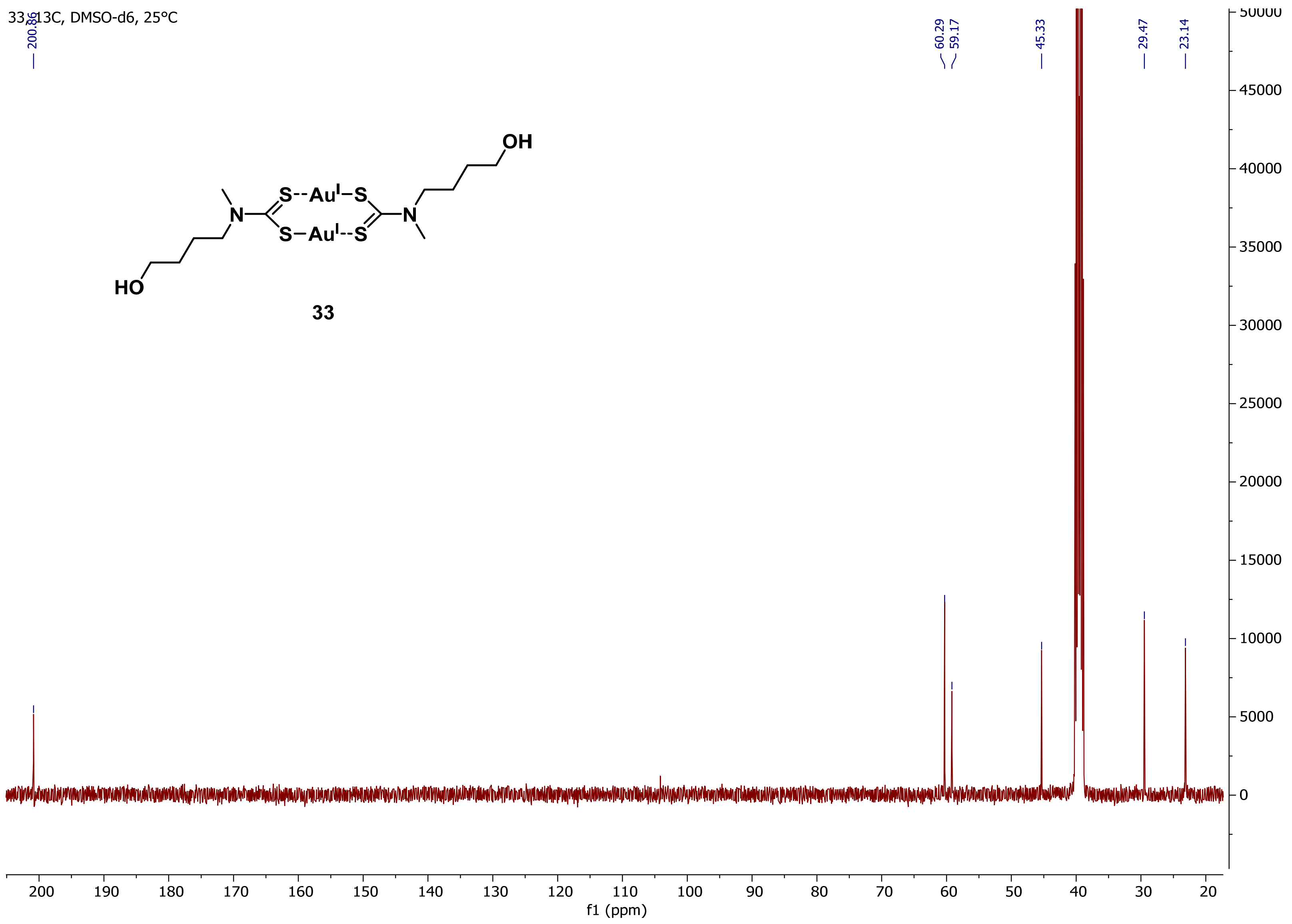
33, 13C, DMSO-d6, 25°C
200.86
60.29
59.17
45.33
29.47
23.14
OH
S--Au^I–S
N
S–Au^I--S
HO
33
50000
45000
40000
35000
30000
25000
20000
15000
10000
5000
0
200
190
180
170
160
150
140
130
120
110
100
90
80
70
60
50
40
30
20
f1 (ppm)

34, 1H, DMSO-d6, 25°C

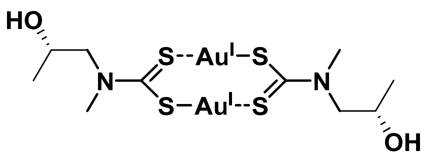

34

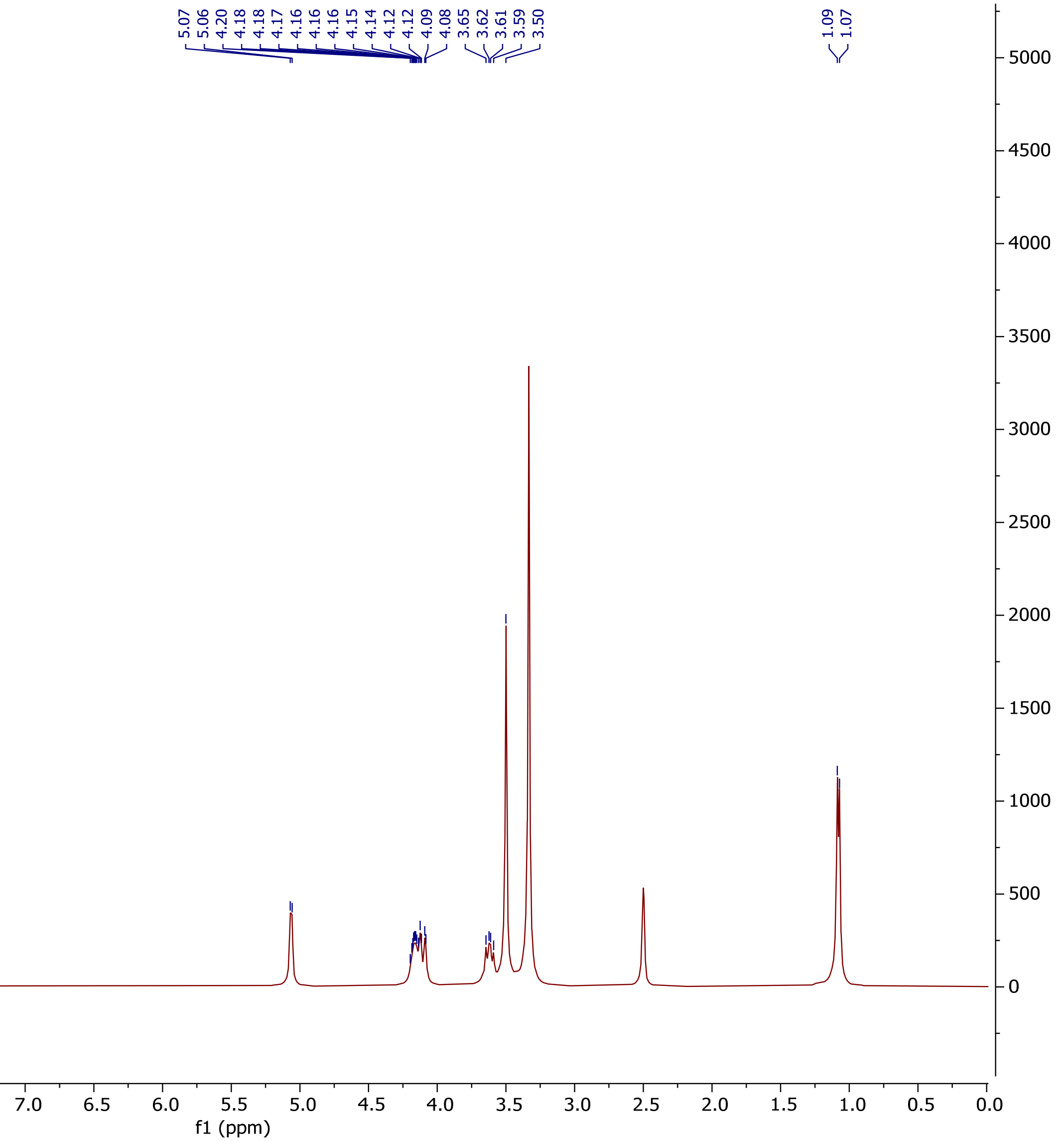

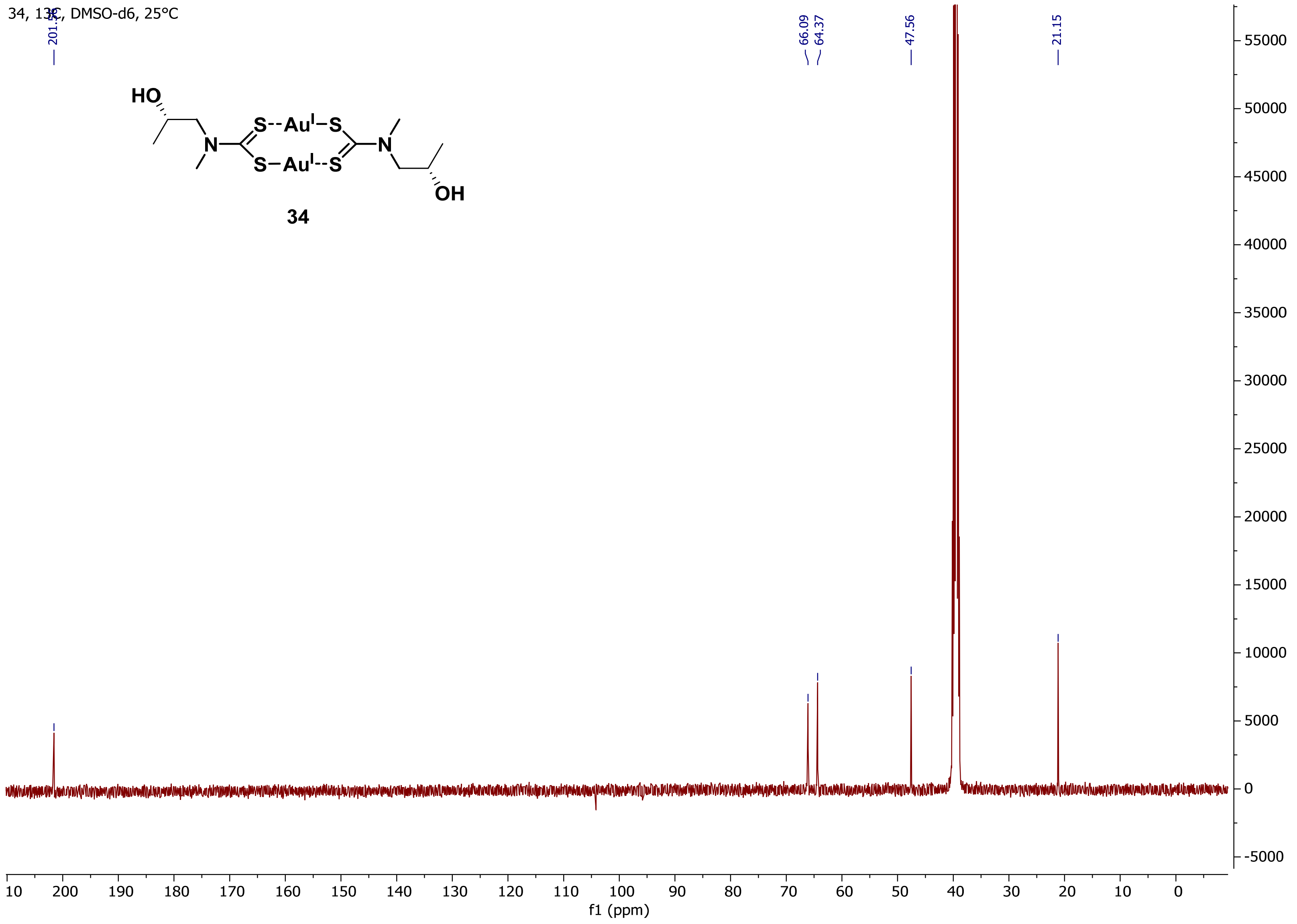
34, 13C, DMSO-d6, 25°C
201.5
66.09
64.37
47.56
21.15
HO
OH
S--Au^I–S
S–Au^I--S
N
34
f1 (ppm)
55000
50000
45000
40000
35000
30000
25000
20000
15000
10000
5000
0
-5000
10
200
190
180
170
160
150
140
130
120
110
100
90
80
70
60
50
40
30
20
10
0

35, 1H, DMSO-d6, 25°C

35

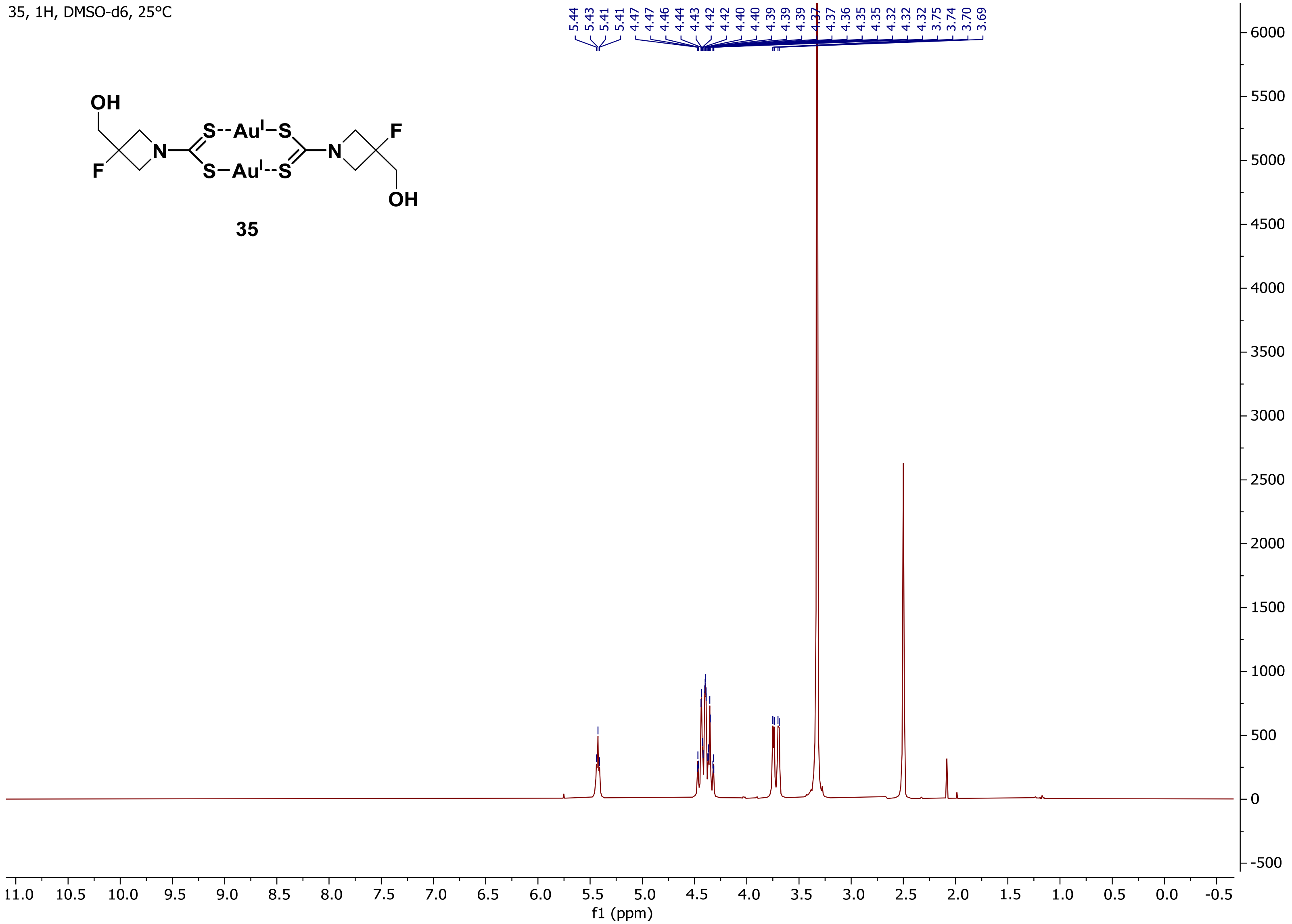

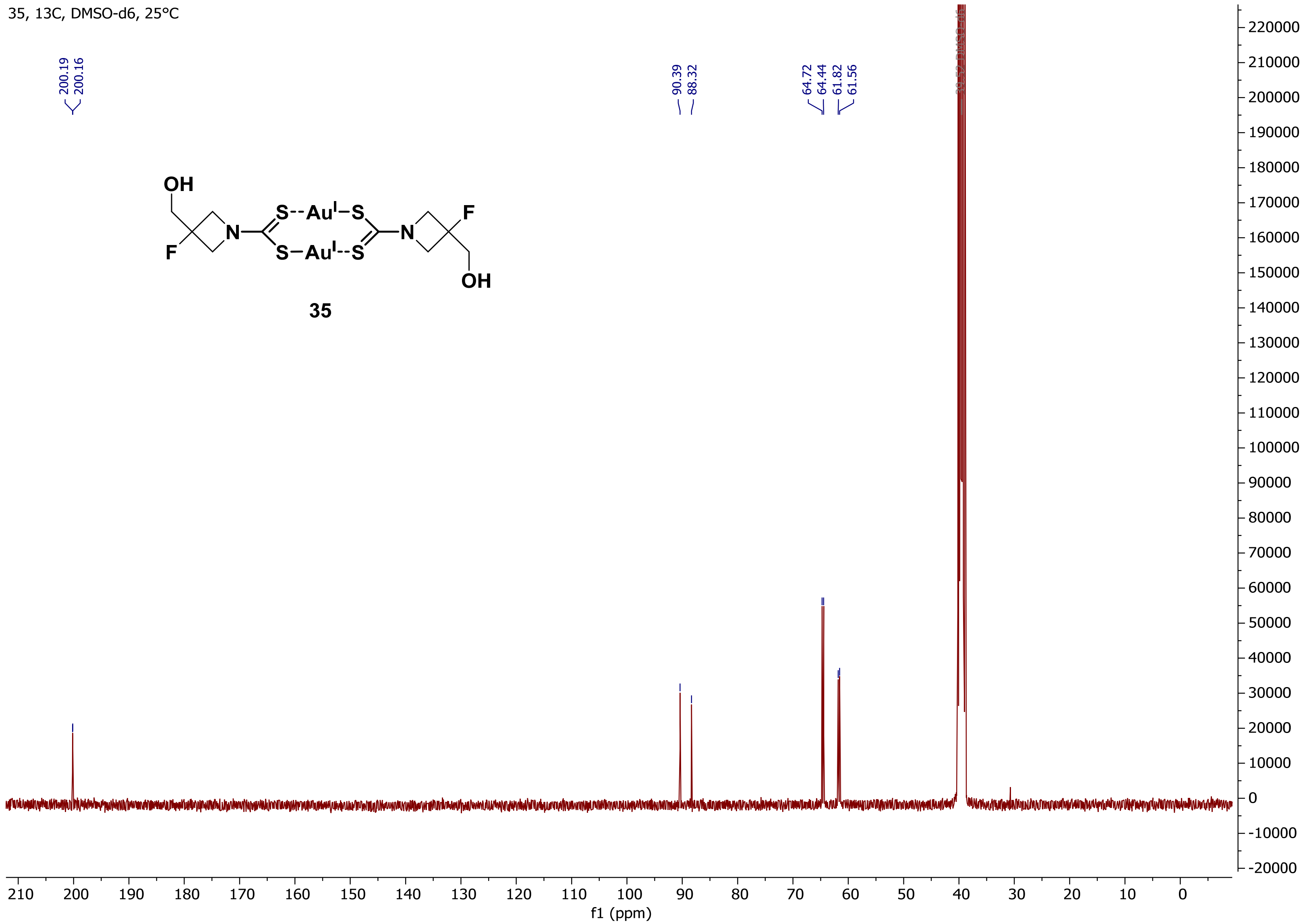
35, 13C, DMSO-d6, 25°C
200.19
200.16
90.39
88.32
64.72
64.44
61.82
61.56
35
f1 (ppm)

36, 1H, DMSO-d6, 25°C

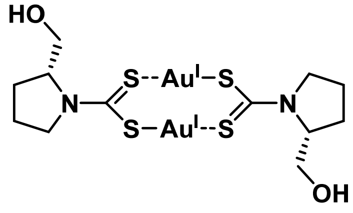

36

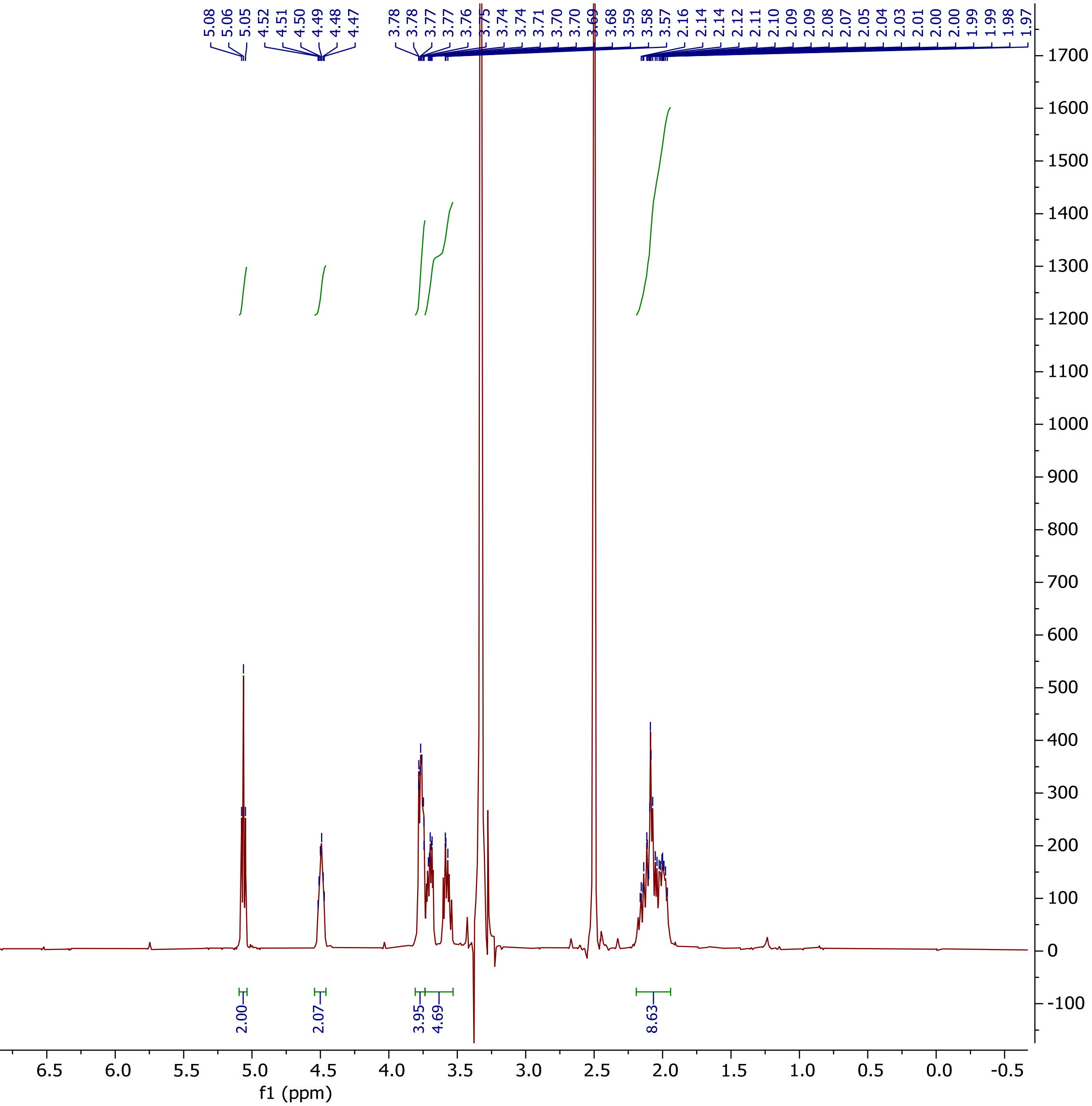

36, 13C, DMSO-d6, 25°C

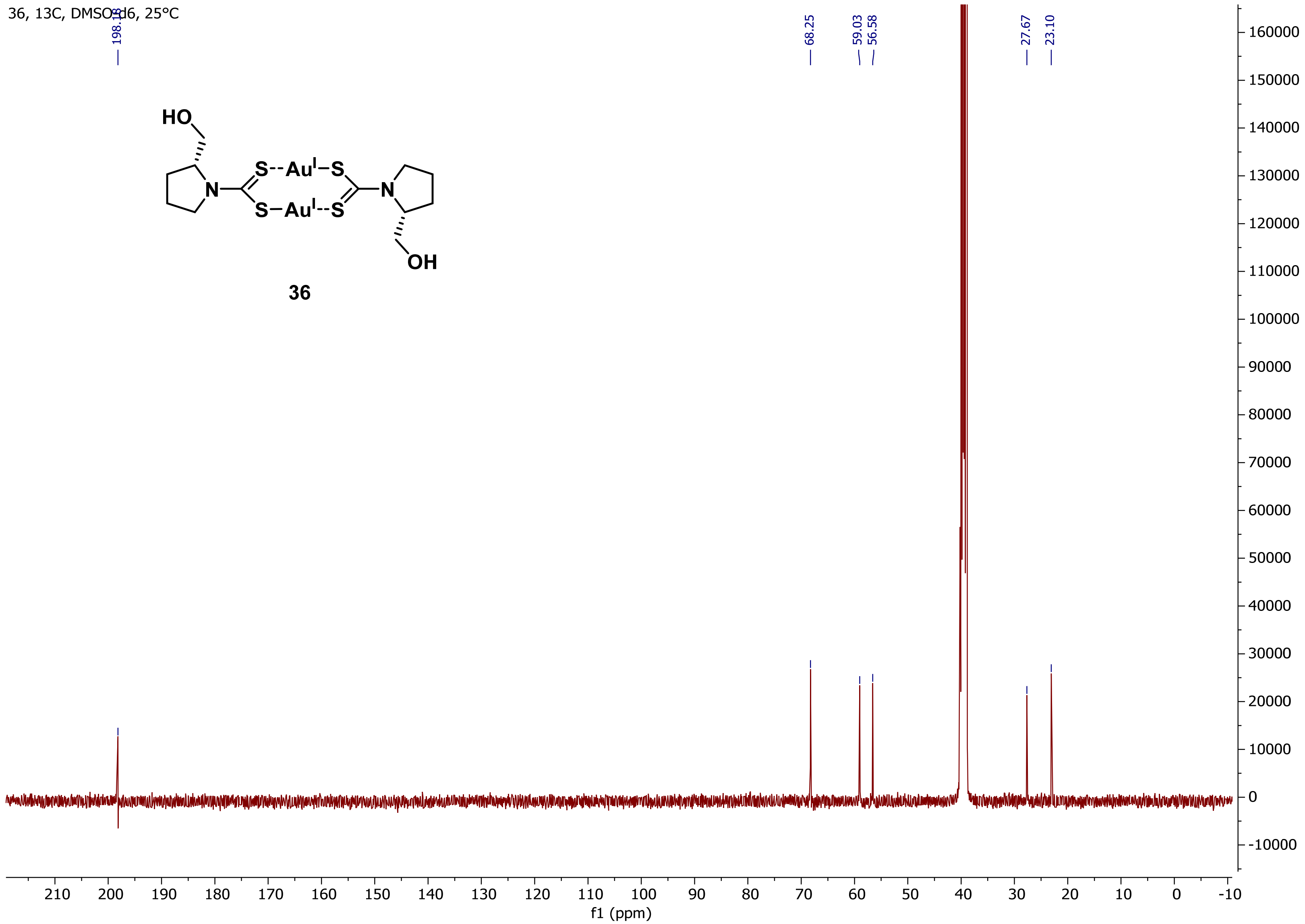

37, 1H, DMSO-d6, 25°C

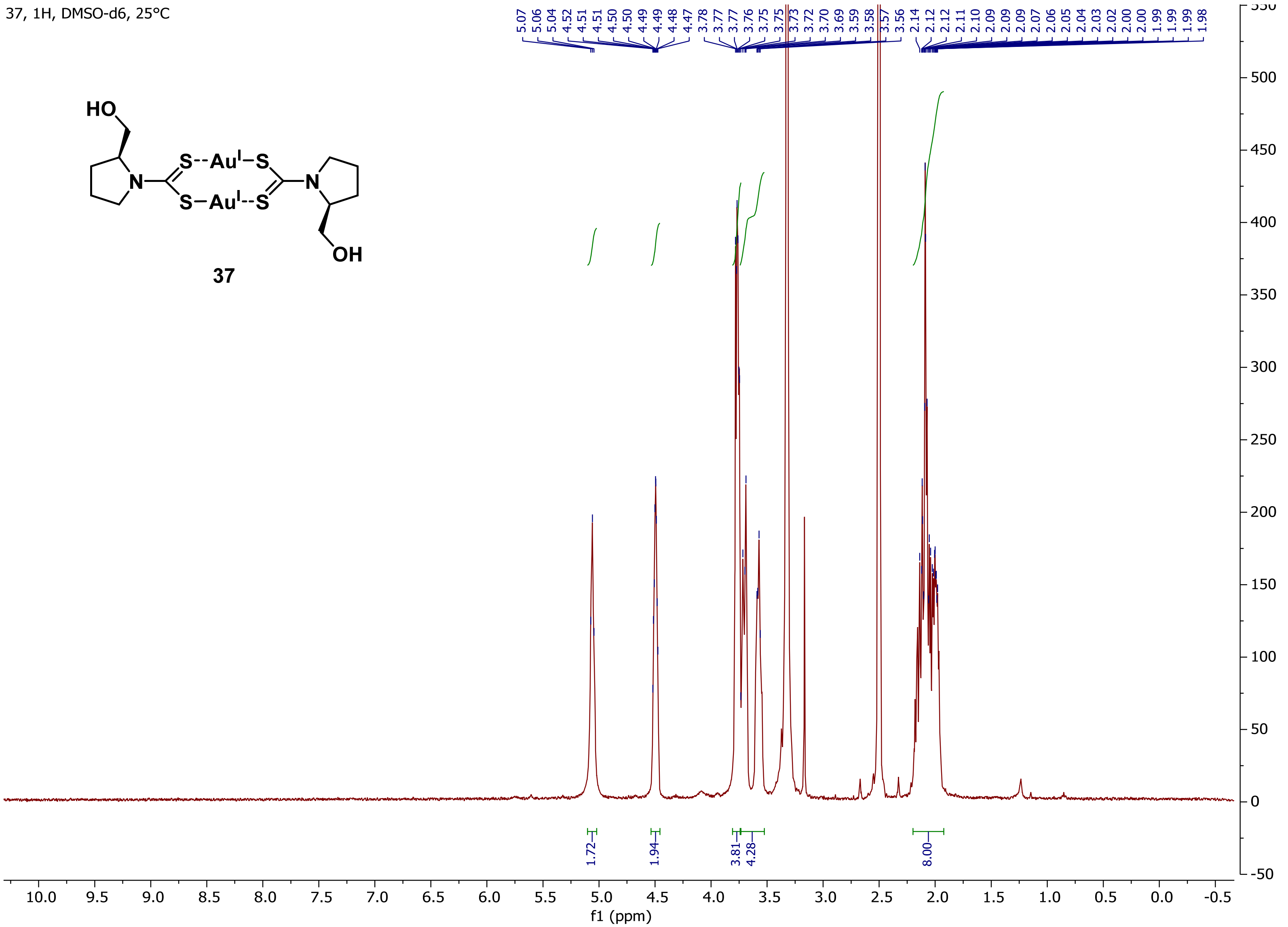

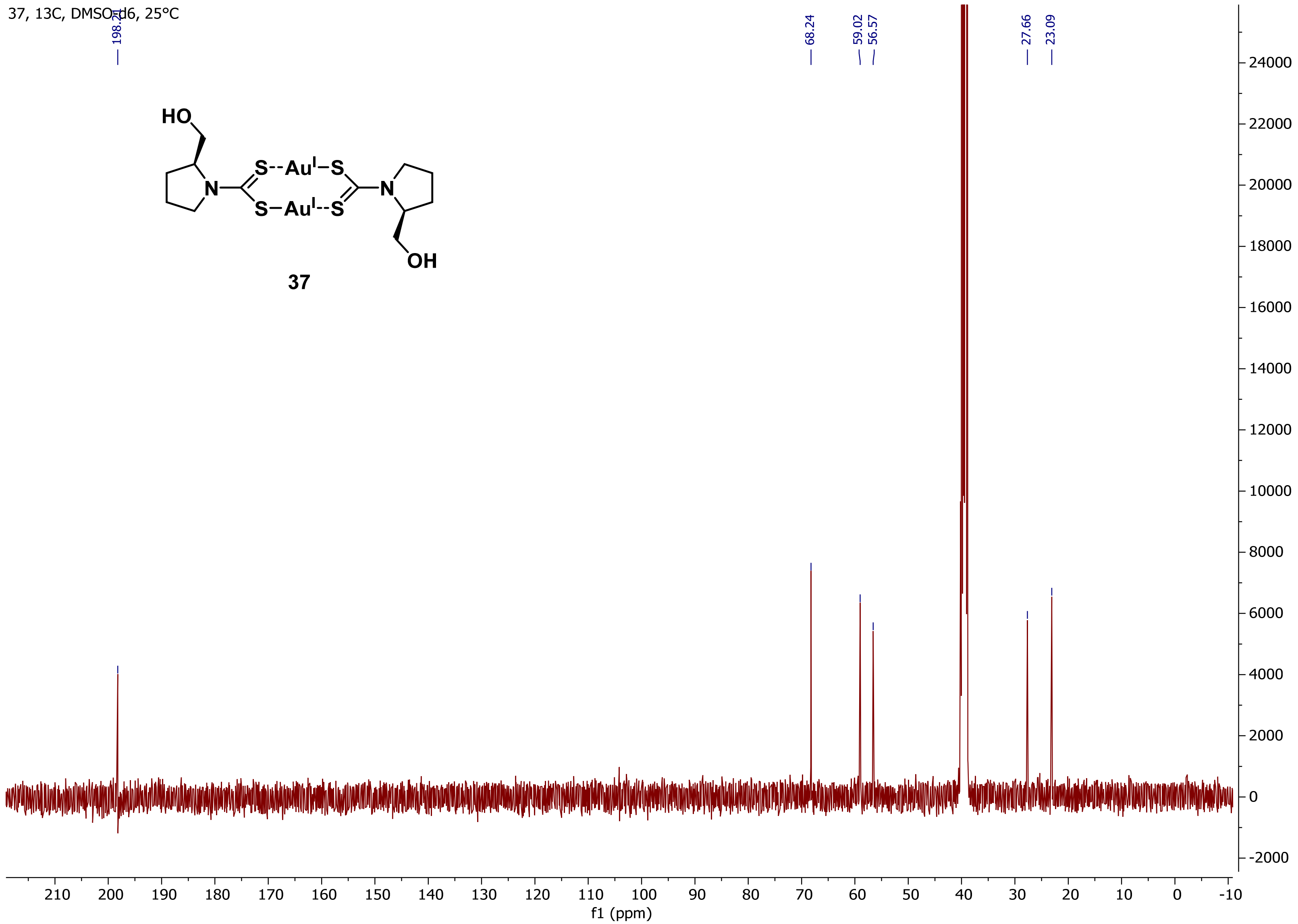
37, 13C, DMSO-d6, 25°C
198.21
68.24
59.02
56.57
27.66
23.09
HO
S
Au
N
OH
37
f1 (ppm)

38, 1H, DMSO-d6, 25°C

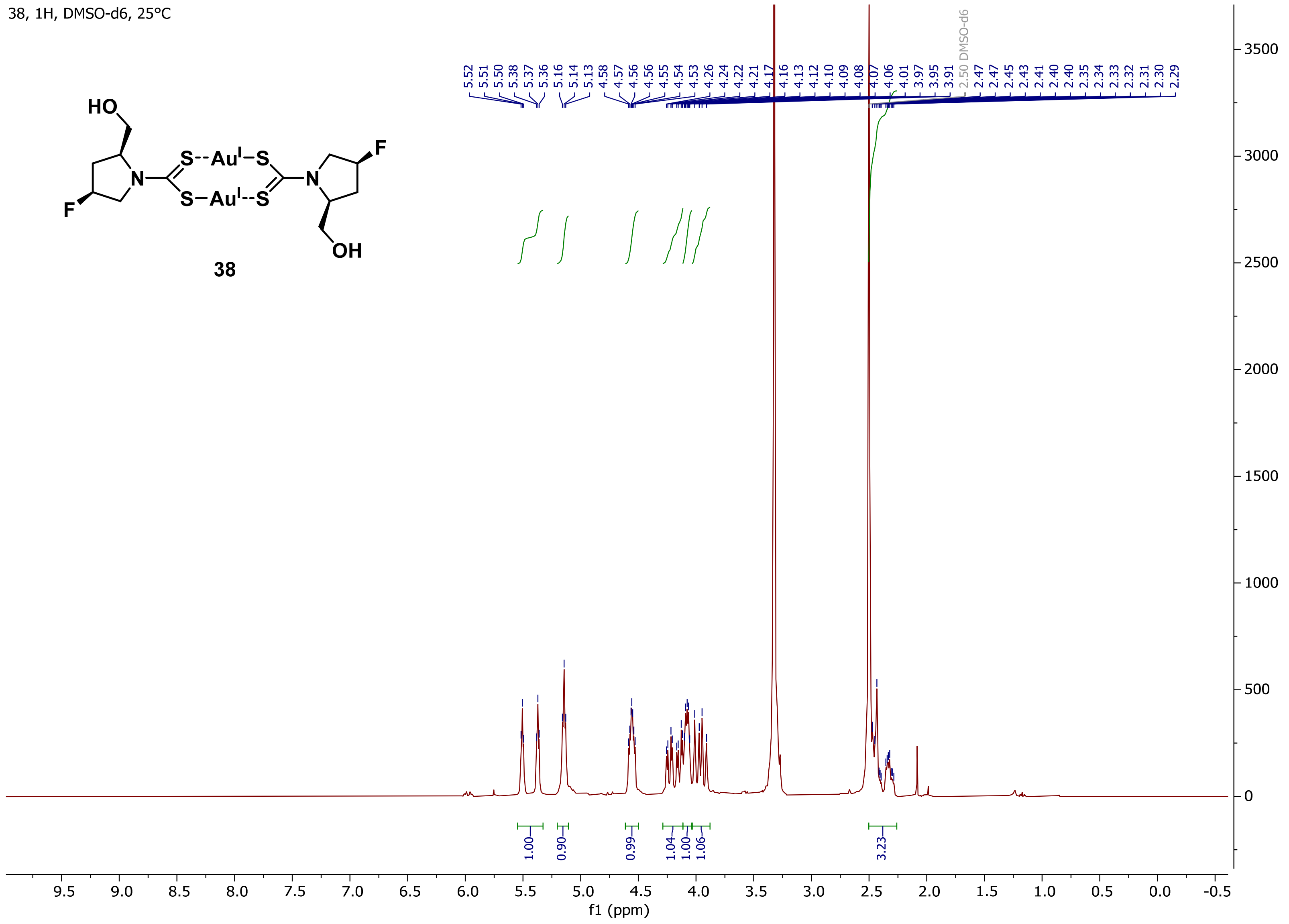

38, 13C, DMSO-d6, 25°C

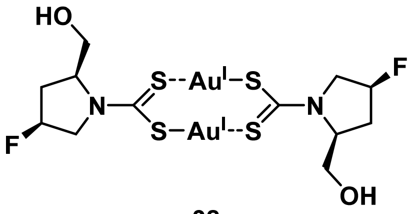

38

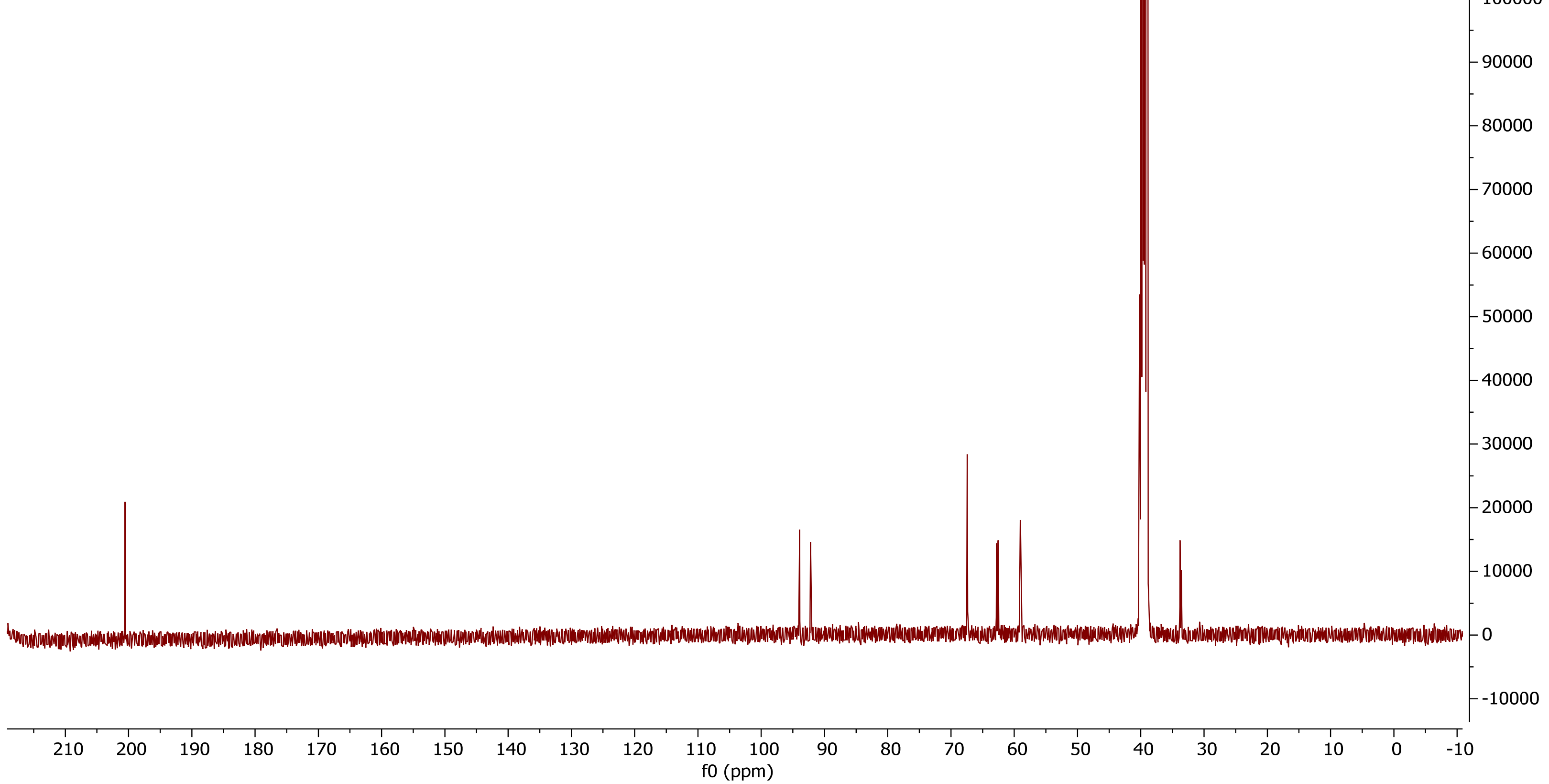

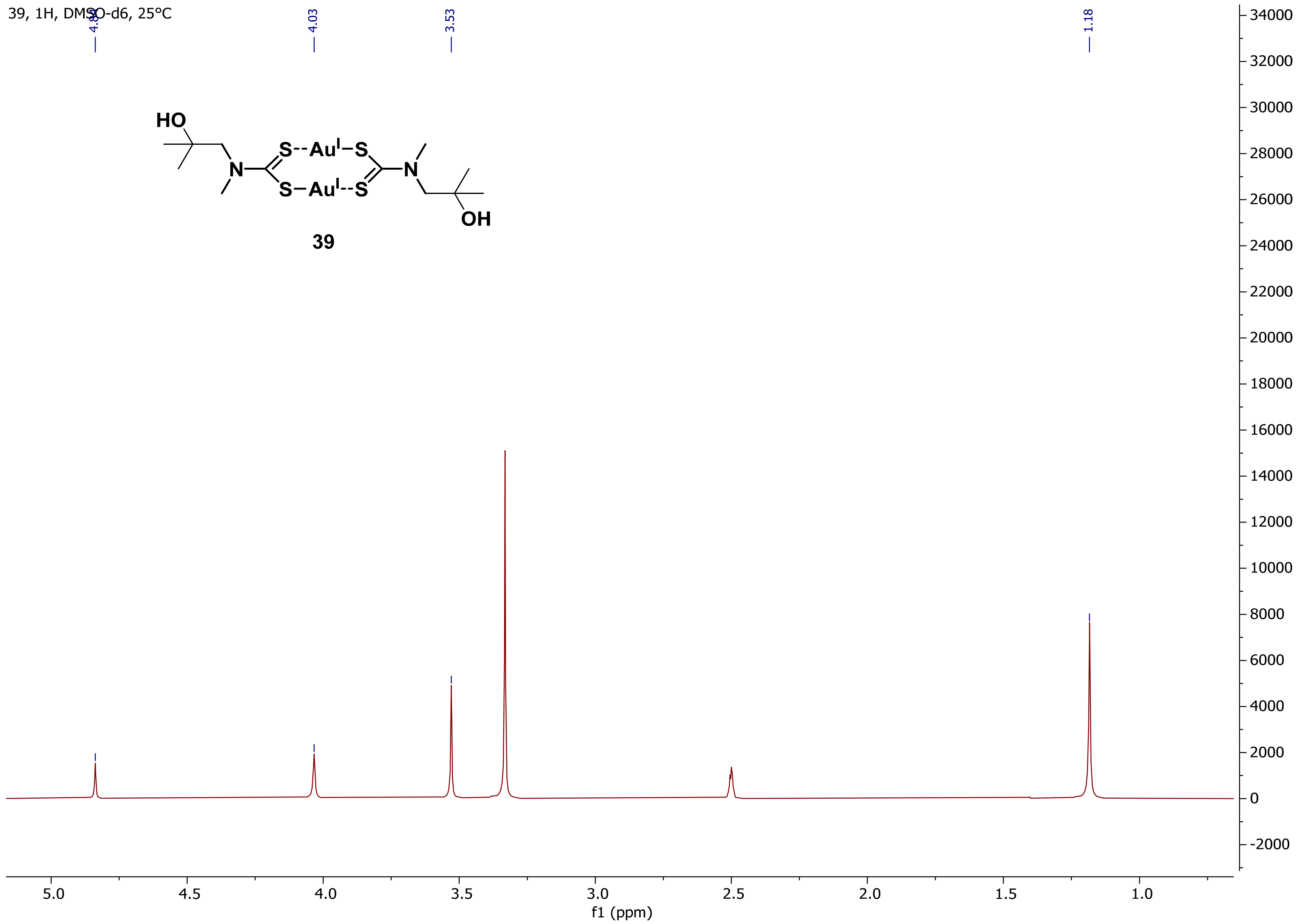
39, 1H, DMSO-d6, 25°C
4.03
3.53
1.18
HO
S
Au
S
N
OH
39
f1 (ppm)

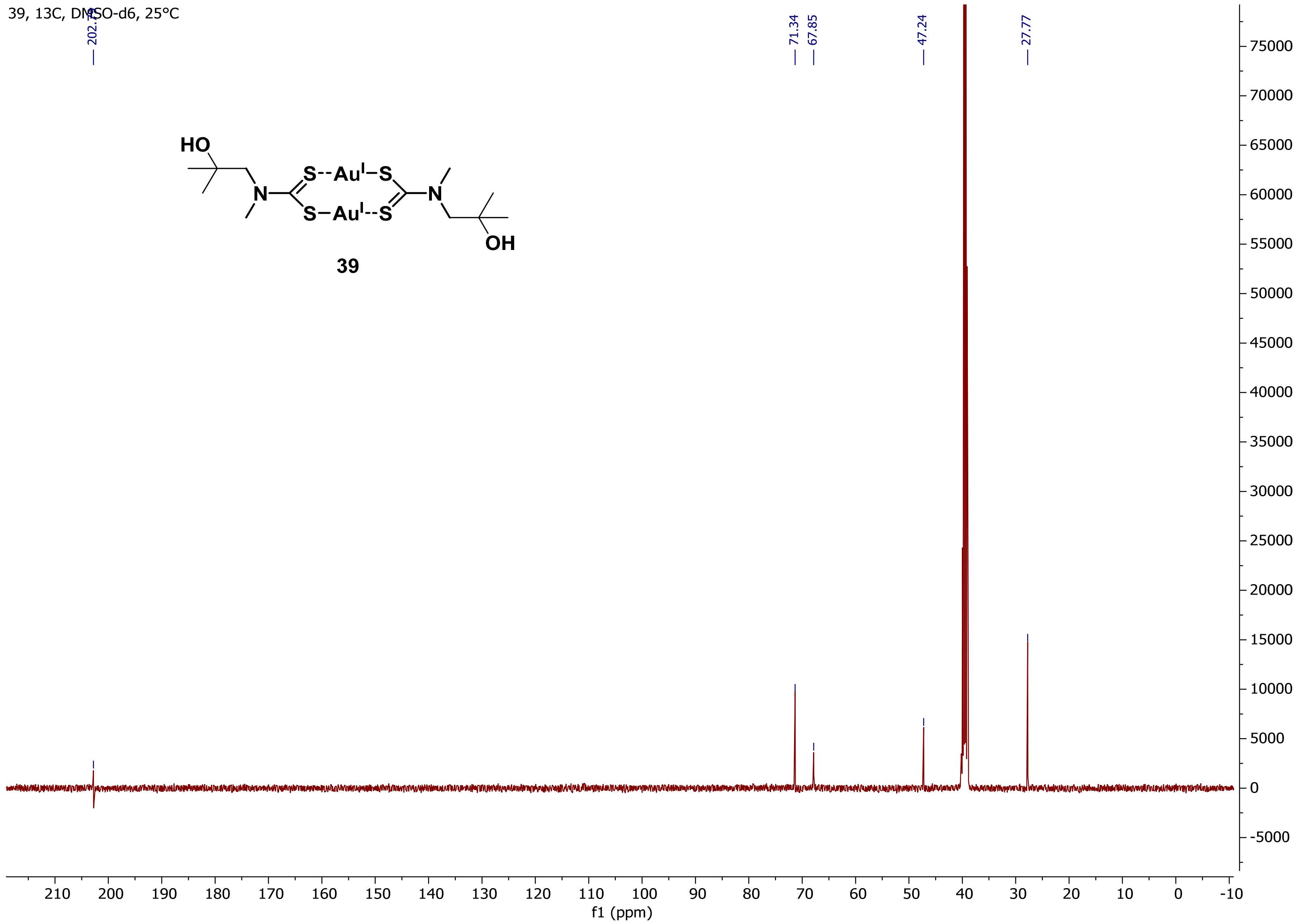
39, 13C, DMSO-d6, 25°C
71.34
67.85
47.24
27.77
HO
OH
S
Au
N
39
f1 (ppm)
210 200 190 180 170 160 150 140 130 120 110 100 90 80 70 60 50 40 30 20 10 0 -10
75000 70000 65000 60000 55000 50000 45000 40000 35000 30000 25000 20000 15000 10000 5000 0 -5000
